# Piezo1 activation suppresses bone marrow adipogenesis to prevent osteoporosis by inhibiting a mechanoinflammatory autocrine loop

**DOI:** 10.1038/s41392-025-02455-w

**Published:** 2025-10-28

**Authors:** Baile Wang, Jie Liu, Qin Wang, Malika Arhatte, Lai Yee Cheong, Edyta Glogowska, Xue Jiang, Sookja Kim Chung, Leigang Jin, Qianxing Hu, Yu Wang, Eric Honoré, Aimin Xu

**Affiliations:** 1 State Key Laboratory of Pharmaceutical Biotechnology, The University of Hong Kong, Hong Kong, China; 2 Department of Medicine, The University of Hong Kong, Hong Kong, China; 3 Guangdong-Hong Kong Joint Laboratory for Metabolic Medicine, The University of Hong Kong, Hong Kong, China; 4 Université Côte d’Azur, Centre National de la Recherche Scientifique, Institut national de la santé et de la recherche médicale, Institut de Pharmacologie Moléculaire et Cellulaire, Labex ICST, Valbonne, France; 5 Faculty of Medicine; Dr. Neher’s Biophysics Laboratory for Innovative Drug Discovery, State Key Laboratory of Quality Research in Chinese Medicine, Macau University of Science and Technology, Macao, China; 6 Department of Pharmacology & Pharmacy, The University of Hong Kong, Hong Kong, China

**Keywords:** Stem-cell differentiation, Mesenchymal stem cells, Endocrine system and metabolic diseases, Bone development

## Abstract

With aging or osteoporosis, bone marrow adipogenesis is increased and inversely correlates with the loss of bone mass. Bone marrow adipocytes are derived from multipotent bone marrow mesenchymal stem cells (BMMSCs), which can differentiate into either fat or bone. BMMSCs are mechanosensitive cells, but how mechanical loading is implicated in the in vivo regulation of bone marrow adipogenesis and its impact on bone remodeling remain poorly understood. Here, we identify the mechanosensitive cationic channel Piezo1 in BMMSCs as a key suppressor of bone marrow adipogenesis by preventing local inflammation, thereby enhancing osteoblast differentiation and bone formation. Mice with a specific Piezo1 invalidation in BMMSCs exhibit osteoporosis and marrow adiposity, together with resistance to the beneficial effects of exercise on bone health. Accordingly, Piezo1-deficient BMMSCs in vitro preferentially differentiate into adipocytes rather than osteoblasts. Invalidation of Piezo1 in BMMSCs enhances the autocrine activation of CCR2 by Ccl2, which further induces lipocalin-2 (Lcn2) production via NF-κB activation, thereby promoting adipocyte differentiation. Conversely, Piezo1 opening induces Klf2 expression through CaMKII, preventing c-Jun activation, Ccl2 production and bone marrow adipogenesis. These findings demonstrate that Piezo1 activation in BMMSCs suppresses bone marrow adipogenesis to maintain bone strength by preventing the Ccl2-Lcn2 inflammatory autocrine loop, thus uncovering a previously unrecognized link between mechanotransduction, inflammation, and cell fate determination.

## Introduction

Mesenchymal stem cells (MSCs) located within the bone marrow stroma are nonhematopoietic multipotent stem cells that can differentiate into several cell types, including osteoblasts or adipocytes. As multipotent stem cells, the lineage commitment of bone marrow MSCs (BMMSCs) has garnered considerable attention due to its significant implications for regenerative medicine and tissue engineering.^1^ The tightly controlled lineage commitment of BMMSCs into osteoblasts or adipocytes is competitive and reciprocal, which forms a balance to maintain a homeostatic state.^2^ Disruption of this fine balance is linked to a variety of pathophysiological conditions, such as aging, obesity, cancer, and osteoporosis.^2^ The regulation of BMMSC differentiation is a complex process that involves various signaling pathways, transcription factors, and extracellular matrix components.^2^ The transcription factors peroxisome proliferator-activated receptor gamma (PPARγ) and CCAAT/enhancer binding protein alpha (C/EBPα) are two key players in adipogenesis,^3^ while RUNX family transcription factor 2 (RUNX2) and Osterix are crucial for osteogenic differentiation of BMMSCs.^4^ Furthermore, adipogenesis and osteogenesis of BMMSCs are finely balanced by their reciprocal regulation. Adipocyte-derived factors inhibit osteogenesis,^5^ whereas factors secreted by osteoblasts suppress adipogenesis.^6^ Additionally, a number of external cues, including physical factors, cytokines, chemokines, growth factors, hormones, and extracellular matrix components present in the bone marrow niche, trigger different signaling pathways in BMMSCs to influence the lineage commitment of BMMSCs.^2^

BMMSCs are mechanosensitive, and the lineage commitment of BMMSCs is greatly influenced by a variety of mechanical stimuli, including fluid shear stress, tension, compression, and substrate rigidity.^7^ However, how mechanical parameters influence the biology of BMMSCs is not yet fully understood. In vitro evidence has shown that mechanical stimuli, such as fluid shear stress, tensile strain, and hydrostatic pressure, promote osteogenesis while inhibiting adipogenesis in BMMSCs.^8–10^ Conversely, mechanical unloading favors the differentiation of BMMSCs into adipocytes.^11^ Therefore, deciphering the underlying mechanisms whereby BMMSCs transduce mechanical stimuli into intracellular biochemical signals to modulate lineage commitment may shed new light on the development of stem cell-based therapeutic strategies. To date, various force-sensitive ion channels, including TREK/TRAAK K_2P_ channels, Piezo1/2, TMEM63/OSCA, and TMC1/2, have been identified in mammalian cells.^12^ However, the specific role of these mechanosensitive ion channels in the regulation of BMMSC differentiation remains to be determined.

Piezo1 and Piezo2 are nonselective cationic channels that are exquisitely activated by mechanical stimulation, including shear stress and pressure-induced membrane stretching.^12,13^ Piezo1 is a large trimeric complex with a large N-terminal mechanotransduction module (forming blades) followed by a C-terminal ionic pore.^14,15^ It exhibits a distinctive topology with 38-transmembrane-helix and specialized mechanotransduction components, which collectively facilitate a lever-like mechanogating mechanism.^16^ At the closed state, Piezo1 is curved to form an inverted dome that flattens upon mechanical activation.^17^ Piezo1 is broadly expressed in peripheral tissues and is critically required for vascular and lymphatic development, as well as erythrocyte volume regulation, among a multitude of other physiological functions,^18,19^ whereas Piezo2 is expressed mostly in sensory dorsal root ganglion (DRG) neurons, which are implicated in light touch sensitivity, proprioception, interoception, and nociception.^13^ Recent studies have indicated that Piezo1 in differentiated bone cells, including osteoblasts and osteocytes, contributes to bone formation.^20–24^ However, the specific role of Piezo1 in the fate decision of BMMSCs, as well as in bone and marrow adiposity remodeling upon physical exercise, remains to be explored in an in vivo setting.

Thus, we embarked on exploring the physiological role of Piezo1 in controlling the lineage commitment of BMMSCs and investigated the molecular pathways linking Piezo1 to cell fate decision. In brief, we discovered that Piezo1 activation is a major negative regulator of bone marrow adipogenesis by suppressing local inflammation, thereby contributing to the beneficial effects of physical exercise on bone strength.

## Results

### Piezo1 invalidation in PDGFRα-expressing cells causes osteoporosis and bone marrow adiposity in mice

Our initial aim was to explore the potential role of Piezo1 in the development and function of adipose tissues. To this end, we intended to use the Platelet-Derived Growth Factor Receptor Alpha (PDGFRα)-Cre mice to genetically invalidate Piezo1 in adipocyte progenitor cells.^25^ In addition to adipocyte progenitor cells, previous studies have shown that PDGFRα is also expressed in BMMSCs, brain oligodendrocyte progenitor cells (OPCs), and muscle fibro-adipogenic progenitors (FAPs).^26–28^ Therefore, we first generated PDGFRα-Cre tdTomato reporter mice to demonstrate the tissue distribution pattern of tdTomato driven by the PDGFRα promoter by crossing PDGFRα-Cre mice with tdTomato fluorescent reporter (Ai14) mice (supplementary Fig. 1a). Examination of the tdTomato fluorescence intensity across different tissues of the reporter mice revealed its most abundant enrichment in the bone marrow, followed by subcutaneous white adipose tissue (scWAT), OPC-enriched brain regions (subventricular zone [SVZ], white matter, and corpus callosum), and skeletal muscles (gastrocnemius, quadriceps, and soleus) (supplementary Fig. 1b). Consistently, fluorescence microscopy revealed that tdTomato-expressing (PDGFRα^+^) cells were most abundant in BMMSCs, moderately present in stromal vascular fractions (SVFs) and brain areas containing OPCs, scattered in skeletal muscle, but completely absent in non-OPC-containing brain area such as the hypothalamus (supplementary Fig. 1c, d).

Next, we generated mice with specific depletion of Piezo1 in PDGFRα^+^ cells (named PDGFRα-Piezo1 KO mice) by crossing Piezo1^flox/flox^ mice (WT) with PDGFRα-Cre mice. As expected, both qPCR and immunofluorescence staining confirmed the ablation of Piezo1 in SVFs and BMMSCs of PDGFRα-Piezo1 KO mice, with no compensatory expression of Piezo2 in Piezo1 KO SVFs and BMMSCs (supplementary Fig. 2a, b, d). On the other hand, there was no difference in Piezo1 expression in the brain or skeletal muscle between WT and KO mice (supplementary Fig. 2c, d), which could be explained by the undetectable Piezo1 expression in OPC-enriched brain areas and the absence of colocalization between Piezo1-expressing cells and tdTomato-expressing PDGFRα^+^ cells in skeletal muscle (supplementary Fig. 1d). Taken together, these observations indicate that the PDGFRα-Cre driver enables the selective ablation of Piezo1 in BMMSCs and SVFs.

**Fig. 1:**
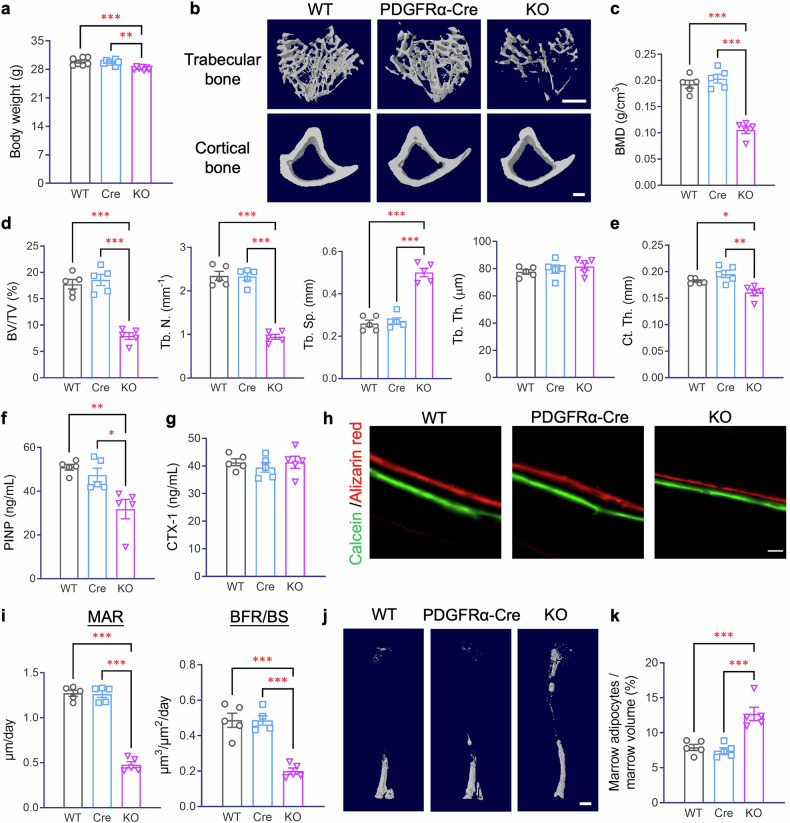
PDGFRα-Piezo1 KO mice display osteoporosis and bone marrow adiposity. Eighteen-week-old Piezo1^flox/flox^ (WT), PDGFRα-Cre (Cre) and PDGFRα-Piezo1 knockout (KO) male mice fed with a standard chow diet (STC) were sacrificed. Tibias were isolated for micro-CT analysis and osmium tetroxide staining. **a** Body weight. *n* = 7. **b** Representative images showing three-dimensional trabecular architecture and cortical bone by micro-CT reconstruction at the proximal tibia. Scale bar, 500 μm. **c** Bone mineral density (BMD) determined by micro-CT analysis. *n* = 5. **d** Micro-CT measurements of the bone volume fraction (BV/TV), trabecular number (Tb. N.), trabecular separation (Tb. Sp.), and trabecular thickness (Tb. Th.) at the proximal tibia. *n* = 5. **e** Micro-CT analysis of cortical thickness (Ct. Th.) at the proximal tibia. *n* = 5. **f**, **g** Circulating levels of PINP (**f**) and CTX-1 (**g**) measured by ELISA. *n* = 5. **h** Representative calcein and alizarin red double labeling images of tibias. Scale bar, 50 μm. **i** Quantification of the mineral apposition rate (MAR) (left panel) and the bone formation rate per unit of bone surface (BFR/BS) (right panel). **j**, **k** Tibias were fixed with 4% paraformaldehyde (PFA) for overnight and decalcified in 0.5 M EDTA (pH = 7.4) for 21 days, followed by osmium tetroxide staining and micro-CT scanning. **j** Representative micro-CT images showing osmium-stained bone marrow adipocytes (BMAs) in decalcified tibias. Scale bar, 1000 μm. **k** Quantification of BMAs in the tibia normalized to marrow volume. *n* = 5. The data are presented as the means ± SEMs; *p < 0.05, **p < 0.01, ***p < 0.001. See also supplementary Figs. S1–S6

During metabolic characterization, we found that PDGFRα-Piezo1 KO mice on both standard chow (STC) and high-fat diet (HFD) feeding showed significantly lower body weights than their WT littermates and PDGFRα-Cre controls (Fig. 1a and supplementary Fig. 3a), whereas their body length, fat and lean mass, weight and morphology of different adipose tissues and skeletal muscles, oxygen consumption, respiratory exchange ratio (RER), and secretion of adipokines by peripheral adipose tissues remained unchanged (supplementary Figs. 3b–h and 4a–e). Taken together, these data indicate that the lower body weight observed in PDGFRα-Piezo1 KO mice is unrelated to growth retardation or the effect of Piezo1 invalidation in peripheral adipocyte progenitor cells.

Notably, microcomputed tomography (micro-CT) analysis revealed a significant reduction in bone volume and bone density in PDGFRα-Piezo1 KO mice compared with WT and PDGFRα-Cre control mice, as evidenced by markedly lower trabecular bone volume and number, increased trabecular separation, and reduced thickness of cortical bone (Fig. 1b–e). However, the lengths of major long bones (femurs and tibias) were similar between WT and KO mice (supplementary Fig. 4f), suggesting that skeletal development is unaltered in PDGFRα-Piezo1 KO mice. Notably, the reduction in bone volume in PDGFRα-Piezo1 KO mice was accompanied by a significantly lower circulating level of procollagen type I N-propeptide (PINP, a bone formation marker^29^), whereas the serum level of type I collagen cross-linked C-telopeptide (CTX-1, a bone resorption marker^30^) remained unchanged (Fig. 1f, g). Histological analysis revealed that PDGFRα-Piezo1 KO mice had significantly fewer alkaline phosphatase (ALP)-stained osteoblasts, but a similar number of tartrate-resistant acid phosphatase (TRAP)-stained osteoclasts compared with their WT littermates and PDGFRα-Cre controls (supplementary Fig. 5a–d). Dynamic histomorphometry via in vivo double labeling with calcein and alizarin red^31^ showed significantly reduced bone formation in PDGFRα-Piezo1 KO mice, as evidenced by decreased bone formation rate (BFR) and mineral apposition rate (MAR) (Fig. 1h, i). Conversely, PDGFRα-Piezo1 KO mice exhibited a significantly increased volume of bone marrow adipocytes (BMAs) compared to their controls, as determined by osmium tetroxide staining of decalcified tibias followed by micro-CT analysis^32^ (Fig. 1j, k) and histological analysis via hematoxylin and eosin (H&E) staining (supplementary Fig. 5e–g). Similarly, female PDGFRα-Piezo1 KO mice also displayed reduced bone volume and increased bone marrow adiposity compared to their WT littermates (supplementary Fig. 6a–k). Since PDGFRα-Cre control mice and Piezo1^flox/flox^ WT mice displayed similar phenotypes, only Piezo1^flox/flox^ WT mice (littermates of PDGFRα-Piezo1 KO mice) were included in all subsequent experiments. Collectively, these data suggest that ablation of Piezo1 in PDGFRα^+^ cells impairs osteogenesis but increases bone marrow adiposity without apparent changes in peripheral adipose tissues.

### Piezo1-deficient BMMSCs preferentially differentiate into adipocytes rather than osteoblasts

Given the abundant expression of PDGFRα in BMMSCs (supplementary Fig. 1c),^26,33^ the decreased bone volume and increased marrow adiposity in PDGFRα-Piezo1 KO mice are likely attributed to Piezo1 deficiency in BMMSCs. To explore this possibility, we further used the cell-attached patch clamp configuration to measure Piezo1 currents in response to fast negative pressure pulses to confirm successful invalidation of Piezo1 channel activity in BMMSCs isolated from PDGFRα-Piezo1 KO mice. For electrophysiological recording, we used PDGFRα-Cre tdTomato reporter mice to identify tdTomato-positive BMMSCs in culture. The pressure-induced inward currents (holding potential of −80 mV) were characterized by an absence of inactivation and a remarkably slow deactivation when pressure stimulation was resumed. In contrast, the pressure-induced current was dramatically reduced in Piezo1 KO BMMSCs (supplementary Fig. 7a), indicating that the mechanogated ion channel recorded in mouse BMMSCs is due to Piezo1.

**Fig. 2:**
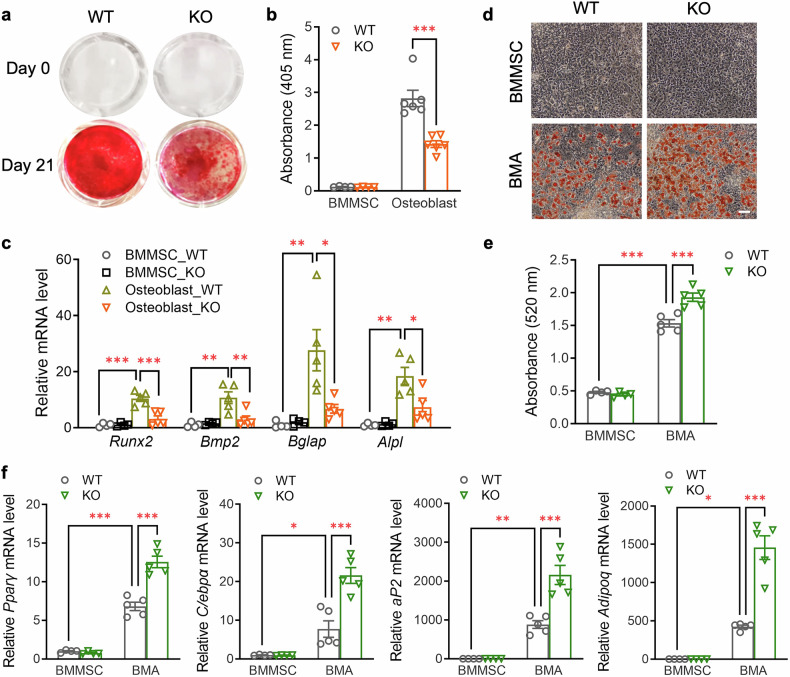
Piezo1 invalidation in BMMSCs results in reduced osteogenesis but enhanced adipogenesis. **a–f** BMMSCs isolated from femurs and tibias of 10-week-old male PDGFRα-Piezo1 KO mice and WT controls were cultured in α-MEM and differentiated into osteoblasts or BMAs by adding an osteogenic cocktail (100 nM dexamethasone, 50 μg/mL ascorbic acid, and 10 mM β-glycerophosphate) or an adipogenic cocktail (1 μM dexamethasone, 0.5 mM IBMX, 1 μM rosiglitazone, and 1.8 μM insulin), respectively. **a** Alizarin Red S staining of osteoblasts on day 0 and day 21 after differentiation. **b** Quantitative analysis of Alizarin Red S staining by determining the OD405 absorbance values. *n* = 4–6. (**c**) Real-time PCR analysis of the mRNA levels of osteogenic genes (*Runx2, Bmp2, Bglap*, and *Alpl*) on day 21 after osteogenic differentiation. *n* = 4–5. **d** Oil Red O staining of BMMSC-derived BMAs on day 8 after differentiation and their undifferentiated controls. Scale bar, 100 μm. **e** Quantitative analysis of Oil Red O staining by determining the OD520 absorbance values. *n* = 4–5. **f** Real-time PCR analysis of several adipogenic genes (*Pparγ, C/ebpα, aP2*, and *Adipoq*) after 8 days of adipogenic differentiation. *n* = 4–5. The data are presented as the means ± SEMs; *p < 0.05, **p < 0.01, ***p < 0.001. See also supplementary Figs. S7–S9

Next, we compared the osteogenic and adipogenic capacity of BMMSCs with or without Piezo1 in vitro. Consistent with our in vivo findings, Piezo1 KO BMMSCs exhibited much lower osteogenic capacity compared to WT BMMSCs, as determined by Alizarin Red S staining (Fig. 2a, b) and real-time PCR analysis of the mRNA expression of four osteoblast-related genes (*Runx2*, bone morphogenetic protein 2 [*Bmp2*], osteocalcin [*Bglap*], and alkaline phosphatase [*Alpl*]) (Fig. 2c). In contrast, Piezo1 deficiency in BMMSCs markedly increased adipogenic capacity, as evidenced by significant increases in Oil Red O-stained adipocytes and significantly higher expression of adipocyte-related genes (*Pparγ*, *C/ebpα*, adipocyte protein 2 [*aP2*], and adiponectin [*Adipoq*]) (Fig. 2d–f), suggesting that ablation of Piezo1 shifts the lineage commitment of BMMSCs from osteoblasts towards adipocytes. Notably, Piezo1 KO BMMSCs and WT controls exhibited comparable expression levels of genes related to multipotency (*Sox2*, *Oct4*, and *Nanog*), senescence (*p16*^*Ink4a*^ and *p21*^*Cip1*^), and proliferation (*Ki67*), as well as similar enzymatic activity of senescence-associated β-galactosidase (SA-β-gal) (supplementary Fig. 7b–d). Contrary to BMMSCs, SVFs isolated from the scWAT of PDGFRα-Piezo1 KO mice and WT littermates exhibited comparable mRNA levels of adipogenic genes and similar amounts of Oil Red O-stained adipocytes after the induction of differentiation, irrespective of the presence or absence of rosiglitazone (supplementary Fig. 8a–d). These findings indicate that Piezo1 invalidation in PDGFRα^+^ cells has a specific effect on the balance between the differentiation of osteoblasts and BMAs in BMMSCs.

Considering the significant role of Piezo1 in mouse BMMSCs, we further investigated whether Piezo1 plays a similar role in human BMMSCs (hBMMSCs) by treating hBMMSCs with the Piezo1 activator Yoda1 (5 μM) or the Piezo1 inhibitor GsMTx4 (5 μM). We found that Piezo1 activation by Yoda1 significantly suppressed adipogenesis but promoted osteogenesis. Conversely, inhibition of Piezo1 via GsMTx4 increased adipogenesis but inhibited osteogenesis, as evidenced by qPCR analysis of adipogenic and osteogenic genes, together with Oil Red O staining of BMAs and Alizarin Red S staining of osteoblasts (supplementary Fig. 9a–d).

### The reduced bone volume and increased marrow adiposity in PDGFRα-Piezo1 KO mice are exclusively due to Piezo1 depletion in BMMSCs

**Fig. 3:**
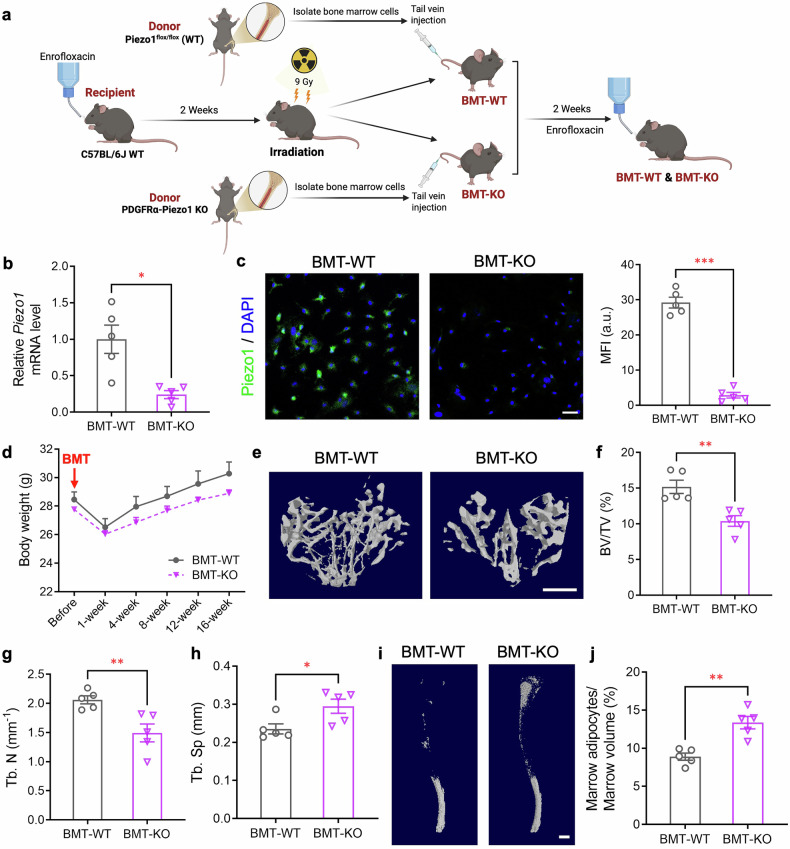
Transplantation of Piezo1-deficient bone marrow cells into WT mice causes osteoporosis and bone marrow adiposity. Twelve-week-old C57BL/6J recipient mice were subjected to 9 Gy total body irradiation to deplete the bone marrow. In the following day, bone marrow cells were isolated from femurs and tibias of 6-week-old male donor mice (PDGFRα-Piezo1 KO mice and their WT littermates). A total of 1 × 10^7^ donor bone marrow cells were intravenously injected into a recipient mouse via the tail vein. The recipient mice were treated with enrofloxacin (0.17 mg/mL) in the drinking water for 2 weeks before irradiation and after bone marrow transplantation (BMT). **a** Schematic diagram showing the experimental design created with BioRender (https://BioRender.com). **b** The mRNA level of *Piezo1* in BMMSCs isolated from mice after BMT. *n* = 5. **c** Immunofluorescence staining of Piezo1 expression (green) in BMMSCs from recipient mice after BMT for 10 weeks (left panel). Nuclei were stained with DAPI (blue). Scale bar, 50 μm. The right panel shows the quantification of the mean fluorescence intensity (MFI) of Piezo1. a.u., arbitrary unit. **d** Changes in body weight. *n* = 5. **e** Representative images showing three-dimensional trabecular bone reconstructed via micro-CT at the proximal tibia. Scale bar, 500 μm. **f**–**h** Micro-CT analyses of the bone volume fraction (**f**), trabecular number (**g**), and trabecular separation (**h**) at the proximal tibia. *n* = 5. **i** Representative micro-CT images showing decalcified tibias stained with osmium tetroxide. Scale bar, 1000 μm. **j** Percentage of BMAs in the tibia determined by osmium tetroxide staining, followed by micro-CT analysis. *n* = 5. The data are presented as the means ± SEMs. *p < 0.05, **p < 0.01, ***p < 0.001. See also supplementary Fig. S10

To further confirm that the altered bone volume and BMAs in PDGFRα-Piezo1 KO mice are specifically attributed to Piezo1 deficiency in BMMSCs, we next investigated whether the transplantation of Piezo1-deficient bone marrow cells into irradiated C57BL/6J WT mice is sufficient to recapitulate the phenotypes observed in PDGFRα-Piezo1 KO mice (Fig. 3a). We first used our PDGFRα-Cre tdTomato reporter mice as donor mice to confirm that BMMSCs could be successfully engrafted after bone marrow transplantation (BMT). In vivo imaging system (IVIS) revealed obvious tdTomato fluorescence in the femurs and tibias of WT mice after transplantation with the bone marrow from PDGFRα-Cre tdTomato reporter mice (abbreviated as BMT-tdTomato) for 10 weeks (supplementary Fig. 10a). Additionally, the tdTomato signal could also be visualized in BMMSCs isolated from BMT-tdTomato mice (supplementary Fig. 10b), confirming the successful engraftment of BMMSCs after BMT. Next, we conducted real-time PCR analysis and immunofluorescence staining to confirm the successful reconstitution of Piezo1-deficient BMMSCs in WT recipient mice (Fig. 3b, c). Notably, the body weights of the recipient mice decreased at the first week after BMT and then gradually recovered (Fig. 3d). Mice transplanted with Piezo1 KO bone marrow (BMT-KO) began to show a decreasing trend in body weight at 4 weeks after bone marrow transplantation compared to those transplanted with WT bone marrow (BMT-WT) (Fig. 3d), whereas the fat percentage and oxygen consumption did not differ between these two groups of mice (supplementary Fig. 10c, d). On the other hand, micro-CT and osmium tetroxide staining analyses showed that mice transplanted with Piezo1-deficient bone marrow cells displayed significantly reduced bone volume and trabecular number but increased trabecular separation (Fig. 3e–h), while the amount of BMAs was significantly increased compared to those transplanted with WT bone marrow cells (Fig. 3i, j). Collectively, these data indicate that specific Piezo1 invalidation in BMMSCs alone is sufficient to increase bone marrow adiposity and reduce bone volume.

### Exercise-induced bone formation and loss of BMAs are abolished in PDGFRα-Piezo1 KO mice

**Fig. 4:**
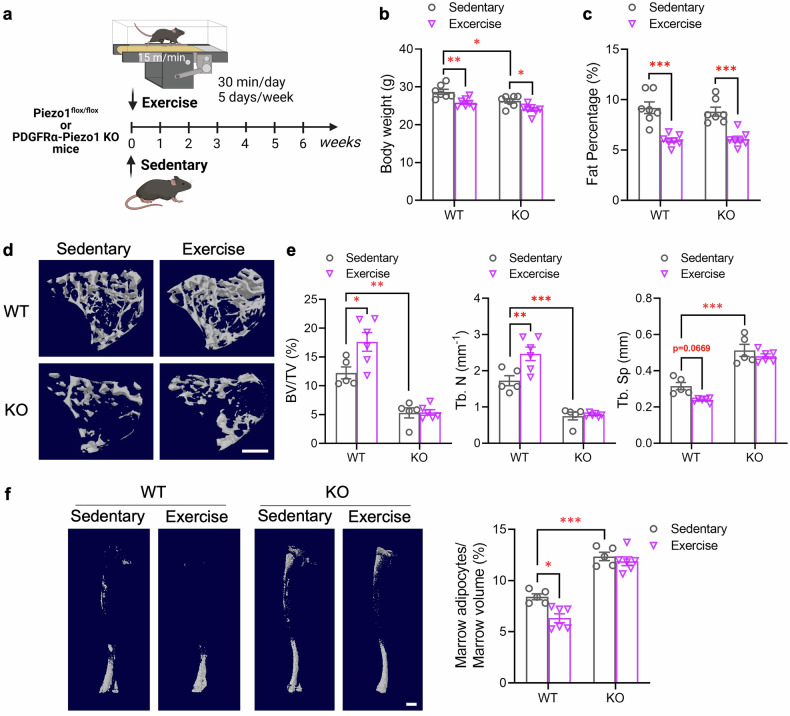
Loss of Piezo1 dampens exercise-induced bone formation and reduction of BMAs. Eight-week-old male PDGFRα-Piezo1 KO mice and their WT littermates were subjected to treadmill exercise for 6 weeks (5 days per week, 30 min per day, at a speed of 15 m/min). Another two age-matched groups without exercise were used as sedentary controls. **a** Schematic diagram showing the experimental design created with BioRender (https://BioRender.com). **b**, **c** Body weight (**b**) and fat percentage (**c**) at 14 weeks of age. *n* = 7. **d** Representative images of micro-CT analysis showing three-dimensional trabecular bone at the proximal tibia. Scale bar, 500 μm. **e** Micro-CT measurements of the bone volume fraction (BV/TV), trabecular number (Tb. N.), and trabecular separation (Tb. Sp.) at the proximal tibia. *n* = 5–6. **f** Representative images showing osmium-stained decalcified tibias scanned via micro-CT. Scale bar, 1000 μm. The right panel shows the quantification of bone marrow adipocytes in the tibia normalized to the marrow volume. *n* = 5–6. The data are presented as the means ± SEMs; *p < 0.05, **p < 0.01, ***p < 0.001. See also supplementary Fig. S11

Physical exercise has beneficial effects on bone health^34^ and suppresses the accumulation of BMAs,^35^ at least in part by enhancing the osteogenic potential and inhibiting the adipogenic capacity of BMMSCs.^36^ Given that Piezo1 is a mechanosensitive ion channel, we next investigated whether Piezo1 in BMMSCs can sense the mechanical loading generated by treadmill exercise to mediate the effects of exercise on the conversion of BMMSCs into osteoblasts and adipocytes (Fig. 4a). Both PDGFRα-Piezo1 KO mice and their WT littermates after 6-week treadmill exercise displayed significant reductions in body weight and peripheral fat content (Fig. 4b, c). However, exercise significantly increased bone volume and trabecular number but decreased trabecular separation in WT mice, while this effect was largely abrogated in PDGFRα-Piezo1 KO mice (Fig. 4d, e). Osmium tetroxide staining revealed that exercise markedly reduced the number of BMAs in WT mice, whereas this reduction was blunted in PDGFRα-Piezo1 KO mice (Fig. 4f). On the other hand, exercise induced comparable increases in the weight of different types of skeletal muscles (quadriceps and gastrocnemius) and muscle fiber hypertrophy, as well as similar changes in the expression of several genes related to muscle development/metabolism between PDGFRα-Piezo1 KO mice and WT controls (supplementary Fig. 11a–c), suggesting that the blunted effects of exercise on bone formation and reduction in bone marrow adiposity are unrelated to changes in skeletal muscles.

### Lipocalin-2 (Lcn2) acts as a key downstream effector of Piezo1 in regulating the conversion of BMMSCs into adipocytes

**Fig. 5:**
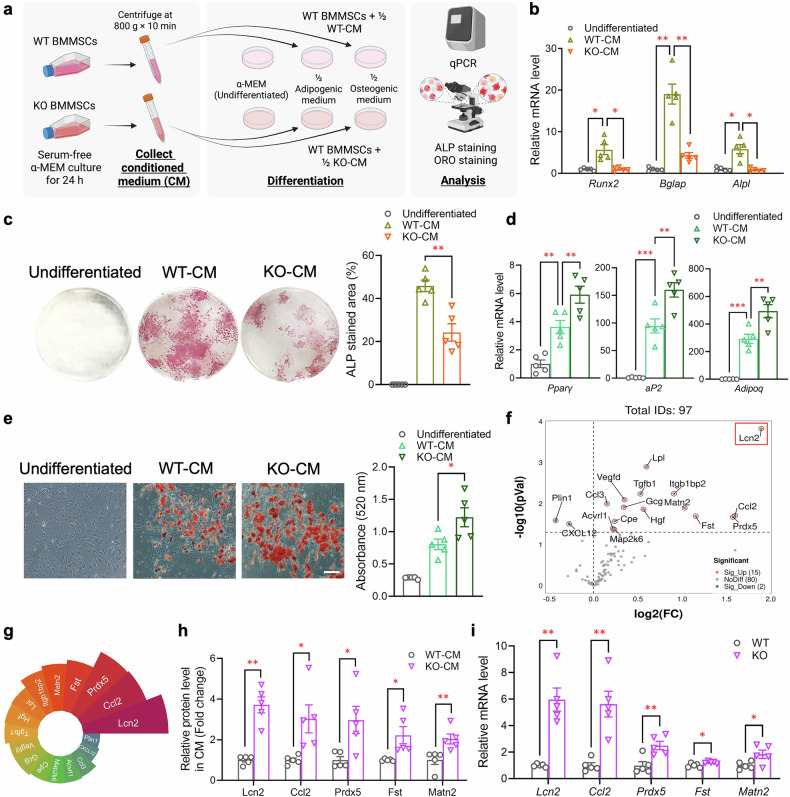
Piezo1 modulates the lineage commitment of BMMSCs in an autocrine manner. BMMSCs were isolated from femurs and tibias of 10-week-old male C57BL/6J mice and then cultured in medium containing half of the conditioned medium (CM) collected from 10-week-old male WT or KO BMMSCs and half of the osteogenic or adipogenic induction medium. **a** Schematic diagram showing the experimental design created with BioRender (https://BioRender.com). **b** qPCR analysis of osteogenic genes (*Runx2, Bmp2, Bglap*, and *Alpl*). **c** Representative images of alkaline phosphatase (ALP) staining. The right panel shows the quantification of the ALP-stained area via ImageJ software. **d** qPCR analysis of adipogenic genes (*Pparγ, C/ebpα, aP2*, and *Adipoq*). **e** Representative images of Oil Red O staining. The right panel shows the quantitative analysis of Oil Red O staining by determining the OD520 absorbance values. Scale bar, 100 μm. **f** Conditioned media collected from WT and KO BMMSCs were subjected to analyses of secretory factors via an Olink Mouse Exploratory Panel, multiplex assay (Mouse Luminex® Discovery Assay, R&D Systems), and ELISA. Volcano plot showing the log2 fold change (FC) and p value (pVal) of differentially secreted proteins between Piezo1 KO and WT BMMSCs among the 97 soluble factors analyzed (log2(FC) > 1, −log10(p)<0.05). **g** The Nightingale rose chart displays the ranking of fold changes in the expression of 17 differentially secreted proteins between Piezo1 KO and WT BMMSCs. **h** Relative protein levels in the conditioned medium of WT and KO BMMSCs are presented as the fold change. **i** qPCR analysis of *Lcn2*, *Ccl2*, *Prdx5*, *Fst*, and *Matn2* in BMMSCs. *n* = 5 for each group. The data are presented as the means ± SEMs; *p < 0.05, **p < 0.01, ***p < 0.001. See also supplementary Fig. S12 and Table S1

Cumulative evidence has demonstrated the importance of BMMSC-derived autocrine factors in determining the lineage commitment of BMMSCs.^37–41^ To investigate whether Piezo1 modulates the autocrine/paracrine effects of BMMSCs to control lineage commitment, we treated WT BMMSCs with conditioned medium (CM) collected from cultured WT and Piezo1 KO BMMSCs (Fig. 5a). Our results revealed that BMMSCs treated with conditioned medium from Piezo1 KO BMMSCs significantly dampened osteogenesis but promoted adipogenesis, as evidenced by the reduced expression of osteogenic genes (Fig. 5b), decreased alkaline phosphatase (ALP)-stained osteoblasts (Fig. 5c), upregulated expression of adipogenic genes (Fig. 5d) and increased Oil Red O-stained BMAs (Fig. 5e).

To identify secretory factors involved in the downstream effects of Piezo1 in modulating osteogenesis or adipogenesis of BMMSCs, we performed Olink-based proteomics for the quantitative analysis of 92 soluble factors in the conditioned media of Piezo1 KO and WT BMMSCs. Additionally, we also used multiplex assay and enzyme-linked immunosorbent assay (ELISA) to measure a panel of cytokines, chemokines, and growth factors, including C-C motif chemokine ligand 7 (Ccl7), C-X-C motif chemokine ligand 12 (Cxcl12), insulin-like growth factor 1 (Igf-1), fatty acid binding protein 4 (Fabp4) and Lcn2, which have been implicated in adipogenesis and/or bone homeostasis^42–46^ but are not included in the Olink Mouse Exploratory panel. Among the 97 soluble factors analyzed, we identified 17 differentially secreted proteins (15 upregulated and 2 downregulated) between Piezo1 KO and WT BMMSCs (Fig. 5f, supplementary Fig. 12a–f, and supplementary Table 1). The top five significantly altered factors with a fold change >2 were Lcn2, C-C motif chemokine ligand 2 (Ccl2), peroxiredoxin 5 (Prdx5), follistatin (Fst), and matrilin 2 (Matn2) (Fig. 5g, h). Consistent with the changes in protein levels in the conditioned medium, the mRNA abundance of these five genes was also significantly increased in Piezo1 KO BMMSCs, with both *Lcn2* and *Ccl2* showing greater increases in KO cells (Fig. 5i). Similarly, a modest but significant elevation in the serum Lcn2 and Ccl2 levels was also observed in Piezo1 KO mice (supplementary Fig. 12g, h).

**Fig. 6:**
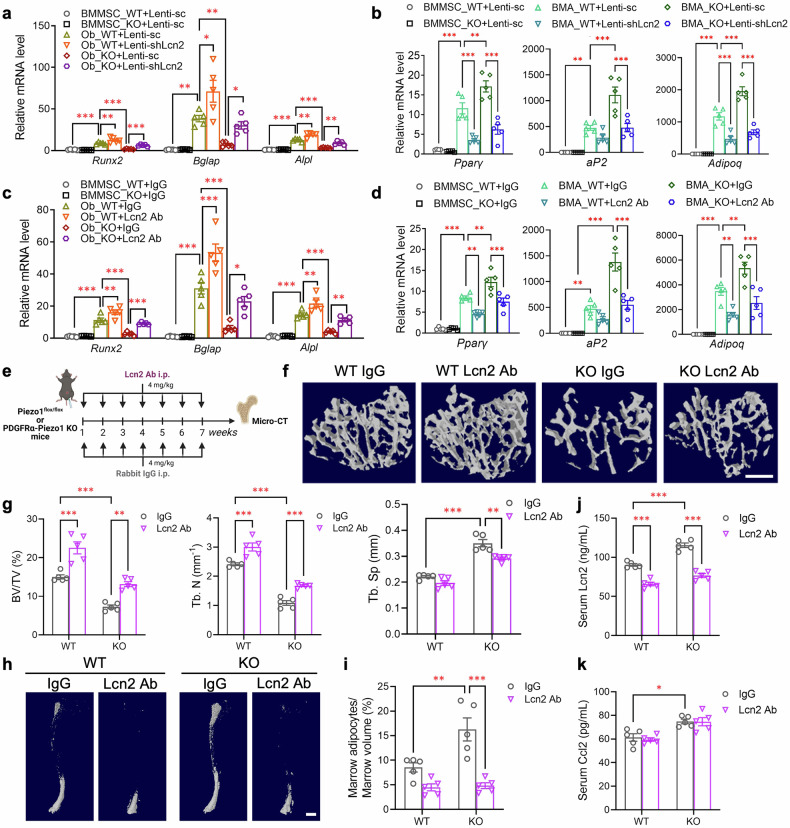
Genetic inhibition or antibody neutralization of Lcn2 could reverse the reduced osteogenesis and enhanced adipogenesis caused by Piezo1 invalidation in BMMSCs. **a**, **b** BMMSCs isolated from femurs and tibias of 10-week-old male PDGFRα-Piezo1 KO mice and their WT littermates were infected with lentivirus (multiplicity of infection [MOI] = 40) encoding *eGFP* together with scrambled shRNA (Lenti-sc) or shRNA against *Lcn2* (Lenti-shLcn2) for 72 h in the presence of 5 μg/mL polybrene before differentiation. Osteogenic (**a**) and adipogenic (**b**) gene expression was determined by qPCR after 21 days of osteogenic differentiation and 8 days of adipogenic differentiation, respectively. **c**, **d** 200 ng/mL rabbit anti-mouse Lcn2 neutralizing antibody or non-immune rabbit IgG was added when the differentiation of BMMSCs started. qPCR analysis of osteogenic (**c**) and adipogenic genes (**d**). **e**–**k** Eight-week-old WT and KO mice were intraperitoneally injected with non-immune rabbit IgG or Lcn2 neutralizing antibody (4 mg/kg body weight) on a weekly basis for 7 weeks. The mice were then sacrificed, and their tibias were collected for further analyses. **e** Schematic diagram showing the experimental design created with BioRender (https://BioRender.com). **f** Representative micro-CT images showing three-dimensional trabecular bone at the proximal tibia. Scale bar, 500 μm. **g** Micro-CT measurements of the bone volume fraction (BV/TV), trabecular number (Tb. N), and trabecular separation (Tb. Sp) at the proximal tibia. **h** Representative images showing osmium-stained decalcified tibias scanned via micro-CT. Scale bar, 1000 μm. **i** Quantification of bone marrow adipocytes in the tibia normalized to marrow volume. **j**, **k** Circulating levels of Lcn2 (**j**) and Ccl2 (**k**) determined by ELISA. *n* = 5 for each group. The data are presented as the means ± SEMs; *p < 0.05, **p < 0.01, ***p < 0.001. See also supplementary Figs. S13–S14 and Table S2

Notably, the top-ranked candidate Lcn2 (approximately 6-fold increase in mRNA abundance and 3.7-fold increase in protein secretion in Piezo1 KO BMMSCs) was previously identified as a mechanoresponsive gene in different tissues^45^ and was predicted as a potential Piezo1-associated gene in bone-related diseases by a recent bioinformatic study.^47^ Therefore, we next investigated whether Lcn2 may act as a downstream effector of Piezo1 in the modulation of BMMSC differentiation in an autocrine manner. Lentivirus-mediated knockdown of Lcn2 abolished Piezo1 deficiency-induced Lcn2 mRNA expression and protein secretion (supplementary Fig. 13a, b) and markedly reversed the decreased osteogenesis and increased adipogenesis of Piezo1 KO BMMSCs (Fig. 6a, b and supplementary Fig. 14a, b). Similarly, the direct addition of an anti-Lcn2 antibody, but not non-immune IgG, into the culture medium was sufficient to reverse the reduced osteogenesis and increased adipogenesis in Piezo1 KO BMMSCs (Fig. 6c, d and supplementary Fig. 14c, d). Importantly, these in vitro observations were further validated by chronic, intraperitoneal injection of a Lcn2 neutralizing antibody into PDGFRα-Piezo1 KO mice, as shown by the recovery of decreased bone volume and trabecular number, together with the reversal of enhanced trabecular separation and increased bone marrow adiposity (Fig. 6e–i). Notably, treatment with the anti-Lcn2 antibody significantly decreased circulating Lcn2 levels in both WT and PDGFRα-Piezo1 KO mice (Fig. 6j), possibly due to the neutralizing antibody-induced epitope masking disrupted Lcn2 detection via ELISA. On the other hand, antibody neutralization of Lcn2 did not alter the circulating concentration of Ccl2 in either WT or KO mice (Fig. 6k).

### Piezo1 invalidation potentiates Ccl2-CCR2 signaling to promote Lcn2 expression and secretion

**Fig. 7:**
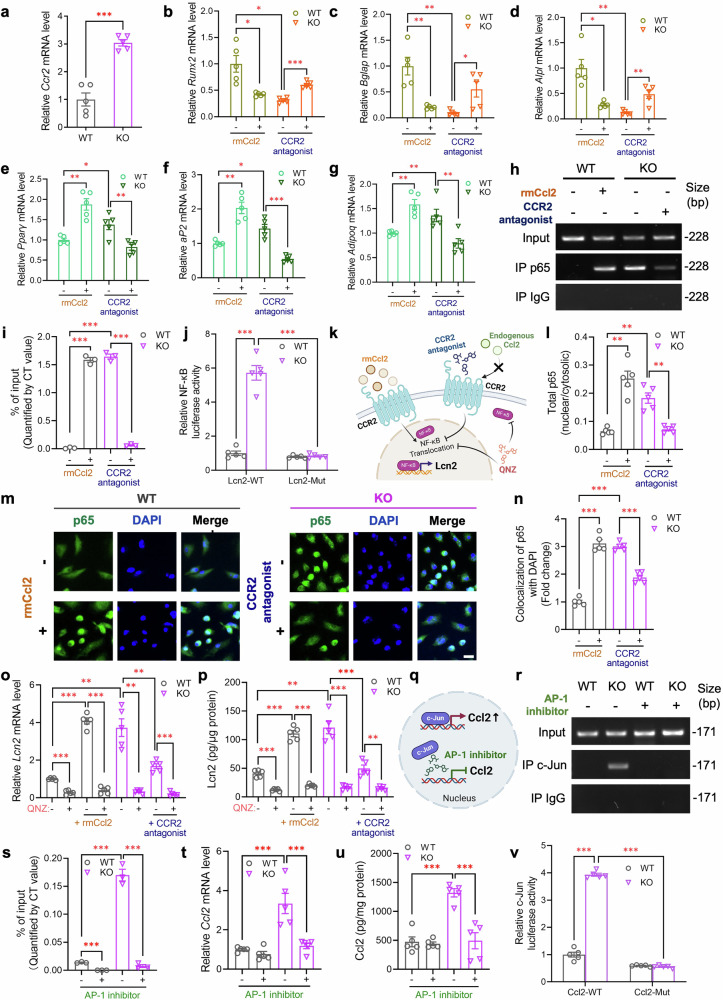
Piezo1 suppresses Lcn2 expression and secretion via the inhibition of Ccl2-evoked NF-κB activation. BMMSCs were isolated from femurs and tibias of 10-week-old male PDGFRα-Piezo1 KO mice and WT controls. **a** Gene expression of *Ccr2* in BMMSCs, as determined by real-time PCR. **b–g** Recombinant mouse Ccl2 protein (rmCcl2, 100 ng/mL) or the CCR2 antagonist INCB3344 (10 nM) was added to the adipogenic and osteogenic induction medium during BMMSC differentiation. The expression levels of osteogenic genes (**b–d**) and adipogenic genes (**e–g**) were determined via real-time qPCR. **h–n** WT and KO BMMSCs were treated with rmCcl2 (100 ng/mL) or the CCR2 antagonist INCB3344 (10 nM) for 24 h. **h** Sonicated BMMSCs were subjected to chromatin immunoprecipitation (ChIP) using an anti-p65 antibody. The ChIP DNA samples were then subjected to qPCR amplification using specific primers against the promoter region of Lcn2, as shown in supplementary Fig. 15b. Representative gel images of end-point ChIP-qPCR products are shown. **i** Quantitative results are shown as the percentage of input DNA: % of Input = 2^(CT^Input^−CT^IP^)/dilution factor × 100%. **j** Luciferase reporter assay was performed in isolated WT and KO BMMSCs transfected with pGL3 constructs containing the mouse wild-type Lcn2 promoter (Lcn2-WT) or the mutated Lcn2 promoter with mutations in the NF-κB binding motif (Lcn2-Mut, sequence in supplementary Fig. 15c). The firefly luciferase activity was measured and normalized to Renilla luciferase activity. **k** Schematic diagram illustrating the actions of rmCcl2, the CCR2 antagonist, and the NF-κB-specific inhibitor QNZ on NF-κB translocation and Lcn2 expression; created with BioRender (https://BioRender.com). **l** ELISA analysis of total p65 in the nucleus and cytoplasm of BMMSCs. **m** Representative immunofluorescence images of intracellular p65 localization. Nuclei were stained with DAPI (blue). Scale bar, 50 μm. **n** P65 nuclear translocation was quantified by ImageJ software via Pearson’s correlation coefficient (PCC) between the p65 immunofluorescence signal and DAPI staining across segmented nuclear regions. **o**, **p** WT and KO BMMSCs were treated with the NF-κB-specific inhibitor QNZ (10 nM) for another 2 h after 24-h treatment of rmCcl2 (100 ng/mL) and INCB3344 (10 nM). **o** The mRNA level of *Lcn2*. **p** Lcn2 levels in the culture medium were determined by ELISA. **q** Illustration of how the AP-1 inhibitor modulates *Ccl2* gene expression; created with BioRender (https://BioRender.com). **r–u** WT and KO BMMSCs were treated with the AP-1-specific inhibitor T-5224 (10 μM) for 24 h. **r** Sonicated BMMSCs were subjected to ChIP assay using an anti-c-Jun antibody. Representative gel images of end-point ChIP-qPCR products are shown. **s** Quantitative results are shown as the percentage of input DNA. **t** The mRNA level of *Ccl2*. **u** Ccl2 levels in the culture medium were determined by ELISA. **v** Luciferase reporter assay was performed in isolated WT and KO BMMSCs transfected with pGL3 constructs containing the mouse wild-type Ccl2 promoter (Ccl2-WT) or the mutated Ccl2 promoter with mutations in the c-Jun binding motif (Ccl2-Mut, sequence in supplementary Fig. 16d). The firefly luciferase activity was measured and normalized to Renilla luciferase activity. *n* = 5 for each group. The data are presented as the means ± SEMs; *p < 0.05, **p < 0.01, ***p < 0.001. See also supplementary Figs. S14–S17

Next, we explored how Piezo1 deficiency upregulates Lcn2 expression and secretion in BMMSCs. Our qPCR analysis and Olink data revealed that both Ccl2 expression and secretion levels were greatly elevated in Piezo1 KO BMMSCs (Fig. 5g–i), and protein-protein interaction (PPI) network analysis indicated a potential interaction between Ccl2 and Lcn2 (supplementary Fig. 12i). Interestingly, Ccl2 has previously been shown to induce Lcn2 in Snail (+) tumor cells through its autocrine actions.^48^ To investigate whether Lcn2 levels are regulated through a Piezo1-dependent autocrine effect of Ccl2, we first confirmed the expression of the Ccl2 receptor CCR2 in BMMSCs and found that it was significantly upregulated in KO BMMSCs (Fig. 7a). Notably, treatment with recombinant mouse Ccl2 protein (rmCcl2) significantly shifted the differentiation potential of BMMSCs from osteogenic to adipogenic (Fig. 7b–g and supplementary Fig. 14e, f) and increased Lcn2 expression and secretion in WT BMMSCs (Fig. 7o, p). In contrast, pharmacological inhibition of Ccl2-CCR2 signaling with the CCR2 antagonist INCB3344 reversed the increased Lcn2 level in Piezo1 KO BMMSCs (Fig. 7o, p), as well as Piezo1 deficiency-induced inhibition of osteogenesis and augmentation of adipogenesis (Fig. 7b–g and supplementary Fig. 14e, f).

To address how Ccl2-CCR2 signaling modulates Lcn2 levels, we first used in silico analysis to predict the transcription factors that regulate *Lcn2* gene expression by taking advantage of four different databases, including hTFtarget, GTRD, ChIP_Atlas, and TRRUST (supplementary Fig. 15a). Nuclear factor kappa B (NF-κB) was identified as the only target in the intersection among the four databases, suggesting its possible involvement in regulating Lcn2 expression. Next, we confirmed the NF-κB binding site within the Lcn2 promoter region between −261 and −252 (supplementary Fig. 15b). DNA sequence alignment analysis demonstrated that the NF-κB binding motif was highly conserved among several mammalian species examined (supplementary Fig. 15c). Chromatin immunoprecipitation (ChIP) was subsequently performed, and the binding of p65 to the Lcn2 promoter region was validated (Fig. 7h, i). We also conducted a promoter activity assay by co-transfecting BMMSCs with pGL3-based luciferase reporters containing either the WT Lcn2 promoter (Lcn2-WT) or the mutated Lcn2 promoter with mutations in the NF-κB binding motif (Lcn2-Mut; supplementary Fig. 15c), together with a Renilla luciferase plasmid for normalization. This analysis revealed that Lcn2 promoter activity in KO BMMSCs was robustly enhanced compared with that in WT BMMSCs, whereas it was significantly attenuated when the NF-κB binding motif was mutated (Fig. 7j).

Given that activation of the Ccl2-CCR2 axis has been shown to stimulate the NF-κB signaling pathway,^49,50^ we therefore investigated whether activation or inhibition of Ccl2-CCR2 signaling controls Lcn2 expression by modulating NF-κB activity (Fig. 7k). We found that WT BMMSCs treated with rmCcl2 could markedly enhance the binding of p65 to the Lcn2 promoter region, while KO BMMSCs treated with the CCR2 antagonist INCB3344 significantly reversed the increase in p65 binding to the Lcn2 promoter (Fig. 7h, i). ELISA analysis for cytosolic and nuclear p65 and immunofluorescence staining of p65 collectively revealed that p65 translocation from the cytosol to the nucleus was significantly increased in KO BMMSCs, indicating enhanced NF-κB activity in KO BMMSCs (Fig. 7l–n). Notably, treatment with rmCcl2 potently induced the nuclear translocation of p65 in WT BMMSCs, whereas the pharmacological inhibition of CCR2 reversed the augmented nuclear translocation of p65 in KO BMMSCs (Fig. 7l-n). Additionally, treatment with the NF-κB inhibitor QNZ markedly suppressed the increase in Lcn2 gene expression and secretion in Piezo1 KO BMMSCs and WT BMMSCs treated with rmCcl2 (Fig. 7o, p).

To decipher how Piezo1 regulates Ccl2 expression, we also used the aforementioned in silico analysis to predict the potential transcription factors implicated in regulating *Ccl2* gene expression (supplementary Fig. 16a) and then validated these predictions by ChIP assay. Among the five predicted transcription factors, namely, jun proto-oncogene (c-Jun), NF-κB, Spi-1 proto-oncogene (SPI1), signal transducer and activator of transcription 1 (STAT1), and signal transducer and activator of transcription 3 (STAT3), only the binding of c-Jun with the Ccl2 promoter region was significantly enhanced in Piezo1 KO BMMSCs (supplementary Fig. 16b, c and Fig. 7r, s). DNA sequence alignment analysis revealed a highly conserved c-Jun binding motif within the Ccl2 promoter region among different mammalian species (supplementary Fig. 16d). Given that c-Jun is a key component of the transcription factor activator protein-1 (AP-1) complex,^51^ we used the AP-1 inhibitor T-5224 to further confirm that Piezo1 regulates Ccl2 expression through c-Jun^52^ (Fig. 7q). This analysis revealed that the increase in c-Jun binding to the Ccl2 promoter region, as well as the augmented Ccl2 expression and secretion in Piezo1 KO BMMSCs, were largely reversed by preincubation with the AP-1 inhibitor (Fig. 7r–u). Additionally, a promoter activity assay using luciferase reporters containing the WT Ccl2 promoter (Ccl2-WT) or mutated Ccl2 promoter with mutations in the c-Jun binding motif (Ccl2-Mut; supplementary Fig. 16d) revealed significantly enhanced Ccl2 promoter activity in KO BMMSCs than in WT BMMSCs, whereas this augmented Ccl2 promoter activity in KO BMMSCs was markedly abrogated by mutations in the c-Jun binding motif (Fig. 7v). These data collectively suggest that Piezo1 suppresses Ccl2 gene expression via c-Jun.

Interestingly, in epithelial and endothelial cells, Piezo1 activation was previously reported to induce the transcription factor Kruppel-like factor 2 (Klf2).^53,54^ Moreover, Klf2 was shown to inhibit the transcriptional activity of both NF-κB and AP-1, thus reducing the secretion of proinflammatory cytokines such as Ccl2.^53,55^ We therefore investigated whether Piezo1 may inhibit the Ccl2/Lcn2 pathway through Klf2, possibly by competing with NF-κB and AP-1. Notably, we observed significantly lower expression of *Klf2* in Piezo1 KO BMMSCs than in WT BMMSCs (supplementary Fig. 17a). Next, we performed lentivirus-mediated overexpression of Klf2 (supplementary Fig. 17a) and observed that Klf2 overexpression reversed enhanced adipogenesis and reduced osteogenesis in KO BMMSCs (supplementary Fig. 17b–e). These findings indicate that Klf2 acts as a key downstream mediator of Piezo1 in BMMSCs, influencing their fate determination. Finally, we explored whether Piezo1 opening influences Klf2 expression through the calcium/calmodulin-dependent protein kinase II (CaMKII) pathway.^54^ We observed that WT BMMSCs treated with the CaMKII inhibitor KN-93 showed significantly lower *Klf2* levels (supplementary Fig. 17f). While the Piezo1 activator Yoda1 dramatically upregulated *Klf2* expression in WT BMMSCs, this effect was markedly reversed by treatment with KN-93 (supplementary Fig. 17f), thus further highlighting the contribution of CaMKII downstream of Piezo1, which influences Klf2 expression and consequently downregulates Ccl2 and Lcn2 in BMMSCs.

### In vivo overexpression of Klf2 or knockdown of Ccl2/Lcn2 reverses the changes in bone volume and marrow adiposity in PDGFRα-Piezo1 KO mice

To validate the Klf2-Ccl2-Lcn2 axis as a downstream signaling effector of Piezo1 in modulating BMMSC fate in vivo, we performed site-specific genetic manipulation of this signaling axis via intra-tibial injection of lentiviruses encoding eGFP together with scrambled shRNA (Control), shRNA against *Ccl2* (shCcl2) or *Lcn2* (shLcn2), or overexpression of the mouse *Klf2* gene (oe Klf2) under the control of the validated PDGFRα promoter sequence (−2000/+100).^56^ We first confirmed in vitro that lentivirus-mediated Klf2 overexpression or knockdown of Ccl2 or Lcn2 in KO BMMSCs restored their expression to levels comparable to those in WT controls (supplementary Fig. 18a-b). Furthermore, intra-tibial lentiviral delivery achieved highly efficient and specific genetic manipulation of these genes in PDGFRα^+^ BMMSCs in the bone marrow (supplementary Fig. 18c–i) without significant off-target effects in peripheral adipose SVFs (supplementary Fig. 18j), confirming the efficiency and site-specificity of this strategy.

**Fig. 8:**
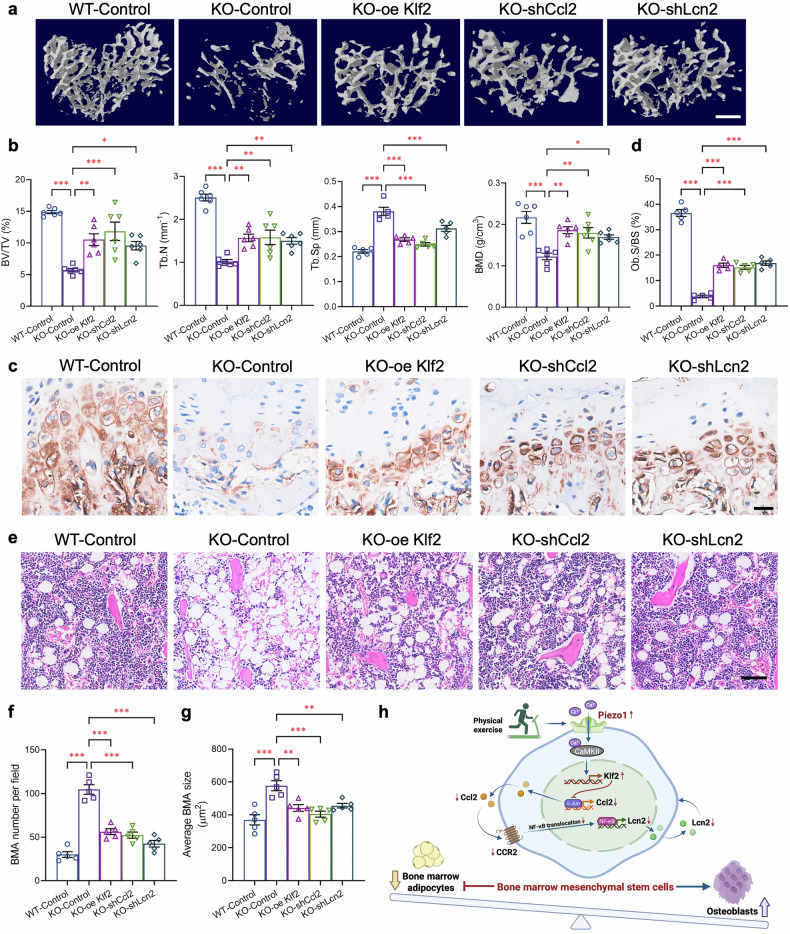
In vivo validation of the Piezo1-Klf2-Ccl2-Lcn2 signaling axis in the regulation of osteogenesis and adipogenesis in BMMSCs. Eight-week-old male PDGFRα-Piezo1 KO mice and their WT littermates were subjected to bilateral tibial injection of lentivirus encoding *eGFP* together with scrambled shRNA (WT-Control or KO-Control), shRNA against *Ccl2* (KO-shCcl2) or *Lcn2* (KO-shLcn2), or overexpression of the mouse *Klf2* gene (KO-oe Klf2) driven by the *PDGFRα* promoter at a dose of 1.25 × 10^6^ TU per side for 7 weeks. **a** Representative micro-CT images showing three-dimensional trabecular bone at the proximal tibia. Scale bar, 500 μm. **b** Micro-CT analysis of the bone volume fraction (BV/TV), trabecular number (Tb. N.), trabecular separation (Tb. Sp.), and bone mineral density (BMD) at the proximal tibia. **c**, **d** Representative images of immunohistochemical staining of ALP in paraffin-embedded decalcified tibias (**c**) and quantification of osteoblast surface as a percentage of bone surface (Ob.S/BS, %) (**d**). Scale bar, 25 μm. **e** Representative images showing H&E staining of paraffin-embedded decalcified tibias. Scale bar, 50 μm. **f**, **g** Quantification of the BMA number per field (**f**) and average BMA size (μm^2^) (**g**). **h** A graphical diagram summarizing the key findings in this study created with BioRender (https://BioRender.com). *n* = 5 for each group. The data are presented as the means ± SEMs; *p < 0.05, **p < 0.01, ***p < 0.001. See also supplementary Fig. S18 and Table S2

Consistent with our in vitro findings, restoring Klf2 expression in PDGFRα^+^ BMMSCs reversed the reduced bone volume, decreased trabecular number, lower bone mineral density, decreased ALP-stained osteoblasts, elevated trabecular separation, and increased BMAs in Piezo1 KO mice (Fig. 8a–g). Similarly, Ccl2 or Lcn2 knockdown in Piezo1-deficient PDGFRα^+^ BMMSCs to a level comparable to that in WT BMMSCs was sufficient to alleviate bone loss and marrow adiposity in Piezo1 KO mice (Fig. 8a–g). Furthermore, in vivo Ccl2 knockdown reduced Lcn2 expression and secretion, while Klf2 overexpression suppressed both Ccl2 and Lcn2 expression and secretion in KO BMMSCs (supplementary Fig. 18f, g). These series of data with sequential modulation of Klf2, Ccl2, and Lcn2 expression in PDGFRα^+^ BMMSCs further support the notion that the Klf2-Ccl2-Lcn2 signaling axis mediates Piezo1 deficiency-induced bone loss and bone marrow adiposity in mice.

## Discussion

This study shows that Piezo1 invalidation in BMMSCs activates an autocrine inflammatory axis involving Ccl2 and Lcn2 through CaMKII/Klf2 and promotes bone marrow adipogenesis, while inhibiting bone formation. Importantly, our findings also indicate that Piezo1 invalidation abolishes exercise-induced benefits on bone volume and marrow adiposity, suggesting that Piezo1 senses physiological mechanical stress (presumably shear stress and compressive forces) in the bone marrow to regulate BMMSC fate decision (Fig. 8h). Thus, our observations bridge the concepts of mechanosensing, inflammatory cytokine secretion, and cell fate determination of BMMSCs, highlighting Piezo1 as a pivotal hub that coordinates these interconnected processes and its critical role in conferring the beneficial effects of physical exercise on bone health.

The cell fate decision of BMMSCs is a complex, tightly regulated process involving the integration of multiple signaling pathways, transcription factors, and extracellular cues.^2^ The diversity of the bone marrow niche renders BMMSCs particularly susceptible to microenvironmental factors, including matrix stiffness, mechanical forces, and geometric configurations, which are known to significantly impact their lineage commitment.^2^ For example, spread-shaped BMMSCs are prone to differentiate into osteoblasts, whereas round cells tend to become adipocytes.^57^ Moreover, the rigidity of the substrate controls BMMSC differentiation, with bone cells forming on a stiff substrate and adipocytes forming on a soft substrate.^2^ Even subtle alterations in the physical landscape, such as nanoscale geometric cues, can effectively dictate BMMSC differentiation.^58^ Osteopontin, an extracellular matrix component, has been shown to interact with integrin αv/β1 to regulate the adipo-osteogenic balance of BMMSCs.^59^ However, the cellular machinery that influences the dynamic extracellular microenvironment to finetune the lineage commitment of BMMSCs in vivo remains largely unknown. In this study, we identify the mechanosensitive ion channel Piezo1 as a key cell surface sensor for mechanical input associated with physical activity to shift the balance of BMMSC differentiation from adipogenesis to osteogenesis. In addition to its ability to inhibit adipogenesis and conversely induce osteoblast formation in BMMSCs, Piezo1 was also shown to increase bone formation and bone density via its direct actions in osteoblasts and osteoclasts.^20–23^ Taken together, these findings raise the possibility of using pharmacological activators of Piezo1 as “exercise mimetics” to improve skeletal fitness and bone health.

BMMSCs secrete a wide array of bioactive molecules that play critical roles in immunomodulation and tissue regeneration, including chemokines, cytokines, and growth factors.^60^ BMMSC-secreted factors have been exploited for the treatment of multiple human diseases, such as refractory autoimmune disease, bone fracture, and tissue degeneration.^60^ However, the molecular and cellular basis underlying the pleiotropic paracrine actions of BMMSCs remains poorly defined. In this study, we identify BMMSC-secreted Lcn2 as the key downstream effector of Piezo1, below CaMKII/Klf2, which acts in an autocrine/paracrine manner to modulate cell commitment to adipocytes or osteoblasts. Lcn2, which was first identified as a top responder to endotoxin in macrophages, is an inflammatory factor closely associated with obesity-related metabolic complications and the innate immune response to bacterial infection.^61–63^ It also exacerbates nonalcoholic steatohepatitis by promoting neutrophil-macrophage crosstalk and activating hepatic stellate cells.^64^ Notably, Lcn2 has also been proposed as a mechanoresponsive gene upregulated by mechanical underload, exerting negative effects on bone cell formation.^45^ A large prospective cohort study revealed a strong positive association between high plasma Lcn2 levels and the risk of fracture and osteoporosis.^65^ Intriguingly, recent bioinformatic analyses have inferred a possible connection between Piezo1 and Lcn2, predicting an upsurge in Lcn2 expression in bone tissues lacking Piezo1.^23,47^ Despite these correlations and predictions, experimental evidence for a possible functional link between Piezo1 and Lcn2 is not yet available. Our study provides both in vitro and in vivo evidence that Lcn2 is strongly induced by Piezo1 invalidation, thereby promoting adipogenesis and inhibiting bone formation. Importantly, we found that antibody-based blockade of Lcn2 reversed osteoporosis and marrow adiposity in Piezo1-deficient mice, suggesting that pharmacological/genetic inhibition of BMMSC-secreted Lcn2 may represent a promising pharmacotherapy for mechanical unloading-induced bone diseases. Mechanistically, the effect of Lcn2 in shifting the commitment of BMMSCs from osteogenesis to adipogenesis is thought to be related to its ability in modulating PPARγ activation and expression. In line with our data, increased PPARγ activation and expression by Lcn2 have been reported in several previous studies.^66,67^ However, given that the molecular identity of the Lcn2 receptor remains obscure, how Lcn2 exerts its autocrine effects on the differentiation of BMMSCs upon Piezo1 invalidation requires further investigation.

Previous studies on BMMSC-derived Ccl2 primarily focused on its paracrine effects on facilitating macrophage recruitment and M2 polarization,^68^ suppressing CD4^+^ T-cell activation and promoting myeloid cell differentiation,^69,70^ enhancing anti-tumor properties in prostate cancer,^71^ and accelerating wound healing.^72^ Here, we show that BMMSC-secreted Ccl2 induces Lcn2 production in an autocrine manner, thereby shifting cell commitment from osteoblasts to adipocytes. Using a multifaceted approach that includes in silico analysis across four different databases and chromatin immunoprecipitation, complemented by the application of the NF-κB inhibitor QNZ, we demonstrate that Ccl2-induced Lcn2 expression is mediated by NF-κB-induced transcriptional activation. Our results position Ccl2 as an upstream regulator that links Piezo1 deficiency with sustained proinflammatory signaling via NF-κB activation and Lcn2 expression, thus promoting adipogenesis and suppressing osteogenesis. Our findings are also in line with several previous studies demonstrating Ccl2-induced Lcn2 expression in Snail (+) tumor cells,^48^ NF-κB activation by Ccl2 in osteoclasts,^50^ and the critical role of NF-κB in mediating TNFα-induced Lcn2 expression in prostate cancer cells and adipocytes.^73^ The augmented NF-κB activation observed in our Piezo1 KO BMMSCs is also in accordance with a previous report showing that Piezo1 is a negative regulator of NF-κB activation in microglia.^74^ Furthermore, we show a markedly increased Ccl2 production attributed to the increase in c-Jun-mediated gene transactivation in Piezo1 KO BMMSCs, suggesting that Ccl2 is also a mechanoresponsive factor suppressed by Piezo1 activation. Additionally, we found that the effect of Piezo1 opening on BMMSC cell fate determination is mediated through the CaMKII-Klf2 axis. Considering the negative role of Klf2 in regulating NF-κB and AP-1,^53,55^ together with the effect of CaMK inhibition or a low-calcium environment on c-Jun/AP-1 activation in neurons or osteoblastic cells,^75,76^ Piezo1 invalidation likely activates c-Jun/AP-1 and NF-κB by repressing CaMKII activity and Klf2 expression.

BMAs represent a unique adipose depot distinct from peripheral adipocytes.^77^ Our study shows that Piezo1 exerts a specific effect in BMMSCs by preventing adipogenesis, although it does not influence fat formation in white adipose tissue (WAT) when invalidated in PDGFRα^+^ cells. In contrast, it is interesting to note that Piezo1 invalidation (using adiponectin Cre as a driver) in differentiated adipocytes prevents obesogenic adipogenesis, further enhancing adipocyte hypertrophy in response to a high-fat diet.^78^ Thus, our study reveals a unique role for Piezo1 in BMMSCs, acting as a key inhibitor of adipogenesis, unlike its stimulatory role in mature adipocytes of WAT. Owing to their specific anatomical location, BMAs play important regulatory roles in bone formation/remodeling, hematopoiesis, immune responses, and metabolism via their paracrine actions to modulate the bone marrow microenvironment. The secretion profiles of BMAs are characterized by increased production of proinflammatory cytokines.^79^ Excessive accumulation of BMAs, which occurs with aging, obesity and other clinical conditions, such as anorexia and cancer patients receiving chemotherapy or radiotherapy, may disrupt the bone marrow environment, leading to chronic inflammation, cellular senescence, and impaired immune fidelity.^80^ However, the function and regulation of BMAs remain understudied due to the technical difficulties in selectively manipulating BMAs without affecting other peripheral adipose depots. Our present study shows that genetic ablation of Piezo1 in PDGFRα-expressing cells causes selective expansion of BMAs without any significant change in peripheral adipose depots, although both BMMSCs and peripheral adipocyte progenitor cells express PDGFRα and Piezo1.^25,26^ This finding is further corroborated by our transplantation study demonstrating that wild-type recipient mice reconstituted with bone marrow cells from PDGFRα-Piezo1 KO donor mice recapitulate the phenotypic changes observed in the KO mice, further indicating that distinct regulatory mechanisms govern adipogenesis in BMAs and peripheral adipose tissues. Thus, our mouse model provides a valuable tool for further interrogating the distinct pathophysiological functions of BMAs in the context of aging and obesity.

In line with our findings, emerging clinical evidence suggests that the Piezo1-Klf2-Ccl2-Lcn2 axis is altered in patients with osteoporosis. Reduced *PIEZO1* expression in bone tissue has been shown to be correlated with lower bone mineral density and impaired bone quality across multiple osteoporosis subtypes, including glucocorticoid-induced osteoporosis and age-associated osteoporosis.^20,81,82^ A genetic study revealed the close association of a *PIEZO1* gene polymorphism (SNP rs62048221) with human bone density, possibly by modulating *PIEZO1* gene expression.^83^ In contrast, the upregulation of *KLF2* in hBMMSCs has been shown to promote osteogenic differentiation.^84^ Likewise, elevated CCL2 levels are observed in osteoporosis patients and are inversely associated with bone loss in individuals with postmenopausal osteoporosis.^85,86^ The CCL2 (MCP-1) A2518G and CCR2 V64I gene variants are risk factors for osteoporosis and osteopenia.^87^ Similarly, elevated circulating LCN2 levels are associated with increased fracture risk in elderly women, although its relationship with bone turnover may differ between healthy and osteoporotic populations.^65,88^ These clinical evidences suggest that pharmacological interventions targeting Piezo1-Klf2-Ccl2-Lcn2 axis might be effective for the treatment of osteoporosis in humans.

Collectively, our findings reveal that the mechanosensor Piezo1 acts as a key gatekeeper to tune down the c-Jun-Ccl2-NF-κB-Lcn2 inflammatory axis through CaMKII/Klf2, thereby balancing the adipo-osteogenic potential of BMMSCs. Mechanical unloading or Piezo1 inactivation disrupts this balance, leading to excessive activation of the Ccl2-Lcn2 autocrine loop in BMMSCs (because of lower Klf2 expression) and enhanced bone marrow adipogenesis, which in turn contributes to aging- or obesity-related skeletal and/or metabolic disorders. Conversely, physical exercise exerts its health benefits on augmentation of bone density and reduction of BMAs by inhibiting this autocrine inflammatory Ccl2-Lcn2 loop via Piezo1 opening and downstream activation of the CaMKII/Klf2 pathway. In this context, BMAs correspond to a default state that can be repressed by mechanical activation of Piezo1 in BMMSCs to become bone cells. These findings uncover a critical role of mechano-controlled inflammation in the cell fate decision of BMMSCs and raise the possibility of targeting the Piezo1-c-Jun-Ccl2-NF-κB-Lcn2 axis as a promising strategy for the development of BMMSC-based therapies for aging- and obesity-related chronic disorders.

## Materials and methods

### Animal models

Piezo1^flox/flox^ mice were generated as previously described^89–91^ and were then bred with mice expressing the Cre transgene under the control of PDGFRα (PDGFRα-Cre mice, The Jackson Laboratory). Heterozygous Piezo1^flox/wt^ mice with or without PDGFRα-Cre transgene expression were then bred to generate homozygous Piezo1^flox/flox^ mice with (PDGFRα-Piezo1 KO) or without Cre (WT control) expression. These homozygous mice were backcrossed onto a C57BL/6J genetic background for at least ten generations. PDGFRα-Cre tdTomato reporter mice were generated by crossing tdTomato fluorescent reporter (Ai14) mice with PDGFRα-Cre driver mice. Mice of the same genotype were randomly assigned to each group. All the mice were housed in a controlled facility (22 ± 1 °C, 12 h light/dark cycle, 60–70% humidity) with *ad libitum* access to water and either a standard chow diet (LabDiet 5053) or a high-fat diet (45 kcal% fat, D12451, Research Diet, USA). Fat percentage and lean percentage were determined by the Bruker Minispec LF90 body composition analyzer. Oxygen consumption and the respiratory exchange ratio (RER) were measured via metabolic chambers (Columbus Instruments). The animals were euthanized via cardiac puncture after anesthesia at the end of the experiments. All the animal experiments were approved by the Committee on the Use of Live Animals in Teaching and Research (CULATR, #5184-19, #23-482, #23-479) at the University of Hong Kong. Sample size determination is based on previous experience to obtain significance and reproducibility,^92,93^ as well as minimizing the number of animals used as required by the animal ethics committee. The sample size followed common standards and employed four or more biological replicates. All sample sizes are listed in each figure legend. Animals that failed to reach the experimental endpoints due to premature mortality or veterinary-mandated euthanasia were excluded prospectively per predefined criteria.

### Micro-computed tomography (micro-CT) analysis

Mouse tibias were excised and fixed with 4% paraformaldehyde solution (PFA) and then subjected to micro-CT scan using the Skyscan1076 micro-CT system (Bruker, Kartuizersweg, Belgium). The region of interest was 100 to 200 slices below the growth plate, and the following parameters were analyzed using CTAn software (Bruker): bone volume fraction (BV/TV), trabecular number (Tb.N), trabecular spacing (Tb.Sp), trabecular thickness (Tb.Th), and bone mineral density (BMD). Three-dimensional images were reconstructed by stacking the two-dimensional slices from the selected regions using CTVox software (Bruker).

### Osmium tetroxide staining

Osmium tetroxide, a radiopaque chemical compound which stains unsaturated lipids and lipoproteins, was used to visualize and quantify bone marrow fat using micro-CT.^32^ Tibias were decalcified in 0.5 M EDTA (pH = 7.4) for 21 days after micro-CT scanning, and then washed twice with distilled water and once with Sorensen’s phosphate buffer (pH = 7.4) for 5 min each. The decalcified tibias were transferred to 1.5 mL tubes containing 300 μL of Sorensen’s buffer. Another 300 μL of 2% osmium tetroxide solution was added to each tube to make a 1% solution. The specimens were stained in the solution for 48 h at room temperature on a shaking platform. The stained tibias were washed for three times with 1 mL of Sorensen’s buffer for 3 h each at room temperature on a shaking platform. The stained tibias were embedded in 1% low-melting agarose (Invitrogen, #16520100) and placed in a fresh set of 1.5 mL microtubes. The content of BMAs was measured using a Skyscan1076 micro-CT system. The region of interest was the entire tibia. The percentage of BMAs was calculated by dividing the osmium-stained adipose volume by the total marrow volume of the whole tibia (AV/TV) using CTAn software.

### In vivo alizarin red and calcein double labeling

To evaluate dynamic periosteal bone formation, 6-week-old male WT, PDGFRα-Piezo1 KO, and PDGFRα-Cre mice received intraperitoneal injections of calcein (20 mg/kg, Sigma-Aldrich #C0875) and Alizarin Red S (40 mg/kg, Sigma-Aldrich #A5533) with a 7-day interval. The mice were euthanized 48 h after the second injection, and tibias were dissected, fixed in 4% PFA, dehydrated in graded ethanol, and embedded in polymethylmethacrylate. Transverse sections of the mid-diaphysis were imaged using a confocal laser scanning microscope (Carl Zeiss LSM 880) with sequential fluorescence excitation to distinguish calcein (488 nm) and alizarin (561 nm) signals. The mineral apposition rate (MAR) was determined by measuring the average distance between fluorescent labels, while the bone formation rate (BFR) was calculated as MAR × MS (mineral surface)/BS (bone surface).

### Differentiation and treatment of mouse BMMSCs

The protocol for isolation and culture of BMMSCs were adhered strictly to standard protocols.^94^ Briefly, 10-week-old male WT and PDGFRα-Piezo1 KO mice were deeply anesthetized with 5% pentobarbital sodium (Dorminal, #013003) and sacrificed by cervical dislocation, followed by immersing in 75% ethanol for 3 min and isolation of femurs and tibias under sterile condition. The bones were then cut at both ends, and the bone marrow was flushed out using a 5 mL syringe equipped with a 25 gauge needle filled with alpha modified Eagle’s minimum essential medium (α-MEM) (Gibco, #11900024). The cells were then filtered through a 70 μm cell strainer (Corning, #352350). After 3 h, remove the non-adherent cells that accumulate on the surface of the dish by changing the medium and replacing with fresh complete medium. The MycoBlue Mycoplasma Detector (Vazyme, #D101) was used to detect mycoplasma in BMMSCs according to the manufacturer’s instructions. If the reaction solution remains purple, it is determined to be mycoplasma negative; if the reaction solution changes to sky blue, it is determined to be mycoplasma positive.

Mycoplasma negative BMMSCs were grown in the α-MEM medium containing 10% fetal bovine serum (FBS) (Gibco, #10270106) and 1% Penicillin/Streptomycin/Amphotericin B solution (Sigma, #P3032, #S1277, #A9538). When the cells reached 65–70% confluence, subculture the adherent BMMSCs. The differentiation of BMMSCs into osteoblasts was initiated once the subcultured BMMSCs reached 80% confluence using the osteogenic induction medium containing 100 nM dexamethasone (Sigma, #D2915), 50 μg/mL ascorbic acid (Aladdin, #S105026), and 10 mM β-glycerophosphate (Sigma, #G9422). The medium was then replaced every 4 days until day 21. For the adipogenic differentiation, BMMSCs with 100% confluency were treated with the adipogenic induction medium containing 1 μM dexamethasone, 0.5 mM 3-Isobutyl-1-methylxanthine (IBMX) (Sigma, #I5879), 1 μM rosiglitazone (Supelco, # PHR2932), and 1.8 μM insulin (Actrapid, #23103007). After 48 h, the medium was changed to a maintenance medium, *i.e.*, α-MEM containing 1 μM dexamethasone, 1 μM rosiglitazone, and 1.8 μM insulin. The maintenance medium was replaced every 2 days until day 8.

To explore the effects of the Ccl2/CCR2 axis on BMMSC differentiation, 100 ng/mL of recombinant mouse Ccl2 protein (PeproTech, #250-10) or 10 nM CCR2 antagonist INCB3344 (MCE, #HY-50674) was supplemented into the adipogenic and osteogenic induction medium of WT or Piezo1 KO BMMSCs, respectively. To investigate the roles of BMMSC-secreted Lcn2, BMMSCs were either infected with 40 multiplicity of infection (MOI) of lentivirus encoding short hairpin RNA (shRNA) against *Lcn2* (Lenti-shLcn2, target sequence: CAGGCAATGCGGTCCAGAAAAA) or scrambled shRNA (Lenti-sc, target sequence: CCTAAGGTTAAGTCGCCCTCG) (OBiO Technology) along with 5 μg/mL of polybrene (OBiO Technology, used for enhancing viral transduction efficiency) for 72 h, or treated with 200 ng/mL of rabbit anti-mouse Lcn2 antibody (raised using full-length mouse Lcn2 as an immunogen^64^) (ImmunoDiagnostics Limited, #12050) or non-immune rabbit IgG in the adipogenic and osteogenic induction medium. To investigate the regulatory effects of AP-1 inhibitor on Ccl2, 10 μM AP-1 inhibitor T-5224 (MCE, #HY-12270) was supplemented into the medium of WT or Piezo1 KO BMMSCs for 24 h. To investigate the regulatory role of NF-κB on Lcn2 expression, WT and PDGFRα-Piezo1 KO BMMSCs were first treated with recombinant mouse Ccl2 protein (rmCcl2, 100 ng/mL) and CCR2 antagonist INCB3344 (10 nM) for 24 h, followed by supplementation with the NF-κB-specific inhibitor QNZ (10 nM, MCE, #HY-13812) for additional 2 h.

To investigate the effects of Klf2 on BMMSC differentiation, 40 MOI of lentivirus encoding *eGFP* or *eGFP* together with *Klf2* (WZ Biosciences Inc) was used to infect WT or Piezo1 KO BMMSCs for 72 h with the presence of 5 μg/mL polybrene before differentiation. To explore the role of CaMKII in regulating Piezo1-dependent Klf2 expression, WT BMMSCs were treated with 10 μM CaMKII inhibitor KN-93 (MCE, #HY-15465) and/or 5 μM Piezo1 activator Yoda1 (MCE, #HY-18723) for 2 h.

### Alizarin Red S and Oil Red O staining

After 21 days of osteogenic induction, cells were washed once with phosphate-buffered saline (PBS), fixed with 10% PFA for 30 min at room temperature, and were subsequently stained with 2% Alizarin Red S (ARS) solution (Sigma, #A5533, adjusted to pH = 4.1–4.3) for 15 min at room temperature. The excess stain was removed by washing with PBS. Calcium deposits were visualized as red staining under a light microscope (Olympus, #ckx53). For quantification, the attached ARS were then destained with 10% cetylpyridinium chloride (Sigma, #C0732) as previously described.^95^ The absorbance was measured at 405 nm using a spectrophotometer.

To visualize the lipid content in adipocytes, cells were fixed with 4% PFA for 15 min at room temperature, washed for three times with PBS (5 min each) and then stained with Oil Red O solution (Sigma, #O0625) for 15 min at room temperature. After staining, the Oil Red O dye was extracted from the stained lipids using 100% isopropanol (500 µL), and the absorbance of this solution was measured at 520 nm with a spectrophotometer to quantify the lipid content.

### Bone marrow transplantation

Ten-week-old C57BL/6J male recipient mice were pretreated with enrofloxacin (Baytril) (0.17 mg/mL) in drinking water for 2 weeks and were then subjected to 9 Gy total body irradiation (split into 2 doses separated by 4 h to minimize gastrointestinal toxicity) using MDS Nordion Gammacell 3000 Elan irradiator to deplete bone marrow. In the following day, bone marrow cells were isolated from femurs and tibias of 6-week-old male donor mice (PDGFRα-Piezo1 KO mice and their WT littermates, or PDGFRα-Cre tdTomato reporter mice) by flushing out the bone marrow with a solution of 10 mL of Hanks’ balanced salt solution (HBSS) (Gibco, #14025092) containing collagenase type IV (10 mg, Gibco, #17104019) and dispase (20 mg, Gibco, #17105041) and were incubated at 37 °C for 10 min.^96^ Cells were then filtered through a 70 μm cell strainer and resuspended within HBSS at a concentration of approximately 1 × 10^7^ cells/0.2 mL for tail vein injection into the irradiated recipient mice. After bone marrow transplantation, the recipient mice were treated with enrofloxacin (0.17 mg/mL) in drinking water for another 2 weeks.

### Treadmill exercise

Eight-week-old male PDGFRα-Piezo1 KO mice and their WT littermates were acclimated to treadmill (LE8710M, Panlab, Spain) running at a low speed for 2 days as previously described^97^ and then subjected to treadmill exercise at a speed of 15 m/min for 30 min per day. The mice were trained 5 days per week for 6 weeks. Another two age-matched groups without exercise were used as sedentary controls.

### Conditioned medium treatment

BMMSCs isolated from femurs and tibias of 10-week-old male PDGFRα-Piezo1 KO or WT mice were cultured in α-MEM until they reached 80–100% confluence. Conditioned medium was collected from the cells cultured in serum-free medium for 24 h. BMMSCs isolated from femurs and tibias of 10-week-old male C57BL/6J WT mice were treated with half of the adipogenic or osteogenic induction medium and half of the conditioned medium from Piezo1 KO or WT BMMSCs during the induction period. The adipogenic induction medium was changed every 2 days for 8 days, while the osteogenic induction medium was changed every 3 days for 21 days.

### Alkaline phosphatase staining for cells

After 21 days of osteogenic induction, cells were washed with PBS, fixed with 10% PFA for 30 min at room temperature. Subsequently, cells were stained using an alkaline phosphatase (ALP) staining kit (Sigma, #86R) for 30 min at 37 °C. The staining reaction was terminated by washing with distilled water, and the stained cells were observed under a light microscope.

### Olink proteomics

The Olink Target 96 Mouse Exploratory Probe Kit (Olink, Sweden, #95380A) was employed to assess the protein secretion profile of mouse BMMSCs. Once the cells reached 80% confluence, the culture medium was replaced with serum-free α-MEM and grown for another 24 h. The conditioned medium was collected for analysis using the Olink Signature Q100 system (Olink, Sweden). The Olink data was presented by normalized protein expression (NPX) values on a log2 scale, which was then transformed to linear NPX by using the formula 2^NPX. Data with a missing frequency larger than 75% were excluded from the analysis.^98^

### Enzyme-linked immunosorbent assay (ELISA)

Conditioned medium was harvested from WT and KO BMMSCs after 24 h of serum-free culture. Fabp4 (ImmunoDiagnostics Limited, #32030), Lcn2 (ImmunoDiagnostics Limited, #32050), and Ccl2 (R&D Systems, #MJE00B) levels in the conditioned medium were detected by ELISA according to the manufacturer’s instructions and were normalized using protein concentrations determined by bicinchoninic acid (BCA) assay (Thermo Scientific, #23225). Serum levels of Lcn2 and Ccl2 were also quantified by ELISA in PDGFRα-Piezo1 KO mice or WT mice, and mice injected with either rabbit non-immune IgG or anti-Lcn2 neutralizing antibody. For the measurement of adiponectin and leptin levels in the conditioned medium of scWAT explants, serum-free conditioned medium after 24-h culture was collected and subjected to ELISA analysis of adiponectin (ImmunoDiagnostics Limited, #32010) and leptin (R&D Systems, #MOB00B) according to the manufacturer’s instructions and were normalized to tissue weight. For ELISA analysis to quantify p65 subcellular localization, BMMSCs were harvested and subjected to separation into the nuclear and cytoplasmic proteins according to the manufacturer’s instructions (Beyotime, #P0028). The nuclear and cytoplasmic fractions were then assayed for NF-κB p65 using ELISA kits (Abcam, #ab176648) following the manufacturer’s instructions.

### Chromatin immunoprecipitation (ChIP) assay

BMMSCs were crosslinked using 37% PFA (final concentration 1.42%) in culture medium for 15 min at room temperature, followed by quenching with 1 M glycine solution (final concentration 125 mM) for 5 min. The cells were subsequently collected and washed thrice with cold PBS. Chromatin shearing was achieved through sonication at 25% amplitude for 5 min in ChIP buffer according to a previous established protocol,^99^ alternating between 10-s on and 10-s off sonication frequency. After centrifugation, the supernatant was subjected to DNA isolation as input or immunoprecipitation at 4 °C for overnight with rabbit anti-NF-κB p65 monoclonal antibody (Cell Signaling Technology, #8242, 1:100), c-Jun (Cell Signaling Technology, #9165, 1:50), STAT1 (Cell Signaling Technology, #9172, 1:50), STAT3 (Cell Signaling Technology, #9139, 1:100), SPI1 (Santa Cruz Biotechnology, sc-390405, 1:100), or rabbit non-immune IgG (50 μg/mL). Protein A/G agarose beads (YEASEN, #36404ES60) were then added to incubate with samples for 2 h at 4 °C with rotation. The samples were then washed and eluted using ChIP buffer (excluding NP-40 and Triton-X) and an elution buffer containing 1% SDS and 100 mM NaHCO₃ in distilled water. The protein-DNA complexes in the elution fraction were reverse crosslinked by incubation with 5 M NaCl for 4 h at 65 °C. DNA was then precipitated using absolute ethanol and dissolved in distilled water. To quantify the enrichment efficiency of the chromatin immunoprecipitation, 1 μL DNA sample was used to amplify the predicted promoter region by qPCR. The enrichment of the transcription factor on the targeting region was accessed by calculating the value of “% of input” using the formula: % of Input = 2^(CT^Input^−CT^IP^)/dilution factor × 100%. The input served as a positive control, while IgG was used as a negative control. The amplified products were collected for agarose gel electrophoresis. The band intensities were quantified using ImageJ software (v1.53). Uncropped films of agarose gel are provided in Supplementary Information.

### Luciferase reporter assay

The luciferase reporter assay was performed by transfecting 0.2 μg of firefly and Renilla luciferase reporter vectors (Addgene, #128046) driven by either the wild-type mouse Lcn2 or Ccl2 promoter (−1999/+1) or their respective mutants (in which the NF-κB or c-Jun DNA binding site was mutated) (Azenta) into BMMSCs seeded in 96-well plates using Lipofectamine 3000 transfection reagent (Thermo Fisher Scientific, #L3000001). After 48-h incubation post-transfection, cells were lysed with Passive Lysis Buffer (Promega, #E1500) and luciferase activities were quantified using the CLARIOstar microplate reader (BMG LABTECH, #0430-101) with dual-luciferase detection capabilities. Firefly luciferase activity values were normalized against Renilla luciferase readings to account for transfection efficiency variations, and relative promoter activities were expressed as the ratio of firefly to Renilla luminescence signals.

### Immunofluorescence staining

Cryosections of the brain, quadriceps, gastrocnemius, and soleus from 8-week-old male PDGFRα-Piezo1 KO mice or WT mice, or PDGFRα-Cre tdTomato reporter mice, along with cultured BMMSCs and scWAT-derived SVFs, were fixed in 4% PFA for 15 min. After PBS washing, samples were blocked with 3% bovine serum albumin (BSA, Sigma, #A7906) in PBS for 1 h, then incubated with rabbit anti-Piezo1 antibody (Proteintech, #15939-1-AP, 1:300) or anti-NF-κB p65 rabbit monoclonal antibody (Cell Signaling Technology, #8242, 1:250) for overnight at 4 °C. Following PBS washing for three times, sections and cells were incubated with Alexa Fluor 488-conjugated goat anti-rabbit IgG (Invitrogen, #A-11008, 1:500) for 1 h at room temperature. Nuclei were counterstained with 4’,6-diamidino-2-phenylindole (DAPI) (Thermo Fisher, #62248, 1:500) for 5 min. After final PBS washing, slides were mounted with antifade medium (Abcam, #ab104135). Imaging was performed on a ZEISS LSM 880 confocal microscope. Piezo1-positive signal intensity (green channel) was quantified in ≥5 fields/sample using ZEN Lite software (v3.12). P65 nuclear translocation was quantified using Pearson’s correlation coefficient (PCC), calculated in ImageJ (v1.53) between p65 immunofluorescence and DAPI signals across segmented nuclear regions.

### Intra-tibial injection of lentivirus

Six-week-old male WT and PDGFRα-Piezo1 KO mice were anesthetized via intraperitoneal injection of ketamine/xylazine and administered with 1.25 × 10^6^ transducing units (TU) lentiviruses bearing the PDGFRα promoter-driven gene expression (WZ Biosciences Inc) through bilateral intra-tibial injection into the tibial bone marrow cavity using 1 mL insulin syringes (BD Biosciences). Four different lentiviruses were used in this study, including scrambled shRNA as control (Lenti-PDGFRα-eGFP-scramble), shRNA-mediated *Ccl2* knockdown (Lenti-PDGFRα-eGFP-shCcl2), shRNA-mediated *Lcn2* knockdown (Lenti-PDGFRα-eGFP-shLcn2), and *Klf2* overexpression (Lenti-PDGFRα-eGFP-oe Klf2). Sequences of shRNAs were listed in supplementary Table 2. Under aseptic surgical conditions, 50 μL of viral suspension was delivered in each injection site. Seven days post-injection, in vivo transduction efficiency was assessed using the IVIS (In vivo imaging system) Spectrum imaging system (PerkinElmer) with 480/520 nm filters.

### Immunohistochemical staining for alkaline phosphatase

Deparaffinized and rehydrated bone sections (~5 µm thick) were subjected to antigen retrieval in citrate buffer (10 mM, pH 6.0) via microwave irradiation (95 °C, 15 min), followed by 30 min cooling at room temperature. Endogenous peroxidase activity was blocked with 3% H₂O₂ (30 min, dark). After PBS washing (5 min × 3 times), sections were incubated with 10% normal goat serum for 30 min at room temperature to block non-specific binding and then incubated with alkaline phosphatase recombinant rabbit monoclonal antibody (HUABIO, #ET1601-21, 1:1600) for overnight at 4 °C. Following PBS washing, sections were treated with HRP-conjugated goat anti-rabbit IgG polyclonal antibody (HUABIO, #HA1001, 1:500) for 1 h at room temperature. Signal detection employed ultra- diaminobenzidine (DAB) substrate kit (Maxim, #DAB-4033; 5 min at room temperature), with reaction termination in distilled water. Sections were counterstained with hematoxylin (Solarbio, #G1120; 30 s), dehydrated in ethanol, and mounted with clean and environmental mounting medium (Biosharp, #BL1519A).

### Hematoxylin and eosin (H&E) staining

Tibias were fixed in 4% PFA solution for 24 h and then decalcified in a 0.1 M ZnSO_4_ and 0.5 M EDTA (pH 7.4) solution in a decalcification instrument (Milestone) for 48–72 h. The samples were subsequently dehydrated and embedded in paraffin wax using the EG1150 paraffin embedding station (Leica Biosystems). Adipose tissues, skeletal muscles, and decalcified bone sections were deparaffinized and rehydrated as described before.^92^ For H&E staining, the sections were stained with hematoxylin for 2–3 min. The sections were then differentiated in 1% acid alcohol (1% hydrochloric acid in 70% ethanol) for a few seconds to remove excess hematoxylin. After differentiation, the sections were rinsed in running tap water and subsequently stained with eosin for 2 min. The sections were then dehydrated through absolute ethanol solutions and cleared in xylene. Finally, the sections were mounted with coverslips and examined under a light microscope.

### RNA preparation and real-time PCR

Total RNA was extracted from BMMSCs using RNAiso Plus (Takara, #9108) and reverse transcribed into complementary DNA using PrimeScript RT reagent kit (Takara, #RR037A). Quantitative real-time PCR was performed using TB Green® Premix Ex Taq (Takara, #CN830A) with specific primers as listed in supplementary Table 3 on a StepOne System (Applied Biosystems). The relative gene expression level was normalized to the *β-actin* gene.

### Statistical analysis and reproducibility

All the data were statistically analyzed via GraphPad Prism 9.5 software (GraphPad, San Diego, CA) and are presented as the means ± standard errors of the means (SEMs). Statistical tests were chosen based on data distribution and variance. For normally distributed data with equal variances, an unpaired two-tailed Student’s t test was used for pairwise comparisons, one-way analysis of variance (ANOVA) with Tukey’s test for multiple groups with a single variable, or two-way ANOVA for comparisons with multiple variables. For normally distributed data with unequal variances, an unpaired t test with Welch’s correction or Welch ANOVA was applied. Nonparametric tests, the Mann–Whitney test or the Kruskal-Wallis test, were used for nonnormally distributed data or unequal variances. A *p* value of <0.05 was considered statistically significant. *P* values and *n* values are indicated in the associated figure legends for each figure. Investigators were blinded to group allocation during data collection.

## Supplementary information

Supplemental Material

Uncropped blot and gel

Source data file for main figures

Source data file for supplementary figures

## Data Availability

All the data generated or analyzed during this study are included in this article and its supplementary information.
