# Supplementary material for: Piezo1 activation suppresses bone marrow adipogenesis to prevent osteoporosis by inhibiting a mechanoinflammatory autocrine loop
Source: Signal Transduct Target Ther. 2025 Oct 28;10:357. doi: 10.1038/s41392-025-02455-w (PMC12559722; doi:10.1038/s41392-025-02455-w)
Supplement: Supplementary file 1 — Supplemental Material [file 41392_2025_2455_MOESM1_ESM.docx]

Supplementary Materials for

**Piezo1 activation suppresses bone marrow adipogenesis to prevent osteoporosis by inhibiting a mechanoinflammatory autocrine loop**

Baile Wang^1,2,3#^*, Jie Liu^1,2,3#^, Qin Wang^1,2,3^, Malika Arhatte^4^, Lai Yee Cheong^1,2^, Edyta Glogowska^4^, Xue Jiang^1,2,3^, Sookja Kim Chung^1,5^, Leigang Jin^1,2,3^, Qianxing Hu^1,2,3^, Yu Wang^1,6^, Eric Honoré^4,6^*, Aimin Xu^1,2,3,6^*

# These authors contributed equally to this work.

* Corresponding authors: [amxu@hku.hk](mailto:amxu@hku.hk); [honore@ipmc.cnrs.fr](mailto:honore@ipmc.cnrs.fr); [blwong@connect.hku.hk](mailto:blwong@connect.hku.hk)

**This PDF file includes:**

Materials and Methods

Figures. S1 to S18

Tables S1 to S3

**Materials and Methods**

**Differentiation and treatment of human BMMSCs (hBMMSCs)**

Mycoplasma negative hBMMSCs purchased from Lonza (#PT-2501) were cultured in α-MEM supplemented with 10% FBS and 1% penicillin/streptomycin/amphotericin B solution until reaching 80% confluence. The osteogenic differentiation of hBMMSCs was induced using the same cocktails as described for mouse BMMSCs. For adipogenesis, as previously described,^1^ hBMMSCs were cultured in full α-MEM medium until 100% confluence was reached. The cells were then exposed to an induction medium consisting of Dulbecco's modified eagle medium (DMEM) supplemented with 10% FBS, 10 µg/mL insulin, 1 μM dexamethasone, 0.5 mM IBMX, and 200 μM indomethacin for 48 hours, followed by a transfer to maintenance medium (DMEM supplemented with 10 µg/mL insulin and 10% FBS). This induction and maintenance cycle was repeated twice. Afterwards, the cultures were sustained in the maintenance medium and the medium was replaced every 2 days until day 14.

**Tartrate-Resistant Acid Phosphatase (TRAP) staining**

Deparaffinized and rehydrated bone sections (~5 µm thick) were stained for TRAP using the TRAP staining kit (Sigma, #387A) according to the manufacturer's protocol. The osteoclast surface was measured using TrapHisto software.

**Multiplex assay**

The Mouse Luminex® Discovery Assay (R&D Systems, #LXSAMSM) was used to measure the concentrations of cytokines Ccl7, Cxcl12 and Igf-1 in the 24-hour serum-free conditioned medium from both WT and KO BMMSCs. All samples were analyzed using the Luminex 200 system according to the manufacturer’s instructions. The concentration of cytokines was normalized to the total protein content of the cells, which was determined using the BCA assay.

**Measurement of tdTomato fluorescence intensity in tissues**

Fresh tissues including subventricular zone, white matter, corpus callosum, cortex, hypothalamus, quadriceps, gastrocnemius, soleus, scWAT, eWAT, BAT, spleen, and kidney were isolated from PDGFRα-Cre tdTomato reporter mice and weighed. Bone marrow was flushed with ice-cold PBS and subjected to erythrocyte lysis using ammonium-chloride-potassium (ACK) lysis buffer (150 mM NH₄Cl, 10 mM KHCO₃, 0.1 mM EDTA), followed by centrifugation (300 ×g, 5 min) and two PBS washes. All samples were homogenized in PBS using a mechanical homogenizer (1,300 rpm, 5 min, 4°C), centrifuged (13,000 g, 15 min), and supernatants transferred to black 96-well plates. tdTomato fluorescence was quantified on a BMG CLARIOstar Plus microplate reader (excitation/emission = 554/581 nm), with values normalized to total protein concentration (BCA assay, Thermo Fisher #23225) from the same lysates, expressed as tdTomato fluorescence intensity per μg protein.

**Patch clamping**

BMMSCs from Piezo1^flox/flox^ mice or PDGFRα-Piezo1 KO mice were plated on 35 mm non-coated plastic dishes, and Piezo1 currents were recorded from tdTomato-positive cells at a holding potential of -80 mV. Patch clamp recordings were performed in cell-attached configuration at room temperature. Data were acquired using an Axopatch 700A amplifier (Axon Instruments), Digidata 1322A digitizer (Molecular Devices) and pCLAMP10.7 software. Currents were sampled at 20 kHz and filtered at 1 kHz. Patch pipettes were made from 1.5 mm outer diameter glass (Vitrex Medical, #160214) using a Narishige PC-10 puller, and had resistance of 1.2 - 1.4 MΩ when filled with a saline solution consisting of 150 mM NaCl, 5 mM KCl, 2 mM CaCl_2_, 1 mM MgCl_2_, 10 mM HEPES (pH adjusted to 7.35 with NaOH). The bath solution consisted of 155 mM KCl, 3 mM MgCl_2_, 5 mM EGTA, 10 mM HEPES (pH adjusted to 7.2 with KOH). The osmolality of all solutions was 310 mOsmol.kg^-1^. Membrane patches were subjected to stepwise, 350-ms long pressure pulses (Δ: 10 mm Hg) through the recording electrode using a fast pressure-clamp device (High Speed Pressure Clamp-1 system, ALA-scientific). N indicates the number of mice, n is the number of recording from single cells.

**Isolation and differentiation of stromal vascular fractions (SVFs)**

Subcutaneous white adipose tissues (scWAT) from 8-week-old male PDGFRα-Piezo1 KO mice and their WT littermates were minced into pieces with sterile scissors before digesting in DMEM containing 2 mg/mL of Collagenase type I (Gibco, #17100017) and 3% of BSA (Sigma, #A7906) for 30 min at 37°C. The digested scWAT were then filtered through a 70 μm cell strainer (BD Biosciences) and centrifuged at 800 g for 10 min at 4°C to separate SVFs from the mature adipocyte fraction. The isolated SVFs were then resuspended with DMEM and plated on a 10 cm cell culture dish. After reaching 90% confluency, cells were sub-cultured and seeded in a 24-well plate (5×10^4^ cells per well). When the cells reached 100% confluency, adipogenic induction medium containing 1 μM dexamethasone, 0.5 mM IBMX, 1 μM rosiglitazone, and 1.8 μM insulin was added to each well. After 48 hours, the medium was changed to a maintenance medium containing 1 μM rosiglitazone, and 1.8 μM insulin. The maintenance medium was replaced every 2 days until day 8.^2^

**Western blot analysis**

Proteins were extracted from BMMSCs using radioimmunoprecipitation assay (RIPA) Lysis Buffer (Beyotime Biotechnology, #P0013B) supplemented with protease inhibitor cocktail (Beyotime Biotechnology, #P1005). Protein lysates were separated by SDS-PAGE, transferred onto 0.22 μm polyvinylidene difluoride (PVDF) membranes (Vazyme, #E802-01), and probed with primary antibodies against Klf2 (rabbit polyclonal, Proteintech #23384-1-AP, 1:1000) and Hsp90 (rabbit mAb, Cell Signaling Technology, #4874S, 1:2500), followed by incubation with HRP-conjugated anti-rabbit secondary antibodies (Cell Signaling Technology, #7074). Protein bands were visualized using SuperFemto ECL Chemiluminescence Kit (Vazyme, #E423-01) and quantified with ImageJ software (v1.53), with Klf2 expression normalized to Hsp90. Uncropped blot scans are provided in Supplementary Information.

**Senescence-associated β-galactosidase (SA-β-gal) activity assay**

WT and KO BMMSCs were lysed, and the lysates were incubated with the substrate o-Nitrophenyl-β-D-galactopyranoside (ONPG) in sodium phosphate buffer (pH 6.0) at 37°C. SA-β-gal activity was quantified using the Lactase/β-Galactosidase Activity Assay Kit (SolarBio, #BC2580) according to manufacturer's instructions. Absorbance was measured at 400 nm using the CLARIOstar microplate reader (BMG LABTECH, #0430-101). Enzyme activity was calculated based on the standard curve generated with p-nitrophenol standards and normalized to total protein concentration.

**References:**

1 Prins, H. J. *et al.* In vitro induction of alkaline phosphatase levels predicts in vivo bone forming capacity of human bone marrow stromal cells. *Stem Cell Res.* **12**, 428-440 (2014).

2 Liu, L. *et al.* Isolation of Mouse Stromal Vascular Cells for Monolayer Culture. *Methods Mol. Biol.* **1566**, 9-16 (2017).


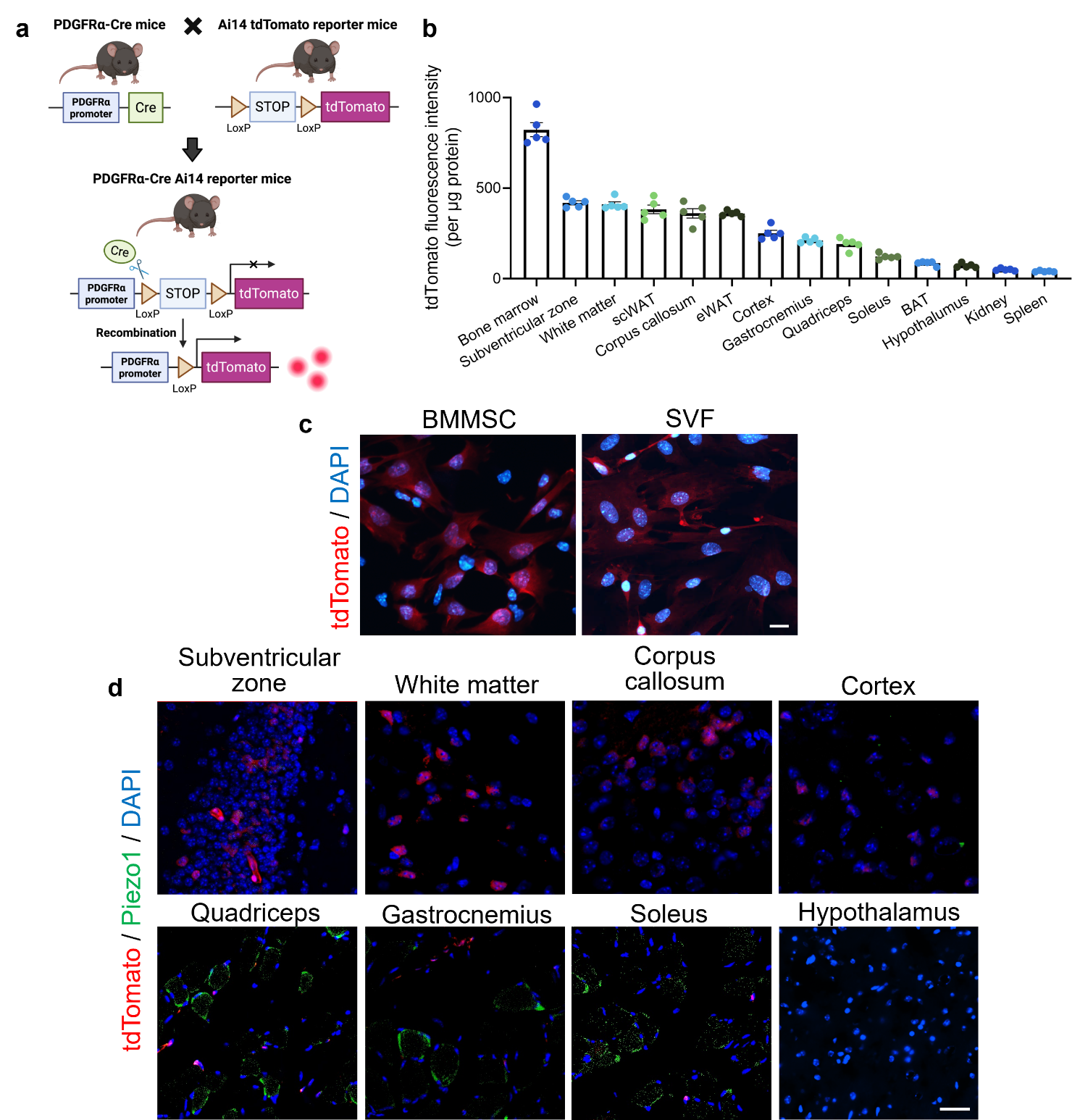


**Figure S1. Related to Figure 1. Visualization and quantification of tdTomato expression in PDGFRα-Cre tdTomato reporter mice. (a-d)** PDGFRα-Cre mice were crossed with tdTomato fluorescent reporter (Ai14) mice in C57BL/6J background to generate PDGFRα-Cre tdTomato reporter mice under the control of the PDGFRα promoter (abbreviated as PDGFRα-Cre Ai14). **(a)** Schematic diagram showing the generation of PDGFRα-Cre Ai14 mice; created with BioRender (https://BioRender.com). **(b)** Quantification of tdTomato fluorescence intensity in different tissues of 8-week-old male PDGFRα-Cre Ai14 mice. The fluorescence intensity in each tissue homogenate was quantified with a BMG CLARIOstar Plus microplate reader and values are normalized to total protein concentration. scWAT, subcutaneous white adipose tissue; eWAT, epididymal white adipose tissue; BAT, brown adipose tissue. **(c)** Fluorescence microscopy images showing tdTomato expression (red) in BMMSCs and SVFs isolated from 8-week-old male PDGFRα-Cre Ai14 mice. The nuclei were counterstained with DAPI (blue). Scale bar, 25 μm. **(d)** Immunofluorescence staining showing expression and localization of Piezo1 (green) in tissue sections of different brain areas and skeletal muscles from 8-week-old male PDGFRα-Cre Ai14 mice. The nuclei were counterstained with DAPI (blue). Red fluorescence represents tdTomato-expressing cells in the PDGFRα-Cre Ai14 mice. Scale bar, 100 μm. Note that no co-localization was detected between Piezo1 (green) and tdTomato (red) in different types of skeletal muscle. The Piezo1 expression was virtually undetectable in subventricular zone, white matter, corpus callosum, cortex, and hypothalamus. *n*=5 for each group. The data are presented as the means ± SEMs.


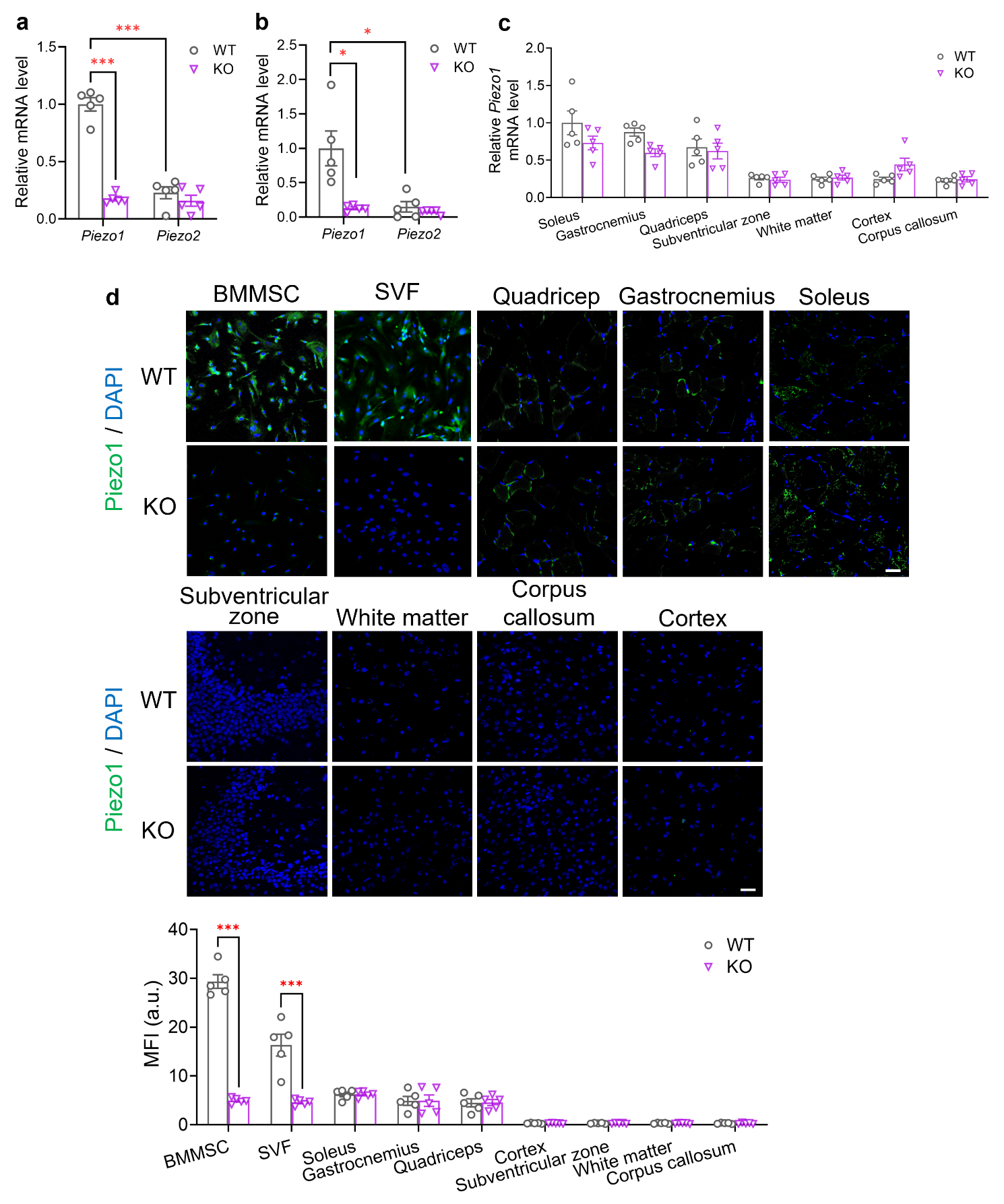


**Figure S2. Related to Figure 1. Piezo1 ablation in PDGFRα-Piezo1 KO mice occurs predominantly in BMMSCs and SVFs. (a-b)** The mRNA levels of *Piezo1* and *Piezo2* in BMMSCs **(a)** and scWAT-derived SVFs **(b)** isolated from 8-week-old PDGFRα-Piezo1 KO mice and wild-type (WT) littermates, determined by real-time PCR analysis. **(c)** The mRNA levels of *Piezo1* in BMMSCs, scWAT-derived SVFs, different types of skeletal muscle (soleus, gastrocnemius, quadriceps), and different brain areas (subventricular zone, white matter, corpus callosum, cortex, hypothalamus). **(d)** Representative images showing immunofluorescence staining of Piezo1 (green) in BMMSCs, SVFs, different types of skeletal muscle, and different brain areas. Nuclei were stained with DAPI (blue). Scale bar, 50 μm. The lower panel is the quantification of the mean fluorescence intensity (MFI) of Piezo1. a.u., arbitrary unit. *n*=5 for each group. The data are presented as the means ± SEMs, **p* < 0.05, ****p* < 0.001.


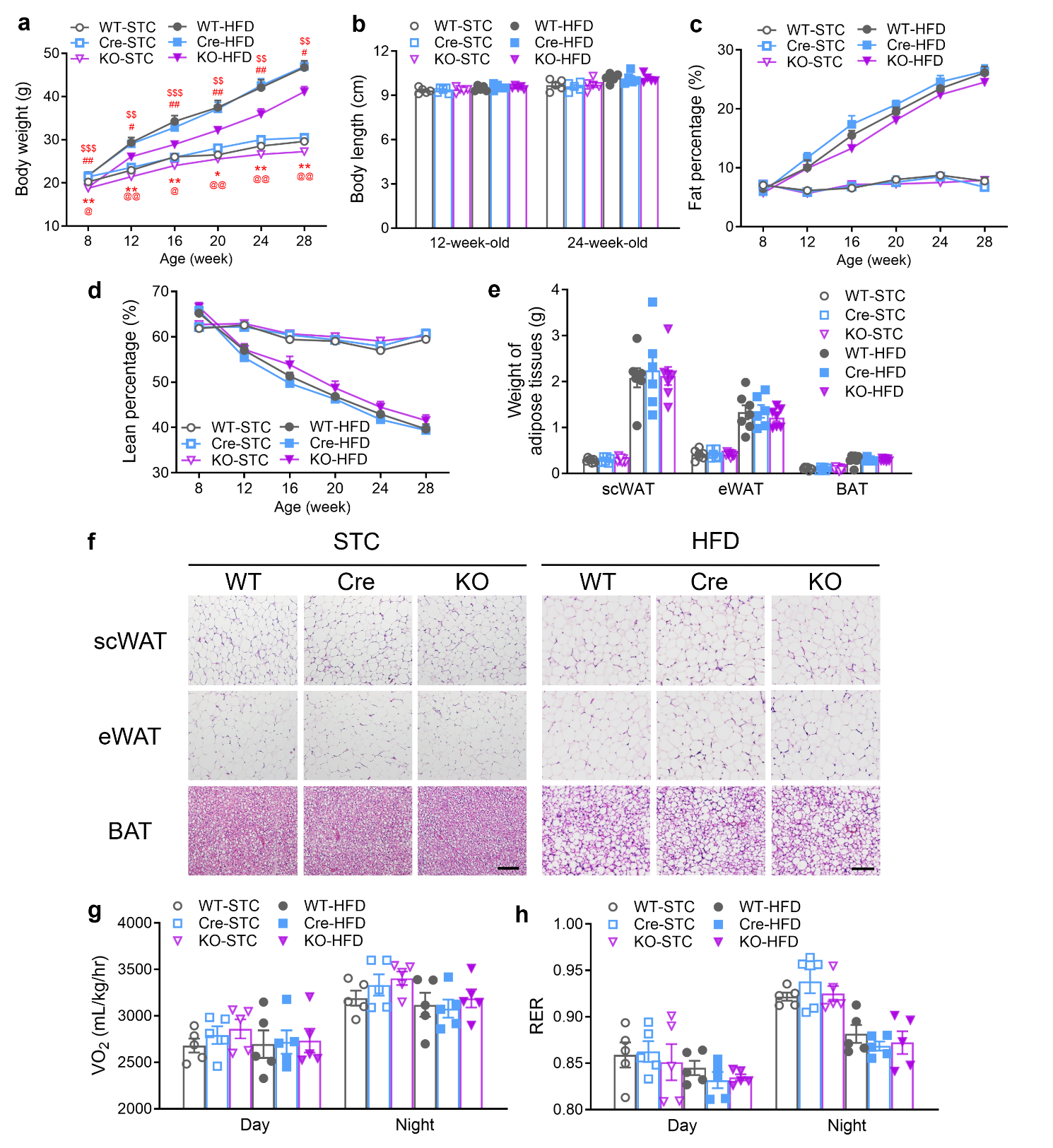


**Figure S3. Related to Figure 1. Metabolic characterization of PDGFRα-Piezo1 KO mice.** Eight-week-old male Piezo1^flox/flox^ mice (WT), PDGFRα-Cre mice, and PDGFRα-Piezo1 KO mice were fed with standard chow diet (STC) or 45% high-fat diet (HFD) for 20 weeks. **(a)** Body weight of male PDGFRα-Piezo1 KO mice, Piezo1^flox/flox^ and PDGFRα-Cre mice. *n*=6-8. **(b)** Body length was measured at the age of 12 weeks and 24 weeks, respectively. *n*=5. **(c-d)** Percentage of fat **(c)** and lean mass **(d)** were quantified by a nuclear magnetic resonance (NMR) body composition analyzer. *n*=6-8. **(e)** Wet weight of scWAT, eWAT, and BAT at week-28. *n*=6-8. **(f)** Representative images of H&E staining of scWAT, eWAT, and BAT in mice on STC or HFD feeding. Scale bar, 100 μm. **(g-h)** Oxygen consumption **(g)** and respiratory exchange ratio (RER) **(h)** were measured using metabolic chambers when the mice were 16-week-old. *n*=5. The data are presented as the means ± SEMs, **p* < 0.05, ***p* < 0.01 (WT-STC vs KO-STC); ^@^*p* < 0.05, ^@@^*p* < 0.01 (KO-STC vs Cre-STC); ^#^*p* < 0.05, ^##^*p* < 0.01 (WT-HFD vs KO-HFD); ^$$^*p* < 0.01, ^$$$^*p* < 0.001 (KO-HFD vs Cre-HFD).


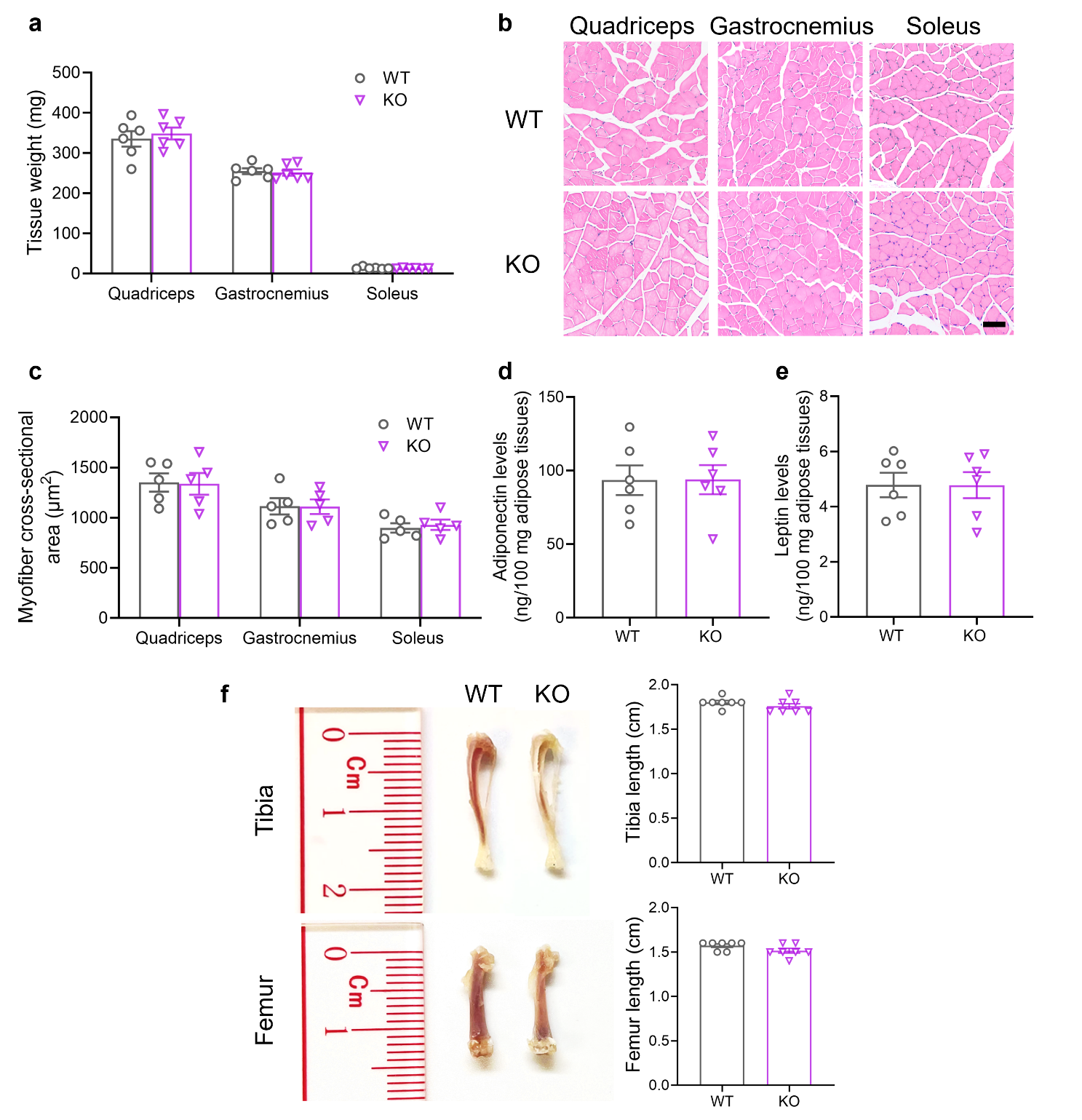


**Figure S4. Related to Figure 1. No obvious change in weight and morphology of skeletal muscle, secretion of adipokines, and skeleton development in PDGFRα-Piezo1 KO mice. (a)** Wet weight of quadriceps, gastrocnemius, and soleus muscles of 14-week-old male PDGFRα-Piezo1 KO mice and WT littermates. *n*=6. **(b)** Representative images of H&E staining of different types of skeletal muscle. Scale bar, 50 μm. **(c)** Quantification of average myofiber cross-sectional area in H&E-stained images. **(d-e)** Levels of adiponectin **(d)** and leptin **(e)** in the serum-free conditioned medium of subcutaneous adipose tissue explants measured by ELISA, after 24 hours of explant culture. *n*=6. **(f)** Representative images of femurs and tibias (left panel) and qualifications of bone length (right panel). *n*=7. The data are presented as the means ± SEMs.


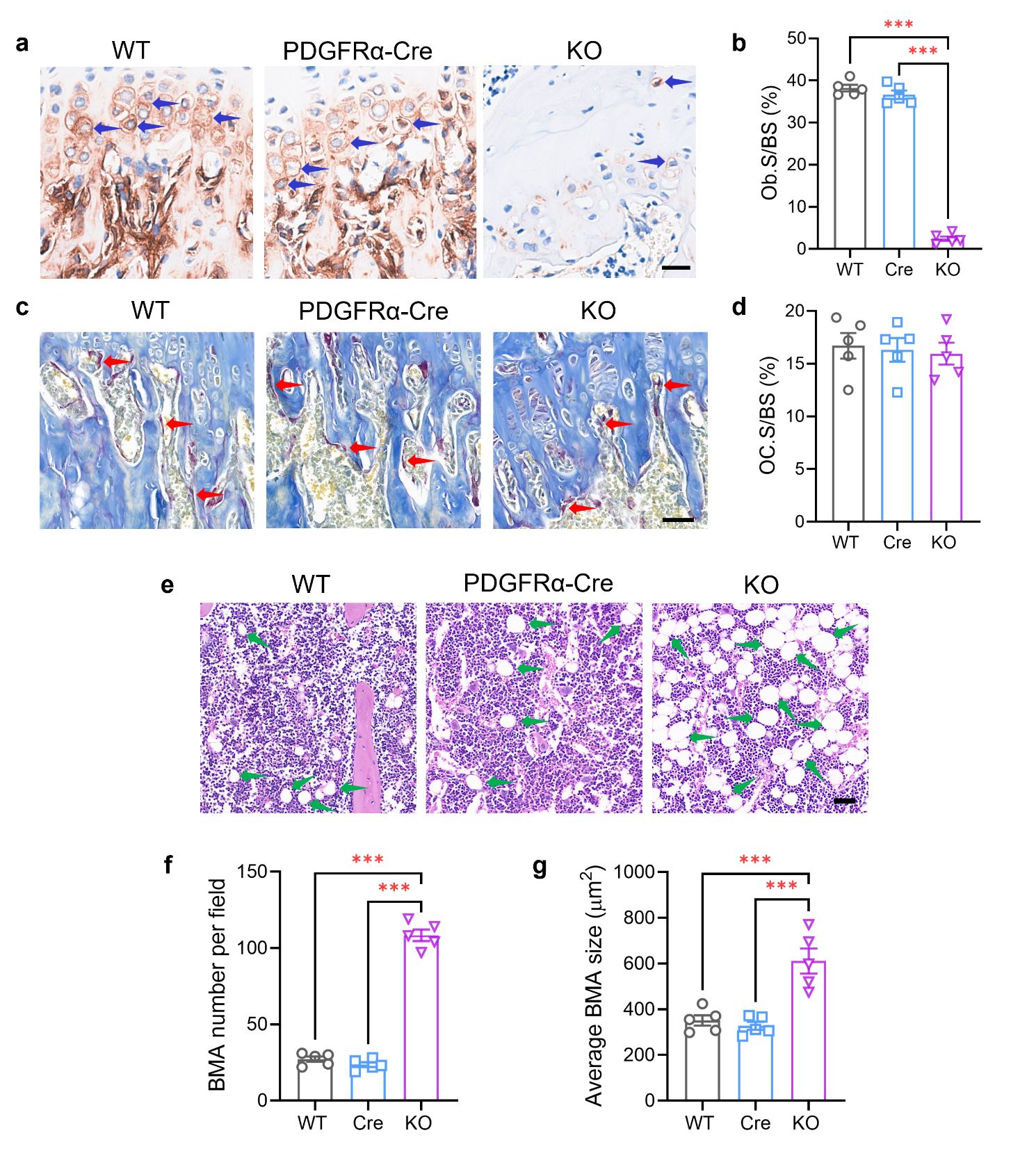


**Figure S5. Related to Figure 1. Histological analyses of osteoblasts, osteoclasts and bone marrow adipocytes (BMAs) in PDGFRα-Piezo1 KO mice.** Tibias dissected from 18-week-old male PDGFRα-Piezo1 KO mice, WT littermates, and PDGFRα-Cre mice were decalcified, paraffinized, sectioned and subjected to staining for alkaline phosphatase (ALP), tartrate-resistant acid phosphatase (TRAP) or H&E staining to visualize osteoblasts, osteoclasts and adipocytes, respectively. **(a)** Representative images showing immunohistochemical staining of ALP in paraffin-embedded decalcified tibias. Blue arrows indicate the ALP-stained osteoblasts. Scale bar, 25 μm. **(b)** Quantification of osteoblast surface as a percentage of bone surface (Ob.S/BS, %). *n*=5. **(c)** Representative images showing TRAP staining of paraffin-embedded decalcified tibias. Red arrows indicate the TRAP-stained osteoclasts. Scale bar, 25 μm. **(d)** Quantification of osteoclast surface normalized by bone surface (Oc.S/BS, %). *n*=5. **(e)** Representative images showing H&E staining of paraffin-embedded decalcified tibias. Green arrows indicate bone marrow adipocytes. Scale bar, 50 μm. **(f-g)** Quantification of BMA number per field **(f)** and average BMA size (μm^2^) **(g)**. *n*=5 for each group. The data are presented as the means ± SEMs, ****p* < 0.001.


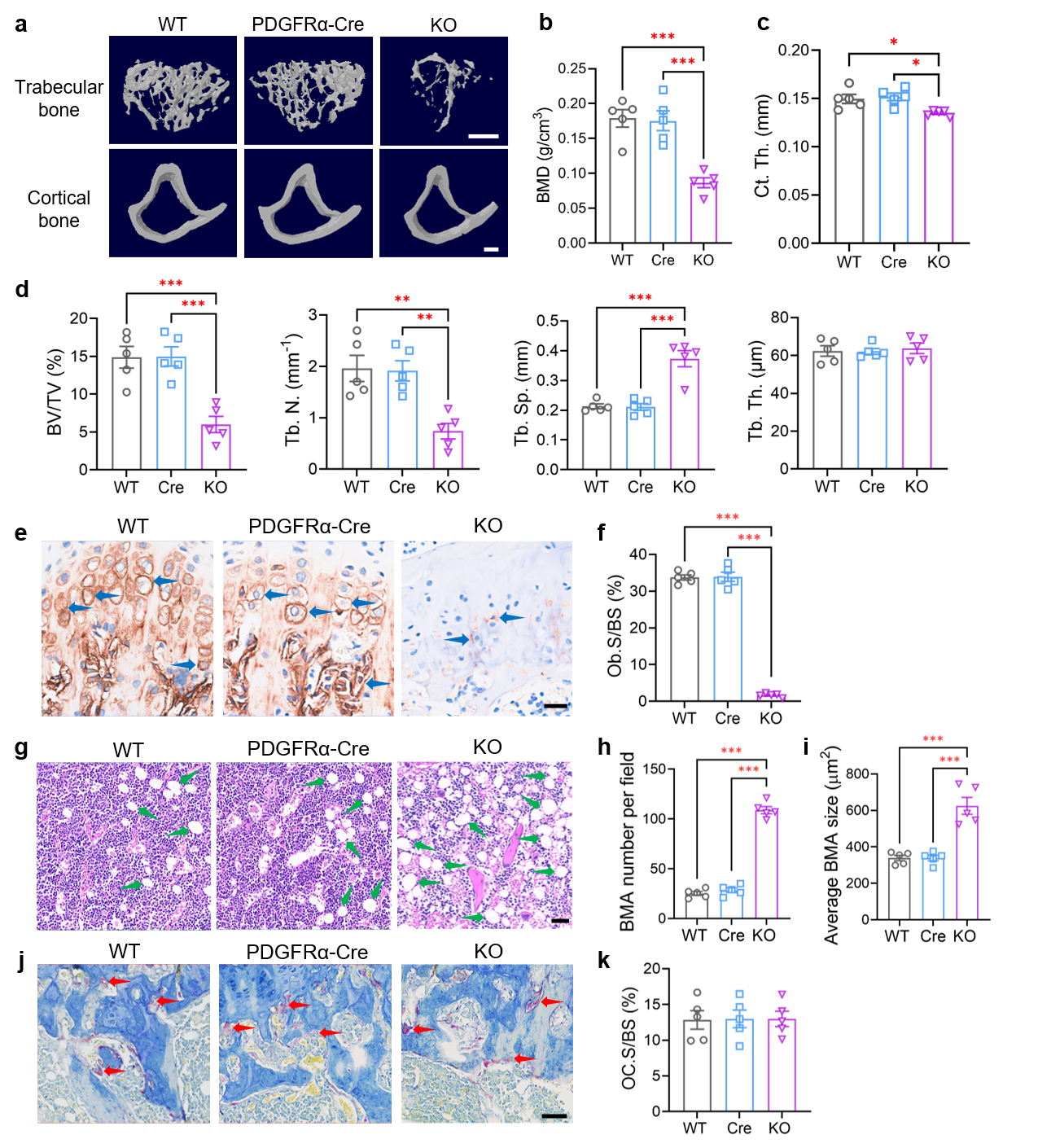


**Figure S6. Related to Figure 1. Female PDGFRα-Piezo1 KO mice also display osteoporosis and bone marrow adiposity.** **(a)** Representative micro-CT images showing three-dimensional trabecular architecture and cortical bone at the proximal tibia of 18-week-old female PDGFRα-Piezo1 KO mice, WT littermates, and PDGFRα-Cre mice. Scale bar, 500 μm. **(b-d)** Micro-CT measurements of bone mineral density (BMD, **b**), cortical thickness (Ct. Th., **c**), bone volume fraction (BV/TV, **d**), trabecular number (Tb. N., **d**), trabecular separation (Tb. Sp., **d**), and trabecular thickness (Tb. Th., **d**) at the proximal tibia. **(e)** Immunohistochemical staining of ALP in paraffin-embedded decalcified tibias. Blue arrows indicate the ALP-stained osteoblasts. Scale bar, 25 μm. **(f)** Quantification of osteoblast surface as a percentage of bone surface (Ob.S/BS, %). *n*=5. **(g)** H&E staining of paraffin-embedded decalcified tibias. Green arrows indicate bone marrow adipocytes. Scale bar, 50 μm. **(h-i)** Quantification of BMA number per field **(h)** and average BMA size (μm^2^) **(i)**. **(j-k)** Representative images **(j)** and quantification **(k)** of TRAP staining. Red arrows indicate the TRAP-stained osteoclasts. Scale bar, 25 μm. *n*=5 for each group. The data are presented as the means ± SEMs, **p* < 0.05, ***p* < 0.01, ****p* < 0.001.


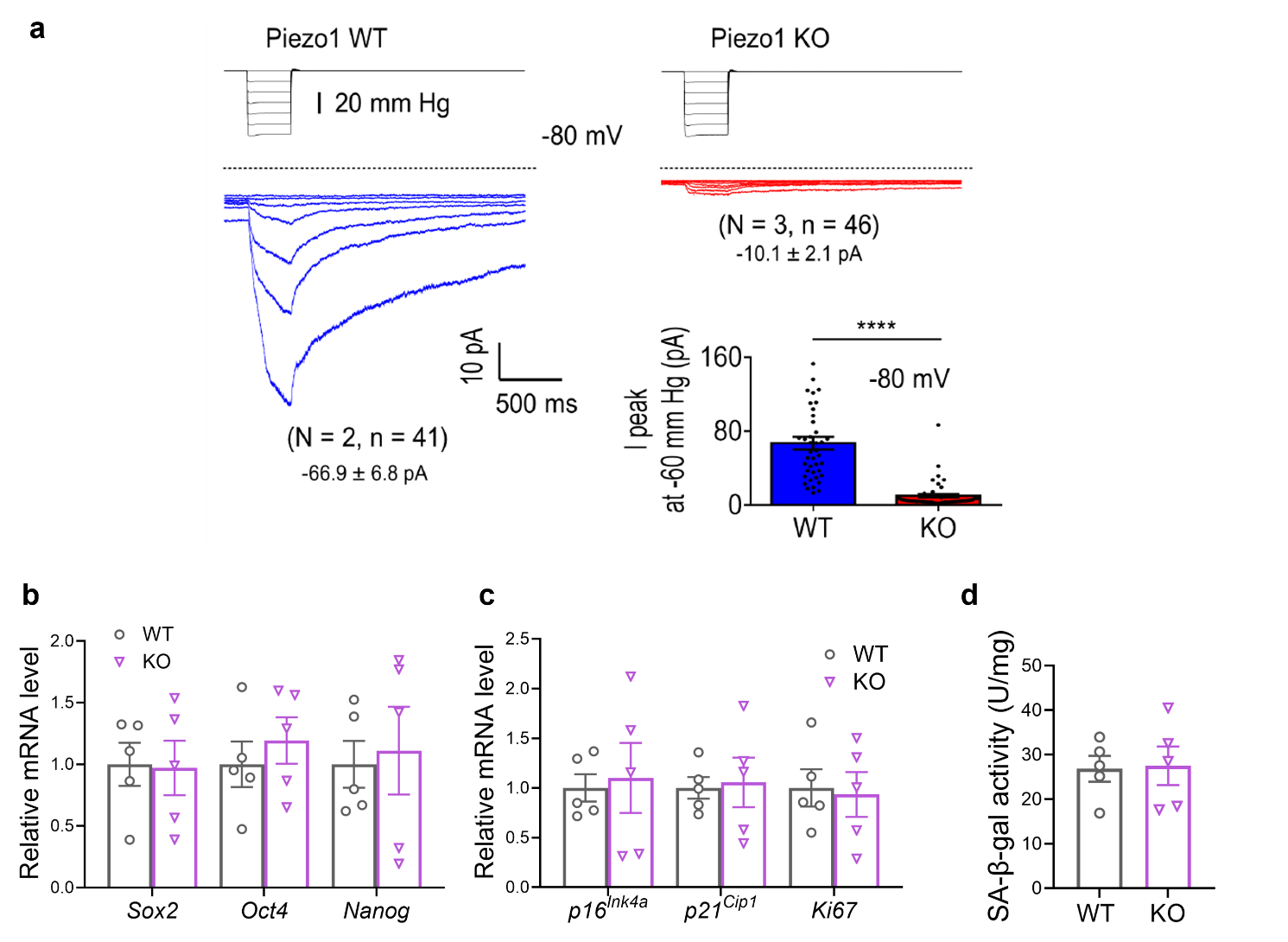


**Figure S7. Related to Figure 2. Piezo1 ablation in BMMSCs abolishes Piezo1 channel activity, but has no effect on multipotency, senescence, or proliferative capacity. (a)** Piezo1 currents in tdTomato-positive WT and KO BMMSCs recorded in the cell-attached configuration. The inset histogram shows peak current amplitude elicited by a pressure pulse of -60 mm Hg. N indicates the number of mice, while n is the number of recording from single cells. **(b-c)** Relative mRNA expression levels of several key genes related to multipotency (*Sox2, Oct4, Nanog*), senescence (*p16^Ink4a^, p21^Cip1^*), and proliferation (*Ki67*) in WT and Piezo1 KO BMMSCs. **(d)** Quantitative analysis of enzymatic activity of senescence-associated β-galactosidase (SA-β-gal) using Lactase/β-Galactosidase Activity Assay Kit (SolarBio, #BC2580). Enzymatic activity was normalized to total cell protein concentration. *n*=5. The data are presented as the means ± SEMs, *****p* < 0.0001.


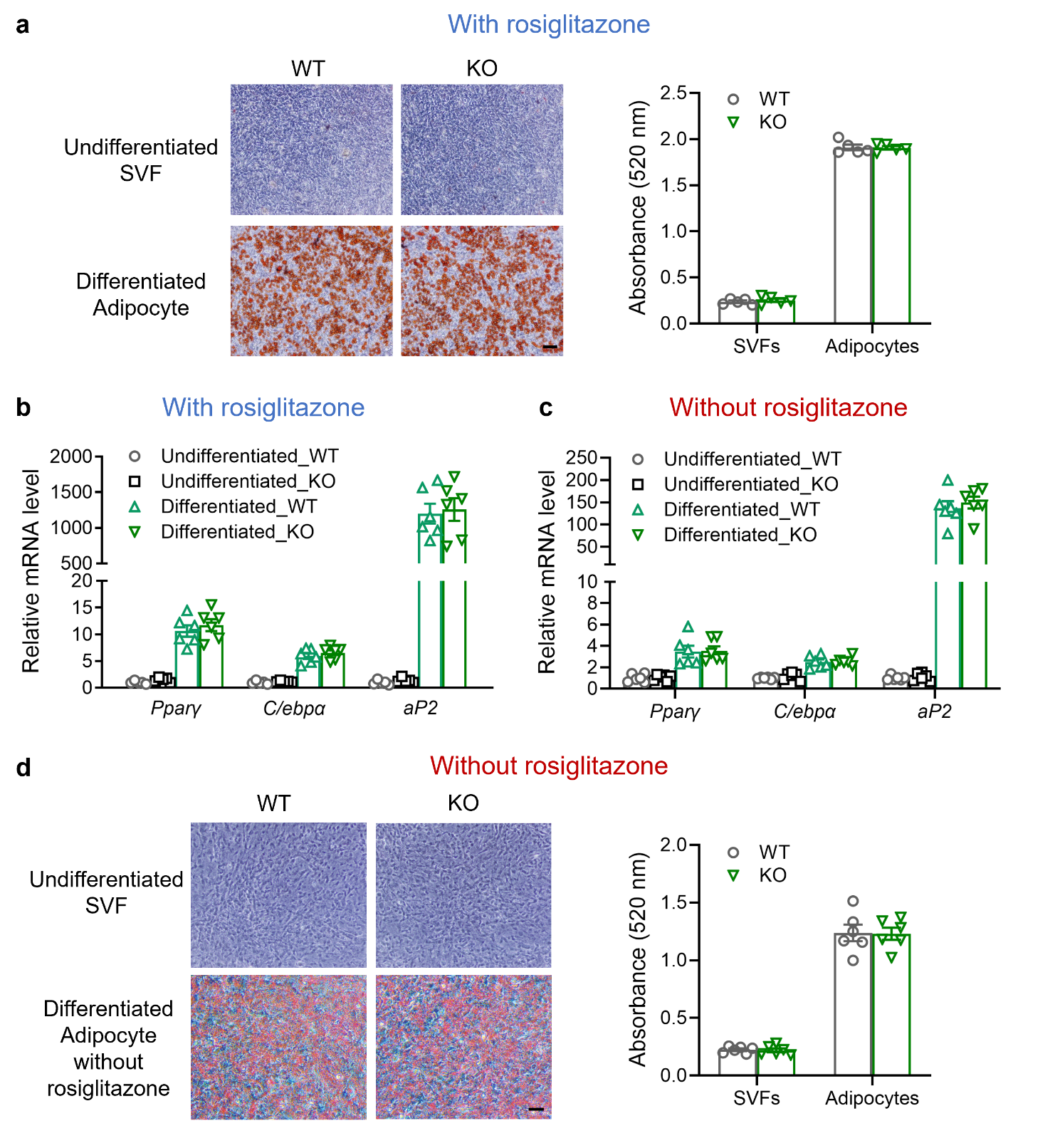


**Figure S8. Related to Figure 2. Piezo1 ablation does not affect adipogenesis of stromal vascular fractions isolated from scWAT.** Stromal vascular fractions (SVFs) were isolated from 8-week-old PDGFRα-Piezo1 KO mice and their WT littermates, followed by *in vitro* adipocyte differentiation by adding 1 μM dexamethasone, 0.5 mM IBMX, 1 μM rosiglitazone, and 1.8 μM insulin into the culture medium. **(a)** Representative images of Oil Red O staining. The right panel shows the quantitative analysis of Oil Red O staining by determining OD520 absorbance values. *n*=5. Scale bar, 100 μm. **(b)** Real-time PCR analysis for mRNA abundance of several adipogenic markers *(aP2*, *Pparγ*, *C/ebpα*) in cells at day 8 after differentiation or without differentiation induction. *n*=5-6. **(c-d)** SVFs were isolated from 8-week-old PDGFRα-Piezo1 KO mice and their WT littermates, followed by *in vitro* adipocyte differentiation as in panel **a** without adding rosiglitazone into the culture medium. **(c)** Real-time PCR analysis of adipogenic genes. **(d)** Representative images of Oil Red O staining. The right panel shows the quantitative analysis of Oil Red O staining by determining OD520 absorbance values. *n*=6. Scale bar, 100 μm. The data are presented as the means ± SEMs.


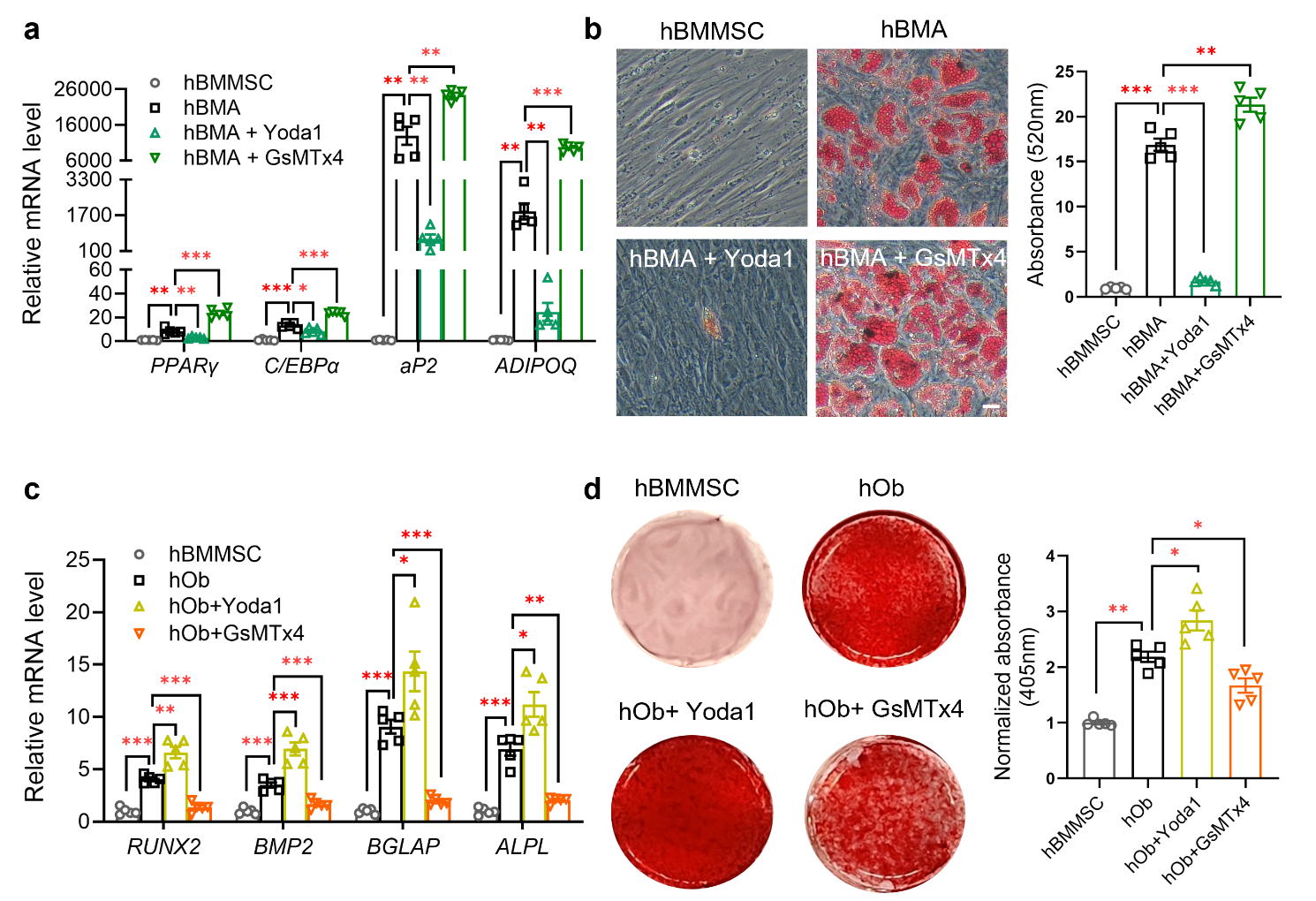


**Figure S9. Related to Figure 2. Effects of pharmacological activation/inhibition of Piezo1 on adipogenesis and osteogenesis in human BMMSCs. (a-e)** hBMMSCs were pre-treated with Piezo1 activator Yoda1 (5 μM) or Piezo1 inhibitor GsMTx4 (5 μM), and then treated with the adipogenic cocktail containing 1 μM dexamethasone, 0.5 mM IBMX, 200 μM indomethacin, and 1.8 μM insulin **(a, b)**, or the osteogenic cocktail containing 100 nM dexamethasone, 50 ug/mL ascorbic acid, and 10 mM β-glycerophosphate **(c, d)**. **(a)** qPCR analysis for mRNA expression levels of several adipogenic genes in cells at 14 days after adipogenic differentiation. hBMMSCs without addition of the adipogenic cocktail were used as controls. **(b)** Representative images of Oil Red O staining. Scale bar, 100 μm. The right panel is quantitative analysis by determining OD520 absorbance values. **(c)** qPCR analysis for mRNA abundance of the osteogenic genes in cells at 21 days after addition of the osteogenic cocktail. **(d)** Representative images of Alizarin Red S staining. The right panel is quantitative analysis by determining OD405 absorbance values. The data is expressed as fold changes to hBMMSCs without differentiation. *n*=5 for each group. The data are presented as the means ± SEMs, **p* < 0.05, ***p* < 0.01, ****p* < 0.001.


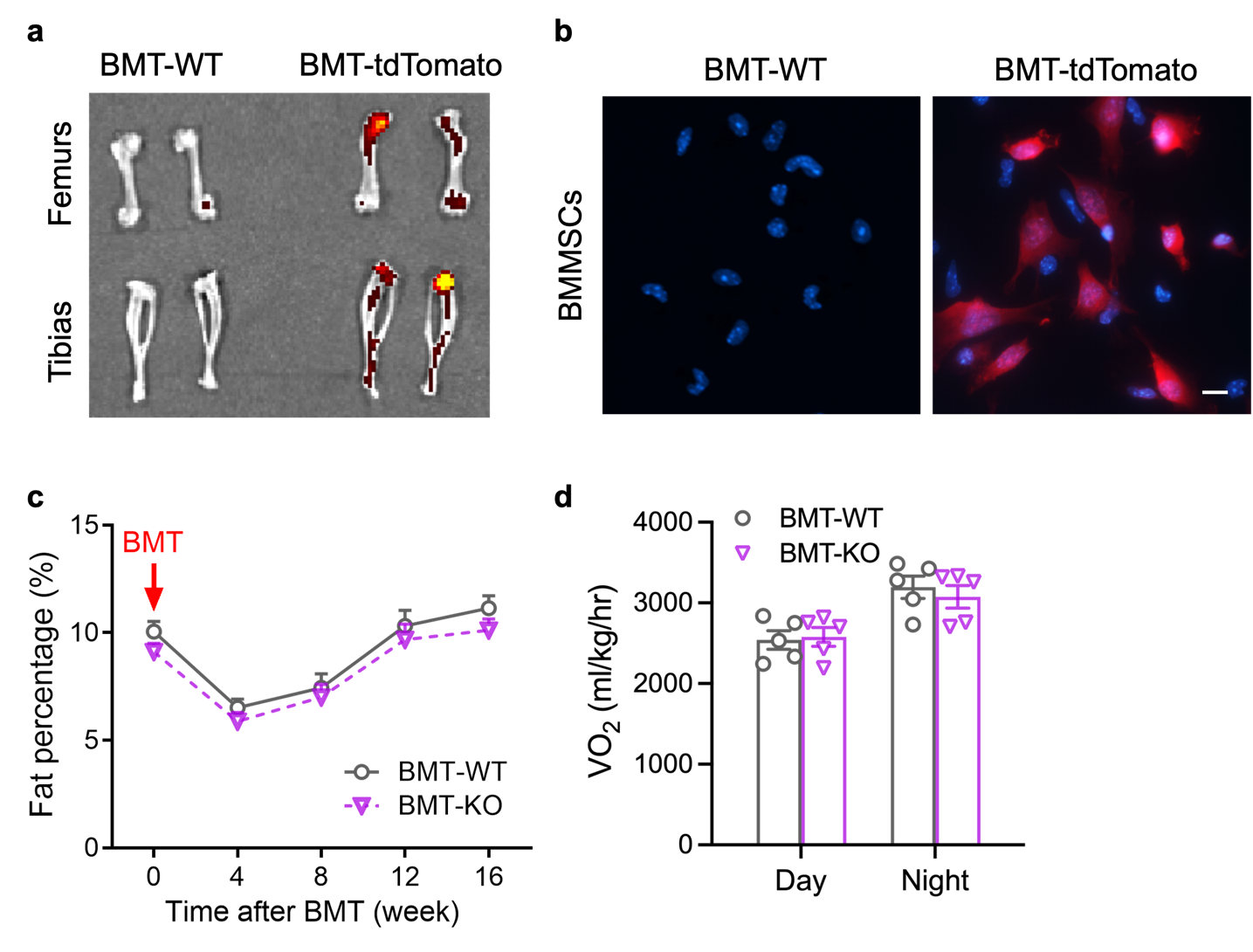


**Figure S10. Related to Figure 3. Confirmation of successful engraftment of BMMSCs after bone marrow transplantation (BMT).** Twelve-week-old C57BL/6J recipient mice received 9 Gy total body irradiation to deplete bone marrow. In the following day, a total of 1 x 10^7^ bone marrow cells isolated from femurs and tibias of 6-week-old male PDGFRα-Cre Ai14 reporter mice or PDGFRα-Piezo1 KO mice and their WT littermates, were intravenously injected into a recipient mouse via a tail vein. **(a)** IVIS imaging showing tdTomato signal in femurs and tibias of C57BL/6J recipient mice receiving BMT from PDGFRα-Cre tdTomato reporter mice (BMT-tdTomato) or C57BL/6J WT mice (BMT-WT) for 10 weeks. **(b)** Representative microscopic images showing tdTomato fluorescence (red) in BMMSCs isolated from BMT-tdTomato or BMT-WT mice for 10 weeks. Nuclei were stained with DAPI (blue). Scale bar, 25 μm. **(c)** Percentage of total fat mass before and at different days after BMT. **(d)** Oxygen consumption measured by metabolic chambers, at 12 weeks after BMT. *n*=5 for each group. The data are presented as the means ± SEMs.


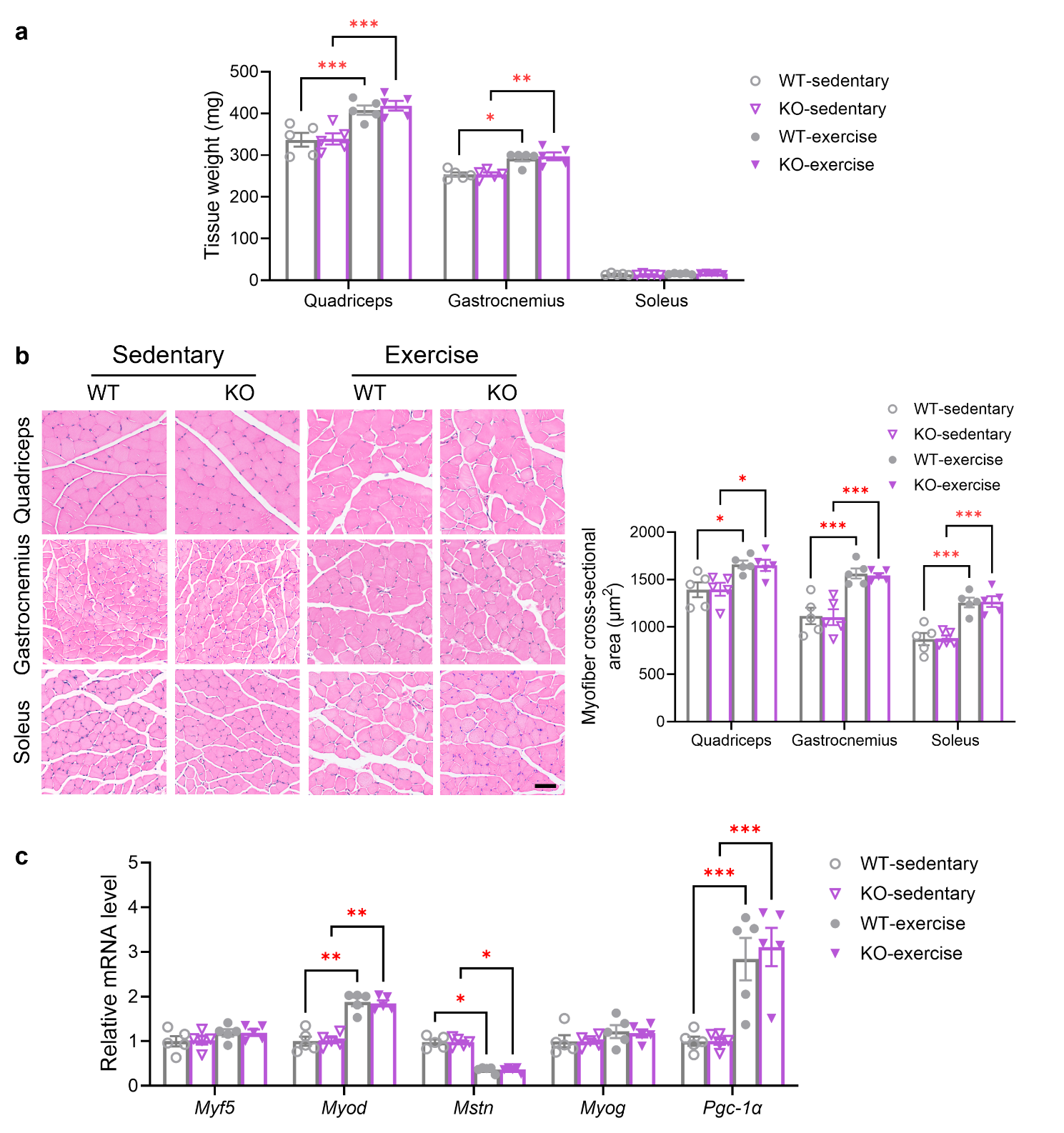


**Figure S11. Related to Figure 4. No obvious change in weight, morphology and development/metabolism-related gene expression in skeletal muscle of PDGFRα-Piezo1 KO mice after treadmill exercise.** Eight-week-old male PDGFRα-Piezo1 KO mice and WT littermates were subjected to treadmill exercise or remained sedentary for 6 weeks as in Figure 4. **(a)** Wet weight of quadriceps, gastrocnemius, and soleus muscles. **(b)** Representative images of H&E staining of different types of skeletal muscle. Scale bar, 50 μm. The right panel is the quantification of average myofiber cross-sectional area in H&E-stained images. **(c)** qPCR analysis of development- and metabolism-related genes (Myogenic factor 5 [*Myf5*], Myoblast determination protein 1 [*Myod*], Myostatin [*Mstn*], Myogenin [*Myog*], Peroxisome proliferator-activated receptor gamma coactivator 1-alpha [*Pgc-1α*]) in gastrocnemius muscle. *n*=5 for each group. The data are presented as the means ± SEMs, **p* < 0.05, ***p* < 0.01, ****p* < 0.001.


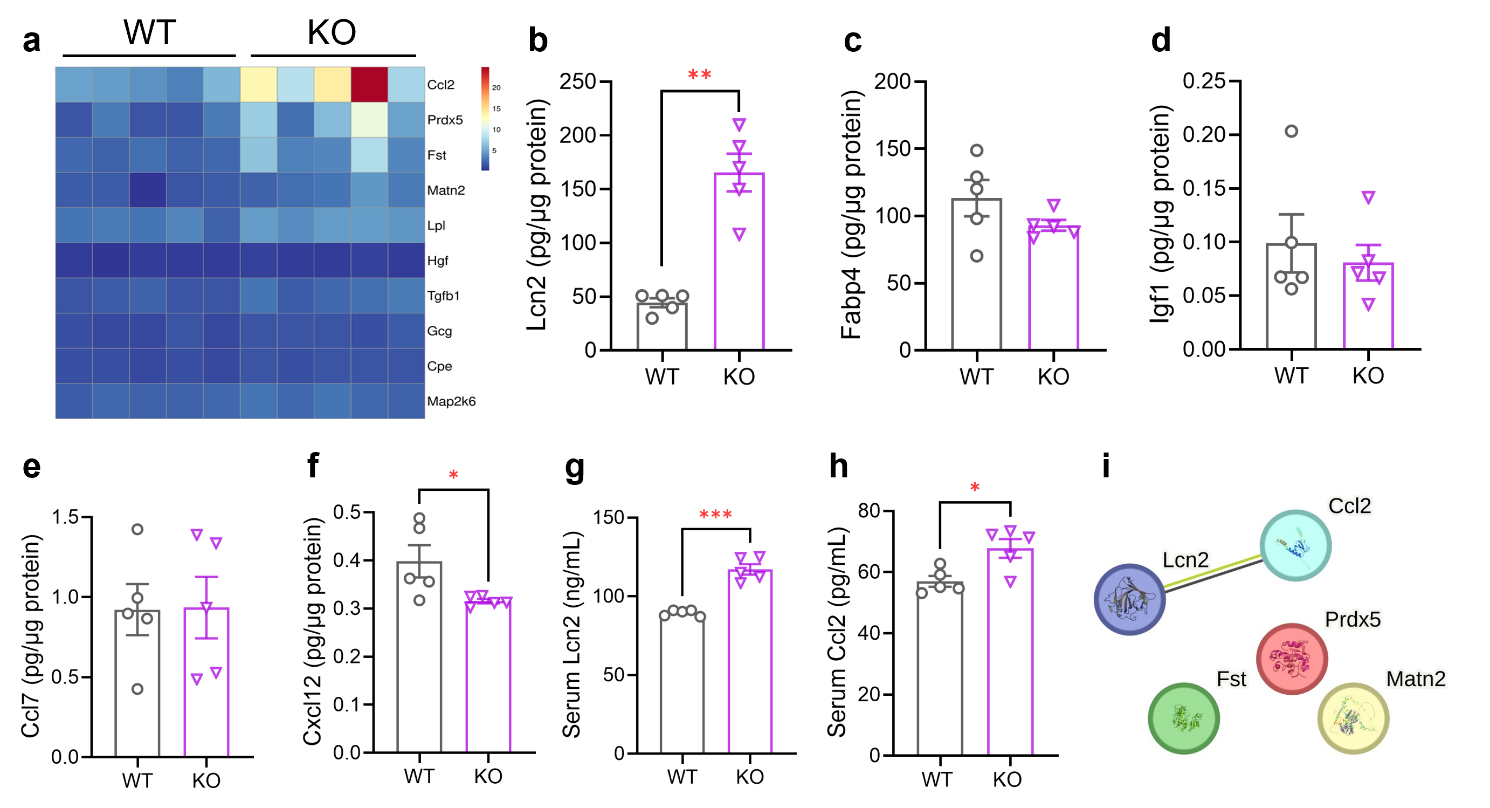


**Figure S12. Related to Figure 5. Identification of Piezo1-dependent secretory factors.** Serum-free culture media of WT and KO BMMSCs were collected and subjected to Olink, multiplex assay, and ELISA analyses. **(a)** Olink proteomics analysis was conducted using Olink Mouse Exploratory panel. Heatmap showed linearized normalized protein expression (linear NPX) of proteins with significant changes in KO BMMSCs. **(b-c)** ELISA analyses of Lcn2 **(b)** and Fabp4 **(c**). **(d-f)** Luminex® multiplex assay of Igf1, Ccl7 and Cxcl12. **(g-h)** Circulating levels of Lcn2 **(g)** and Ccl2 **(h)** determined by ELISA. **(i)** Correlation analysis of the top five differential proteins in the protein-protein interaction (PPI) network using STRING (https://string-db.org/). *n*=5 for each group. The data are presented as the means ± SEMs, **p* < 0.05, ***p* < 0.01, ****p* < 0.001.


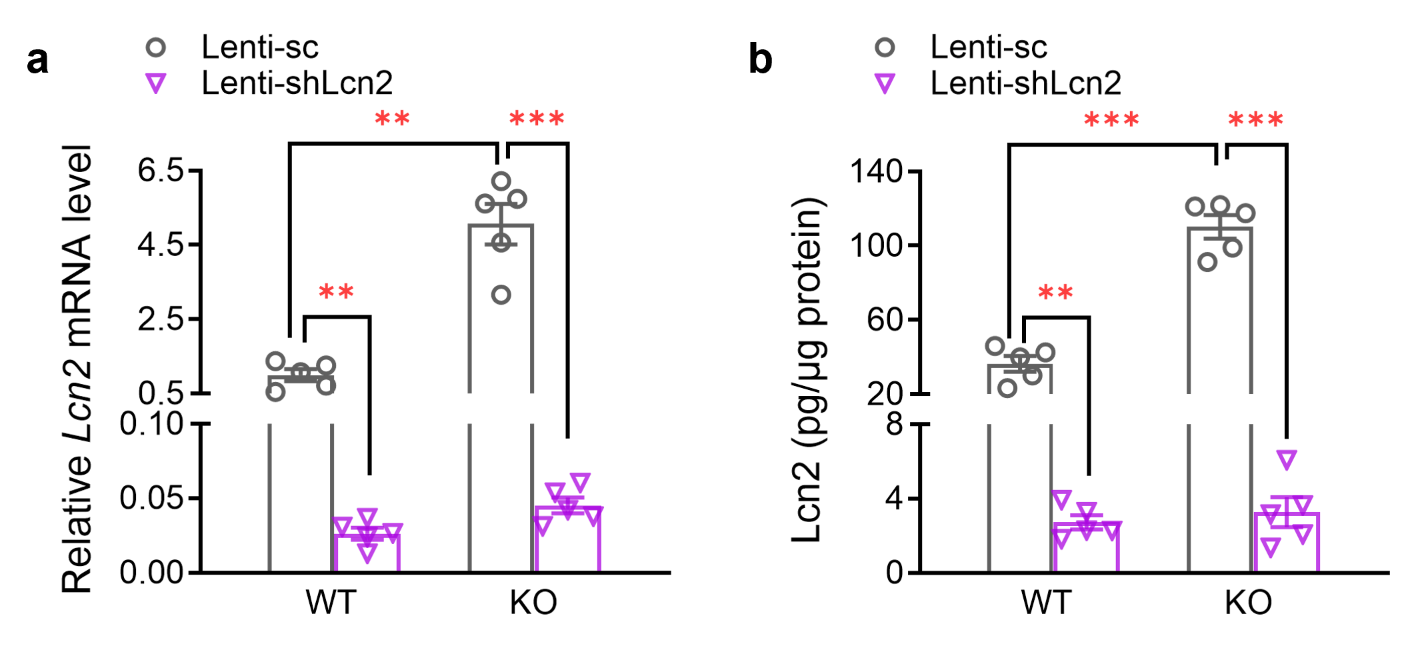


**Figure S13. Related to Figure 6. Validation of efficacy of lentivirus-mediated knockdown of lipocalin-2 in BMMSCs. (a-b)** BMMSCs isolated from PDGFRα-Piezo1 KO mice or WT littermates were infected with lentivirus (MOI=40) encoding *eGFP* together scrambled shRNA or shRNA against *Lcn2* (shLcn2) for 72 hours with the presence of 5 μg/mL polybrene. **(a)** qPCR analysis for *Lcn2* mRNA level in Piezo1 KO and WT BMMSCs after 72-hour transfection. **(b)** ELISA analysis of Lcn2 protein level in culture medium. *n*=5 for each group. The data are presented as the means ± SEMs, ***p* < 0.01, ****p* < 0.001.


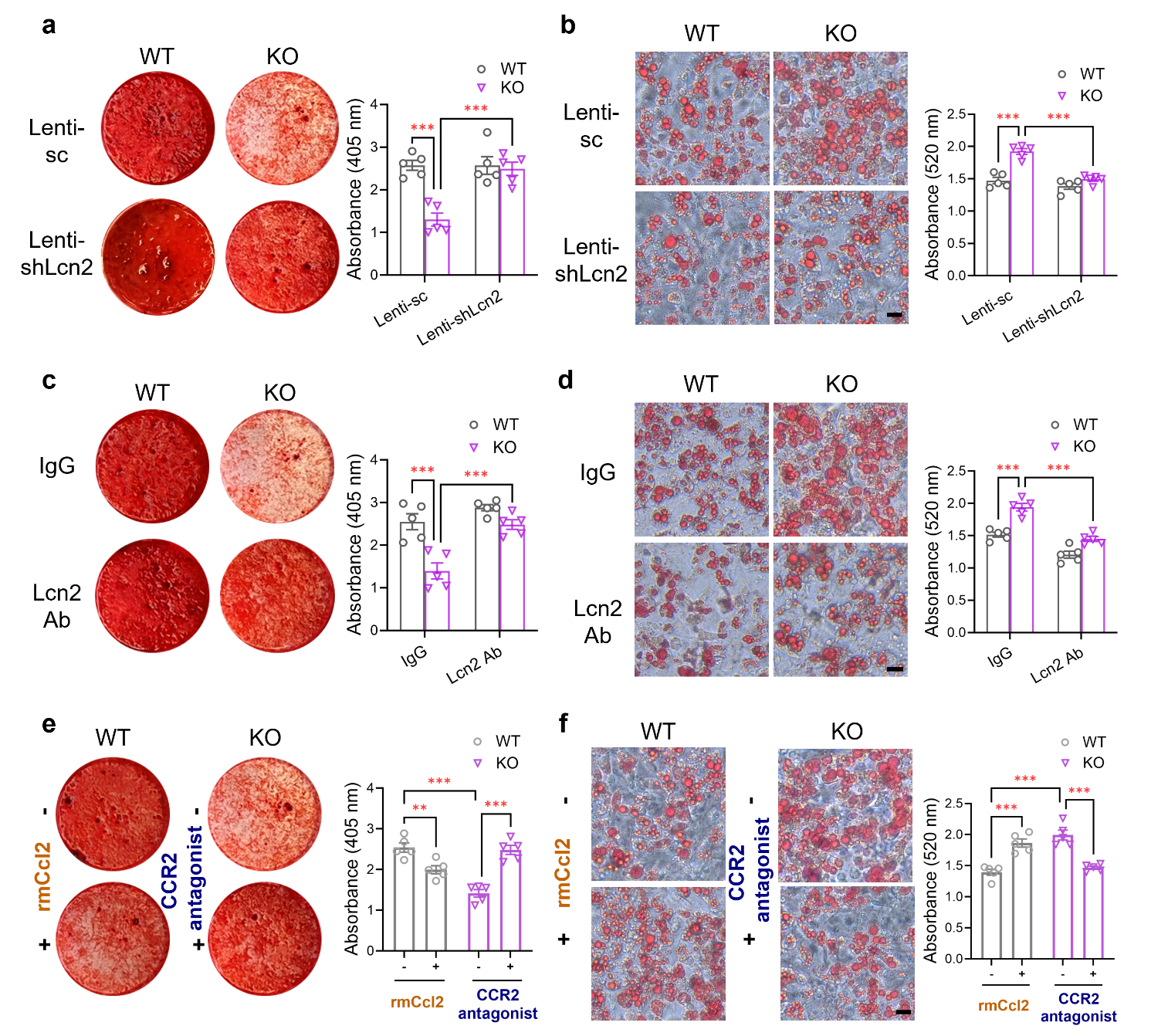


**Figure S14. Related to Figure 6 and Figure 7. Blockage of Lcn2 or Ccl2 reverses Piezo1 deficiency-induced alterations in adipogenesis and osteogenesis of BMMSCs.** BMMSCs were isolated from femurs and tibias of 10-week-old male PDGFRα-Piezo1 KO mice and WT littermates for differentiation into osteoblasts or adipocytes. **(a-b)** BMMSCs were infected with lentivirus (MOI=40) encoding *eGFP* together with scrambled shRNA (Lenti-sc) or shRNA against *Lcn2* (Lenti-shLcn2) for 72 hours with the presence of 5 μg/mL polybrene before differentiation into osteoblasts or adipocytes. Alizarin Red S staining for osteoblasts **(a)** and Oil Red O staining for adipocytes **(b)** at 21 days and 8 days after osteogenic and adipogenic differentiation, respectively. **(c-d)** BMMSCs from KO or WT mice were treated with 200 ng/mL rabbit anti-mouse Lcn2 neutralizing antibody or non-immune rabbit IgG throughout the differentiation process of BMMSCs into osteoblasts or adipocytes. Alizarin Red S staining for osteoblasts **(c)** and Oil Red O staining for adipocytes **(d)** were performed at 21 days or 8 days after differentiation, respectively. **(e-f)** 100 ng/mL of recombinant mouse Ccl2 protein (rmCcl2) or 10 nM CCR2 antagonist INCB3344 or vehicle control was supplemented into the adipogenic and osteogenic induction medium during BMMSC differentiation. Alizarin Red S staining **(e)** and Oil Red O staining **(f)** were performed at 21 days or 8 days after differentiation, respectively. **(a-f)** The bar charts in the right panel represent densitometric measurement of OD405 for differentiated osteoblasts or OD520 absorbance for differentiated adipocytes. *n*=5 for each group. The data are presented as the means ± SEMs, ***p* < 0.01, ****p* < 0.001.


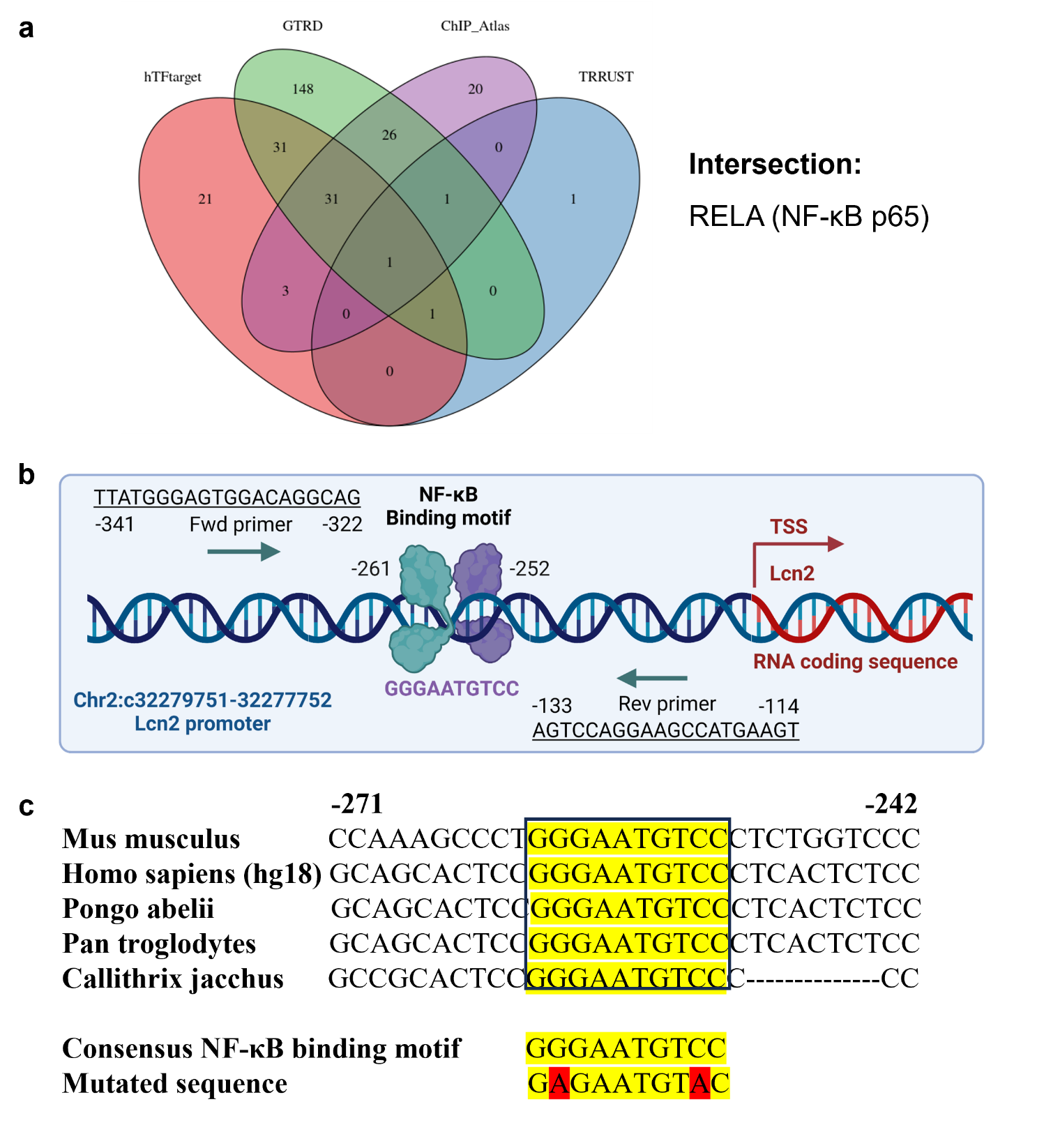


**Figure S15. Related to Figure 7. Identification of NF-κB as a potential transcription factor that controls the Lcn2 gene expression. (a)** Prediction of potential transcription factors regulating the *Lcn2* gene expression with 4 different databases: hTFtarget, GTRD, ChIP_Atlas, and TRRUST. NF-κB was identified as the only transcription factor by all four databases by TF Target Finder (https://jingle.shinyapps.io/TF_Target_Finder/). **(b)** Schematic diagram showing the NF-κB binding motif in the *Lcn2* promoter region, and primers designed for chromatin immunoprecipitation; created with BioRender (https://BioRender.com). **(c)** Sequence alignment showing the conserved NF-κB binding sequences (highlighted in yellow) in the *Lcn2* promoter region among different species. The mutations made in the NF-κB binding sites of the *Lcn2* promoter region for the luciferase reporter assay were highlighted in red.


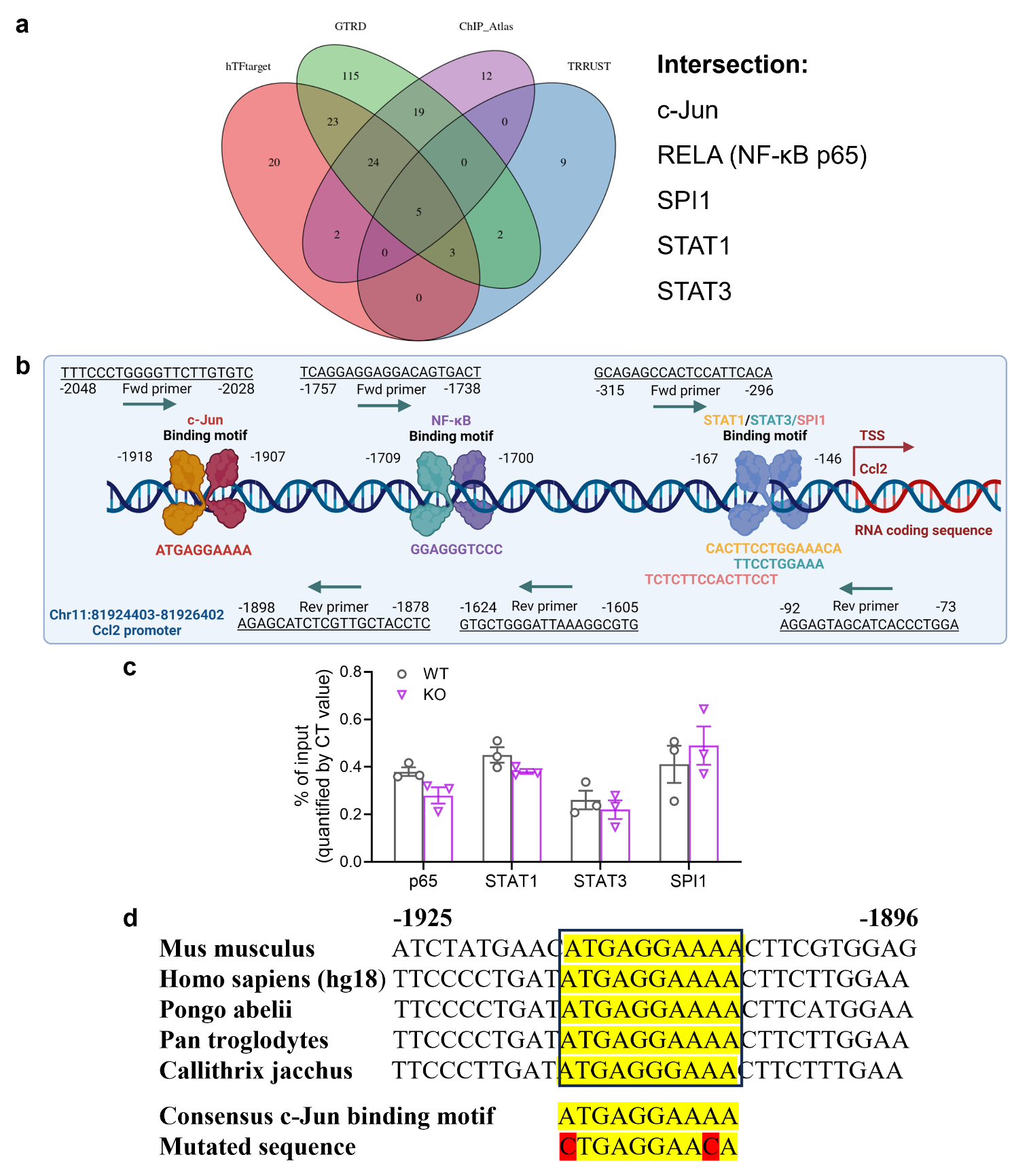


**Figure S16. Related to Figure 7. Transcription factors that potentially regulate Ccl2 expression. (a)** Prediction of potential transcription factors regulating the *Ccl2* gene expression with 4 different databases: hTFtarget, GTRD, ChIP_Atlas, and TRRUST. Five potential transcriptional regulators were identified by TF Target Finder (https://jingle.shinyapps.io/TF_Target_Finder/). **(b)** Schematic diagram showing the c-Jun, NF-κB, STAT1, STAT3, and SPI1 binding motifs in the *Ccl2* promoter region, and primers designed for chromatin immunoprecipitation; created with BioRender (https://BioRender.com). **(c)** Sonicated BMMSCs were subjected to chromatin immunoprecipitation using anti-p65, anti-STAT1, anti-STAT3, and anti-SPI1 antibodies, respectively. The ChIP DNA samples were subjected to qPCR amplification using specific primers against the promoter region of *Ccl2* as shown in **(b)**. Quantitative results are shown as the percentage of input DNA: % of Input=2^(CT^Input^−CT^IP^)/dilution factor×100%. **(d)** Sequence alignment showing the conserved c-Jun binding sequences (highlighted in yellow) in the *Ccl2* promoter region among different species. The mutations made in the c-Jun binding sites of the *Ccl2* promoter region for the luciferase reporter assay were highlighted in red.


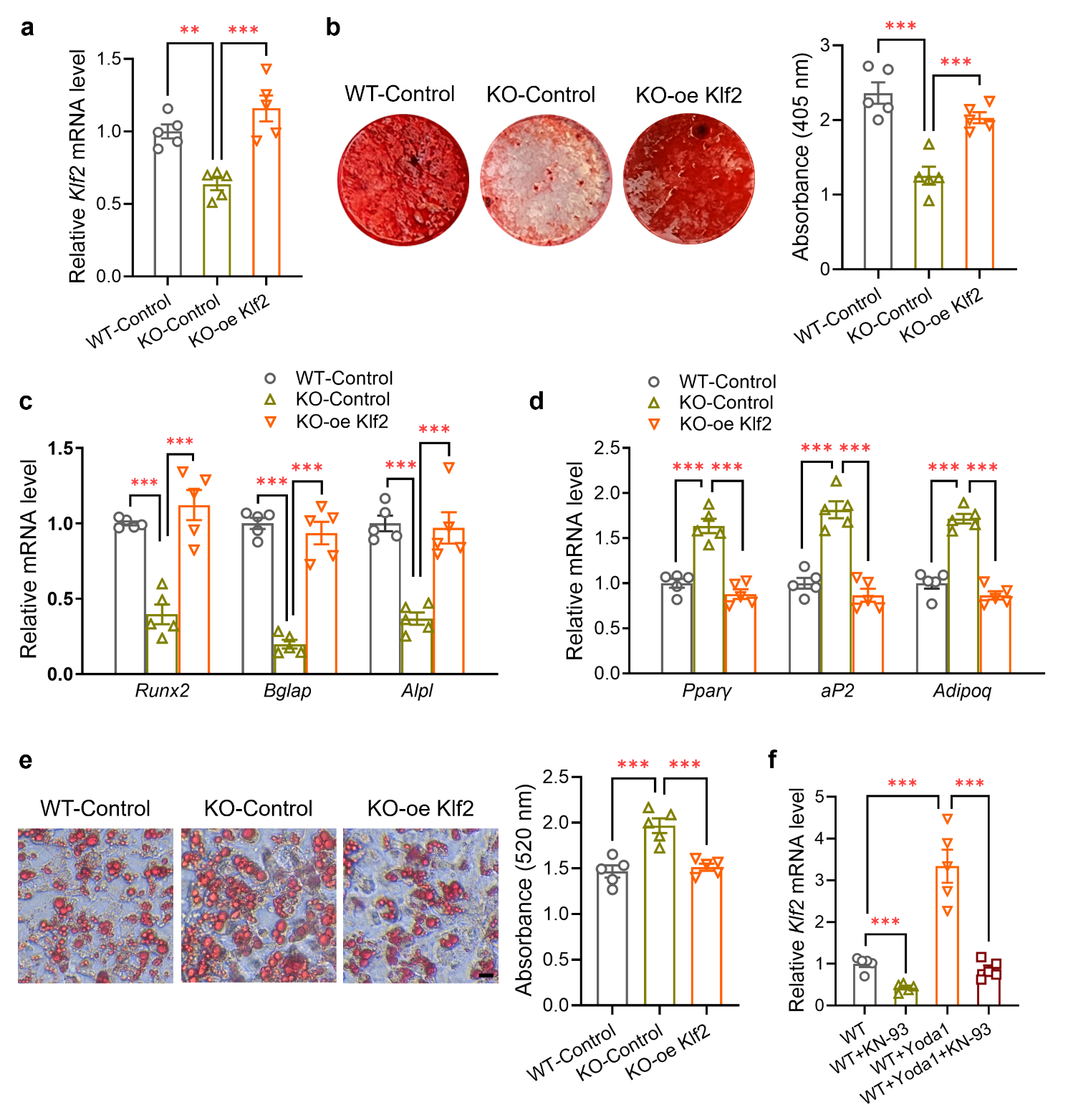


**Figure S17. Related to Figure 7. Overexpression of Klf2 reverses the decreased osteogenesis and enhanced adipogenesis of KO BMMSCs. (a-e)** BMMSCs isolated from femurs and tibias of 10-week-old male PDGFRα-Piezo1 KO mice and their WT littermates were infected with lentivirus (MOI=40) encoding *eGFP* together with mouse *Klf2* gene (oe Klf2) or *eGFP* only (Control) for 72 hours with the presence of 5 μg/mL polybrene before differentiation. **(a)** Gene expression of *Klf2* in BMMSCs determined by real-time PCR. **(b)** Alizarin Red S staining of osteoblasts at Day 21 after differentiation. The right panel is the quantitative analysis of Alizarin Red S staining by determining OD405 absorbance values. **(c-d)** BMMSCs with lentivirus infections were then differentiated into osteoblasts or adipocytes, respectively. The expression levels of osteogenic genes **(c)** and adipogenic genes **(d)** were determined by real-time qPCR. **(e)** Oil Red O staining of BMMSCs-derived BMAs at day 8 after differentiation. Scale bar, 50 μm. The right panel is the quantitative analysis of Oil Red O staining by determining OD520 absorbance values. **(f)** BMMSCs isolated from femurs and tibias of 10-week-old male C57BL/6J mice were treated with CaMKII inhibitor KN-93 (10 μM) and/or Piezo1 activator Yoda1 (5 μM) for 2 hours. The mRNA level of *Klf2* was determined by real-time PCR. *n*=5 for each group. The data are presented as the means ± SEMs, ***p* < 0.01, ****p* < 0.001.


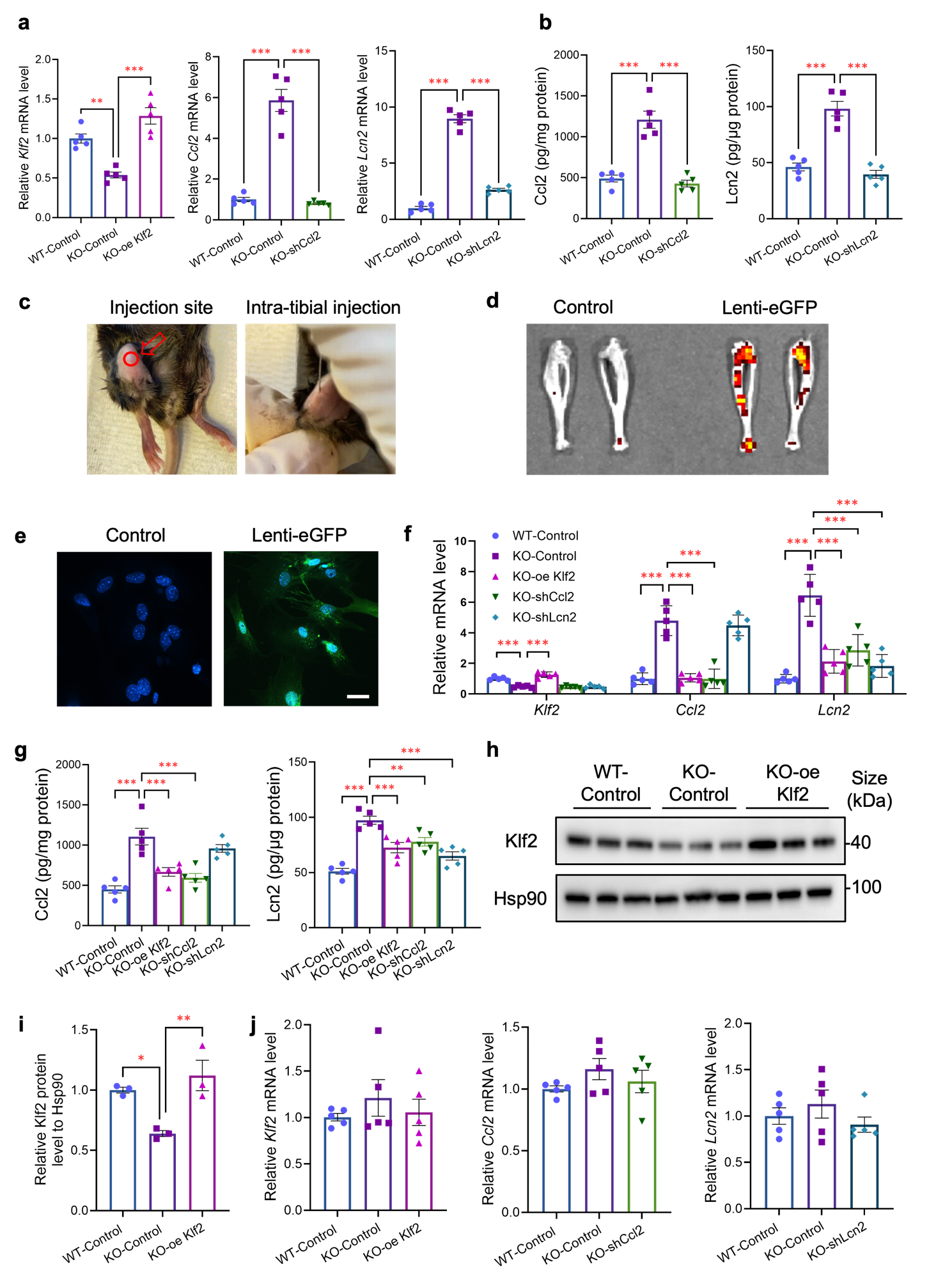


**Figure S18. Related to Figure 8. *In vitro* and *in vivo* validation of gene manipulations in BMMSCs mediated by lentivirus. (a-b)** BMMSCs were isolated from the bone marrow of femurs and tibias of PDGFRα-Piezo1 KO mice or their WT littermates were infected with lentivirus (MOI=40) encoding *eGFP* together with scrambled shRNA (WT-Control or KO-Control), shRNA against *Ccl2* (KO-shCcl2) or *Lcn2* (KO-shLcn2), or overexpression of mouse *Klf2* gene (KO-oe Klf2) driven by PDGFRα promoter for 72 hours with the presence of 5 μg/mL polybrene. **(a)** qPCR analysis of *Ccl2*, *Lcn2*, and *Klf2* expression in BMMSCs. **(b)** ELISA analysis of Ccl2 and Lcn2 in the conditioned medium of BMMSCs. **(c-j)** Eight-week-old male PDGFRα-Piezo1 KO mice and their WT littermates were subjected to bilateral tibial injection of lentivirus encoding *eGFP* together with scrambled shRNA (Control), shRNA against *Ccl2* (shCcl2) or *Lcn2* (shLcn2), or overexpression of mouse *Klf2* gene (oe Klf2) driven by PDGFRα promoter at a dose of 1.25 × 10^6^ TU per side for 7 weeks. **(c)** Images showing the site of tibial injection. **(d)** Representative IVIS image showing GFP fluorescence in isolated tibias. **(e)** Fluorescence microscopy showing GFP signal (green) in isolated BMMSCs. Nuclei were stained with DAPI (blue). Scale bar, 25 μm. **(f)** qPCR analysis of *Ccl2*, *Lcn2*, and *Klf2* expression in BMMSCs isolated from the tibias of lentivirus-injected mice. **(g)** ELISA analysis of Ccl2 and Lcn2 levels in the culture medium of BMMSCs isolated from the tibias of lentivirus-injected mice. **(h)** Western blot analysis of Klf2 in BMMSCs isolated from the tibias of lentivirus-injected mice. **(i)** Densitometric analysis for the relative abundance of Klf2 normalized with HSP90. **(j)** qPCR analysis of *Lcn2*, *Ccl2*, and *Klf2* mRNA levels in SVFs isolated from scWAT of mice receiving tibial injection of lentivirus. *n*=3-5 for each group. The data are presented as the means ± SEMs, **p* < 0.05, ***p* < 0.01, ****p* < 0.001.

**Table S1. Combined expression panel**

| Method | Target name | | WT | | | | | KO | | | | | | Target name | Mean Fold Change | P value | SE of difference |
| --- | --- | --- | --- | --- | --- | --- | --- | --- | --- | --- | --- | --- | --- | --- | --- | --- | --- |
| ELISA | Lcn2 | | 0.89602 | 1.13938 | 0.6761 | 1.14221 | 1.1463 | 4.71317 | 3.81967 | | 3.36602 | 2.42522 | 4.25456 | Lcn2 | 3.715727126 | 0.00015 | 0.4038 |
| Olink | Ccl2 | | 1.09003 | 0.99038 | 0.90559 | 0.71161 | 1.30238 | 2.9592 | 1.82545 | | 3.10899 | 5.54073 | 1.7063 | Ccl2 | 3.028134123 | 0.01957 | 0.6968 |
| Olink | S100a4 | | 1.05329 | 0.75573 | 0.37937 | 0.58126 | 2.23036 | 4.43457 | 0.61462 | | 1.34299 | 6.98526 | 1.7584 | S100a4 | 3.027167784 | 0.13677 | 1.226 |
| Olink | Prdx5 | | 0.73855 | 1.39738 | 0.73877 | 0.69795 | 1.42736 | 3.35401 | 1.18743 | | 2.78224 | 5.23725 | 2.29622 | Prdx5 | 2.97142877 | 0.02125 | 0.69 |
| Olink | Fst | | 1.03052 | 0.95056 | 1.08105 | 0.94781 | 0.99007 | 2.93239 | 1.50553 | | 1.59805 | 3.53088 | 1.54787 | Fst | 2.222943818 | 0.02028 | 0.4235 |
| Olink | Matn2 | | 1.28818 | 1.25789 | 0.17092 | 1.00803 | 1.275 | 1.48159 | 1.74432 | | 1.93267 | 2.92856 | 2.10667 | Matn2 | 2.038758848 | 0.01276 | 0.3254 |
| Olink | Itgb1bp2 | | 1.09076 | 1.37445 | 0.59287 | 1.07537 | 0.86655 | 2.16799 | 1.48651 | | 1.53271 | 1.69695 | 2.48797 | Itgb1bp2 | 1.874425875 | 0.00579 | 0.2344 |
| Olink | Cxcl1 | | 0.55236 | 1.62008 | 0.93994 | 0.96294 | 0.92468 | 1.7529 | 1.04975 | | 1.55853 | 2.21518 | 1.11349 | Cxcl1 | 1.537970742 | 0.08674 | 0.2756 |
| Olink | Lpl | | 1.00428 | 1.09443 | 0.94801 | 1.24795 | 0.70533 | 1.59066 | 1.29093 | | 1.5746 | 1.57361 | 1.53141 | Lpl | 1.512242426 | 0.00127 | 0.1056 |
| Olink | Riox2 | | 1.371 | 1.20017 | 0.79399 | 1.15446 | 0.48038 | 0.63154 | 1.6046 | | 1.36278 | 1.35363 | 2.53028 | Riox2 | 1.496567861 | 0.18804 | 0.345 |
| Olink | Hgf | | 0.98276 | 0.84381 | 1.11008 | 1.01887 | 1.04448 | 1.62901 | 1.26517 | | 1.40062 | 1.95885 | 1.13447 | Hgf | 1.477624086 | 0.01379 | 0.1521 |
| Olink | Tgfb1 | | 0.95749 | 1.08189 | 1.15658 | 0.93668 | 0.86736 | 1.63362 | 1.10613 | | 1.34434 | 1.42553 | 1.70326 | Tgfb1 | 1.442575893 | 0.00582 | 0.1188 |
| Olink | Ccl5 | | 1.20235 | 0.907 | 1.09666 | 0.59943 | 1.19456 | 1.26406 | 1.31966 | | 1.29471 | 1.07184 | 2.03645 | Ccl5 | 1.397342257 | 0.08314 | 0.2007 |
| Olink | Itgb6 | | 0.92446 | 1.77622 | 0.46903 | 1.09644 | 0.73386 | 1.34133 | 1.40838 | | 0.80479 | 1.56509 | 1.73591 | Itgb6 | 1.371100157 | 0.20741 | 0.2706 |
| Olink | Cxcl9 | | 1.04257 | 1.23429 | 1.11588 | 0.79965 | 0.80761 | 1.7064 | 1.14702 | | 1.80801 | 0.33528 | 1.74042 | Cxcl9 | 1.347424344 | 0.2685 | 0.2922 |
| Olink | Nadk | | 1.05545 | 1.05479 | 0.85463 | 0.98153 | 1.05359 | 1.60393 | 1.25644 | | 1.04555 | 1.74734 | 1.04519 | Nadk | 1.339688159 | 0.05263 | 0.1494 |
| Olink | Vegfd | | 0.87483 | 1.24123 | 0.86791 | 0.97902 | 1.037 | 1.29306 | 1.34065 | | 1.13313 | 1.33822 | 1.26293 | Vegfd | 1.273597557 | 0.00805 | 0.07811 |
| Olink | Tnfsf12 | | 0.68668 | 1.55875 | 1.04912 | 0.90961 | 0.79584 | 1.88293 | 1.01586 | | 0.98263 | 0.83815 | 1.63463 | Tnfsf12 | 1.27083981 | 0.31996 | 0.2554 |
| Olink | Gcg | | 1.11532 | 0.89488 | 0.93224 | 1.19044 | 0.86713 | 1.3362 | 1.26062 | | 1.18362 | 1.12977 | 1.41856 | Gcg | 1.265753546 | 0.01228 | 0.08259 |
| Olink | Il17a | | 0.839 | 1.19796 | 1.05946 | 1.05651 | 0.84707 | 1.20011 | 0.96903 | | 1.1278 | 1.19101 | 1.81133 | Il17a | 1.259855314 | 0.14222 | 0.1596 |
| Olink | Casp3 | | 0.75009 | 1.08381 | 0.90977 | 1.13858 | 1.11774 | 2.32903 | 1.02828 | | 0.85487 | 0.96292 | 1.04544 | Casp3 | 1.244107372 | 0.41384 | 0.2832 |
| Olink | Adam23 | | 1.06078 | 1.14281 | 1.00158 | 0.94694 | 0.84788 | 1.24041 | 0.86831 | | 1.34199 | 0.96164 | 1.79009 | Adam23 | 1.240487508 | 0.19513 | 0.1701 |
| Olink | Dctn2 | | 0.97046 | 1.02922 | 0.85934 | 1.06027 | 1.08071 | 1.29282 | 0.89241 | | 1.26318 | 1.49154 | 1.20101 | Dctn2 | 1.228192329 | 0.06122 | 0.1049 |
| Olink | Tnr | | 0.80723 | 1.34022 | 0.83891 | 1.14112 | 0.87252 | 1.30121 | 1.09688 | | 1.21678 | 1.23264 | 1.2443 | Tnr | 1.21836309 | 0.08005 | 0.109 |
| Olink | Tnni3 | | 0.91259 | 1.40649 | 1.07772 | 0.61512 | 0.98808 | 1.59952 | 0.85583 | | 0.84647 | 1.30474 | 1.47444 | Tnni3 | 1.216198041 | 0.31547 | 0.2019 |
| Olink | Axin1 | | 0.82323 | 0.97183 | 0.98958 | 0.96326 | 1.2521 | 1.12347 | 0.84239 | | 1.25723 | 1.3231 | 1.52957 | Axin1 | 1.215153304 | 0.14583 | 0.1335 |
| Olink | Dll1 | | 0.87936 | 1.18211 | 0.68244 | 1.0128 | 1.24328 | 1.17201 | 0.90907 | | 1.35197 | 0.96297 | 1.66542 | Dll1 | 1.212288146 | 0.25096 | 0.1715 |
| Olink | Il23r | | 1.1092 | 0.98413 | 0.7719 | 1.11506 | 1.01971 | 1.34284 | 0.9889 | | 0.97321 | 1.29897 | 1.33098 | Il23r | 1.186979956 | 0.1127 | 0.105 |
| Olink | Cpe | | 1.01271 | 1.12654 | 0.88298 | 1.10924 | 0.86853 | 1.2869 | 1.0661 | | 1.16023 | 1.2356 | 1.14673 | Cpe | 1.179111905 | 0.02711 | 0.06636 |
| Olink | Ghrl | | 1.25568 | 1.02368 | 1.02069 | 0.83779 | 0.86215 | 1.17845 | 0.89672 | | 1.05216 | 1.49745 | 1.26918 | Ghrl | 1.178791443 | 0.19333 | 0.1259 |
| Olink | Map2k6 | | 0.88866 | 1.06957 | 1.01819 | 0.96587 | 1.0577 | 1.35454 | 1.08395 | | 1.3055 | 1.10362 | 1.02862 | Map2k6 | 1.175245992 | 0.0428 | 0.07285 |
| Olink | Fas | | 1.09408 | 1.09192 | 0.93081 | 0.98568 | 0.8975 | 1.16532 | 0.91751 | | 1.19104 | 1.55771 | 1.04385 | Fas | 1.175086108 | 0.16541 | 0.1147 |
| Olink | Pdgfb | | 0.79073 | 0.94877 | 1.09529 | 1.10428 | 1.06093 | 1.48943 | 1.19763 | | 1.06412 | 1.11094 | 0.95484 | Pdgfb | 1.163393704 | 0.16906 | 0.1081 |
| Olink | Acvrl1 | | 0.94974 | 1.09603 | 0.9474 | 1.08206 | 0.92477 | 1.1681 | 1.28231 | | 0.99051 | 1.09407 | 1.27329 | Acvrl1 | 1.161655172 | 0.0406 | 0.06627 |
| Olink | Tnfrsf12a | | 0.88598 | 1.11145 | 0.9712 | 0.96737 | 1.06399 | 1.09627 | 1.23533 | | 0.97307 | 1.11056 | 1.32237 | Tnfrsf12a | 1.147519922 | 0.07514 | 0.07215 |
| Olink | Cntn4 | | 0.85834 | 0.94963 | 1.12042 | 1.00939 | 1.06222 | 0.79254 | 1.00701 | | 1.30776 | 1.30147 | 1.32867 | Cntn4 | 1.147491232 | 0.23919 | 0.116 |
| Olink | Snap29 | | 0.81383 | 1.25921 | 1.06998 | 0.89737 | 0.95961 | 1.18683 | 0.95204 | | 1.05264 | 1.30455 | 1.2399 | Snap29 | 1.147192615 | 0.18008 | 0.1002 |
| Olink | Epo | | 0.97614 | 1.20242 | 1.14847 | 0.87119 | 0.80177 | 1.01184 | 0.95977 | | 1.09831 | 1.41101 | 1.25143 | Epo | 1.146471062 | 0.23137 | 0.1131 |
| Olink | Tpp1 | | 0.93448 | 1.17142 | 0.91022 | 0.86057 | 1.12332 | 1.50177 | 0.90608 | | 1.0708 | 1.1176 | 1.13141 | Tpp1 | 1.145533767 | 0.24348 | 0.1156 |
| Olink | Il6 | | 0.91224 | 1.22936 | 0.9284 | 0.96369 | 0.96631 | 1.23064 | 0.98935 | | 1.07703 | 1.39017 | 1.03135 | Il6 | 1.14370715 | 0.16523 | 0.0941 |
| Olink | Pak4 | | 0.882 | 1.79761 | 0.83977 | 0.86196 | 0.61867 | 1.18234 | 1.41334 | | 1.00511 | 0.84246 | 1.26579 | Pak4 | 1.141808006 | 0.5512 | 0.228 |
| Olink | Tnf | | 1.10186 | 1.34763 | 0.76197 | 0.8048 | 0.98374 | 1.60298 | 1.00092 | | 1.03968 | 1.1164 | 0.8588 | Tnf | 1.123758084 | 0.47619 | 0.1656 |
| Olink | Clmp | | 0.90395 | 1.12731 | 0.96055 | 1.16857 | 0.83963 | 1.31141 | 1.06793 | | 1.12173 | 1.04019 | 1.06799 | Clmp | 1.121847171 | 0.16848 | 0.08048 |
| Olink | Parp1 | | 1.23777 | 1.02139 | 0.8605 | 0.83152 | 1.04882 | 1.34241 | 0.98472 | | 0.896 | 1.26308 | 1.10742 | Parp1 | 1.118727956 | 0.31542 | 0.1109 |
| Olink | Eda2r | | 0.90212 | 1.34428 | 0.96885 | 0.86687 | 0.91787 | 1.10153 | 1.22665 | | 1.05845 | 0.99712 | 1.20988 | Eda2r | 1.118725423 | 0.26044 | 0.09804 |
| Olink | Ccl3 | | 1.03264 | 1.04101 | 0.9906 | 0.92745 | 1.0083 | 1.18869 | 1.14957 | | 1.07255 | 1.09969 | 1.04319 | Ccl3 | 1.11073671 | 0.01013 | 0.03309 |
| Olink | Mia | | 0.99407 | 1.13001 | 1.01983 | 0.8681 | 0.98799 | 1.24421 | 1.01158 | | 1.05787 | 1.07851 | 1.1359 | Mia | 1.105612022 | 0.10517 | 0.05782 |
| Olink | Fstl3 | | 0.99398 | 1.12121 | 0.86758 | 0.81241 | 1.20481 | 1.41456 | 0.7469 | | 0.96806 | 1.23632 | 1.12262 | Fstl3 | 1.097691574 | 0.49277 | 0.1359 |
| Olink | Yes1 | | 1.18626 | 1.11704 | 1.03668 | 0.91923 | 0.7408 | 1.18507 | 1.12737 | | 0.98098 | 1.02431 | 1.16529 | Yes1 | 1.096603748 | 0.30519 | 0.08818 |
| Olink | Cdh6 | | 0.85617 | 1.34716 | 0.93862 | 0.88571 | 0.97234 | 1.01037 | 0.90041 | | 1.09306 | 1.24126 | 1.23659 | Cdh6 | 1.096339179 | 0.40966 | 0.1107 |
| Olink | Cant1 | | 0.72489 | 1.03185 | 0.94174 | 1.20066 | 1.10085 | 1.04646 | 0.90662 | | 0.78292 | 1.27185 | 1.46484 | Cant1 | 1.094539612 | 0.53889 | 0.1473 |
| Olink | Crim1 | | 1.02117 | 1.05684 | 0.61944 | 1.22466 | 1.0779 | 0.77913 | 0.87341 | | 1.0581 | 0.60671 | 2.14782 | Crim1 | 1.093032944 | 0.75797 | 0.2917 |
| Olink | Igsf3 | | 1.0581 | 1.1425 | 0.80343 | 1.00744 | 0.98853 | 1.19268 | 0.8113 | | 1.16034 | 1.28929 | 0.9831 | Igsf3 | 1.087340965 | 0.41535 | 0.1017 |
| Olink | Tnfrsf11b | | 1.02433 | 1.13891 | 0.95187 | 1.02301 | 0.86188 | 1.3202 | 0.79724 | | 0.96828 | 1.2561 | 1.07018 | Tnfrsf11b | 1.082399517 | 0.45767 | 0.1056 |
| Olink | Foxo1 | | 0.95056 | 1.03871 | 0.95062 | 1.12828 | 0.93183 | 1.23587 | 0.97562 | | 0.88769 | 1.20875 | 1.09959 | Foxo1 | 1.08150246 | 0.31695 | 0.07636 |
| Olink | Lgmn | | 1.02529 | 0.95543 | 0.98611 | 0.87116 | 1.16201 | 1.26334 | 1.18367 | | 1.00492 | 0.98049 | 0.96614 | Lgmn | 1.079711464 | 0.33074 | 0.07698 |
| Olink | Gdnf | | 1.02816 | 1.17581 | 0.68195 | 1.22804 | 0.88604 | 1.01041 | 0.81508 | | 1.01928 | 1.2664 | 1.27211 | Gdnf | 1.076656128 | 0.57724 | 0.1319 |
| Olink | Wfikkn2 | | 0.9572 | 1.16754 | 0.9451 | 0.98284 | 0.94732 | 1.14216 | 0.92435 | | 1.04595 | 1.08886 | 1.17109 | Wfikkn2 | 1.074481354 | 0.25401 | 0.06061 |
| Olink | Ntf3 | | 0.98998 | 1.30484 | 0.94976 | 0.90015 | 0.85527 | 1.36 | 0.90651 | | 1.07013 | 0.90355 | 1.10835 | Ntf3 | 1.069709773 | 0.56263 | 0.1154 |
| Olink | Kitlg | | 0.60489 | 1.27841 | 0.84122 | 1.31525 | 0.96023 | 1.15453 | 0.82283 | | 0.99786 | 1.33678 | 1.03253 | Kitlg | 1.068904316 | 0.67626 | 0.159 |
| Olink | Gfra1 | | 1.00798 | 1.23537 | 0.81469 | 0.94433 | 0.99762 | 0.91684 | 1.29575 | | 0.84866 | 1.07009 | 1.19659 | Gfra1 | 1.065586641 | 0.55965 | 0.1078 |
| Olink | Apbb1ip | | 0.99067 | 1.09898 | 1.05512 | 0.92091 | 0.93432 | 1.10678 | 0.91584 | | 1.0999 | 1.0383 | 1.15488 | Apbb1ip | 1.063142143 | 0.27272 | 0.05361 |
| Olink | Ppp1r2 | | 0.99284 | 0.70974 | 0.9112 | 1.41441 | 0.97181 | 1.34068 | 0.74668 | | 0.70411 | 1.2339 | 1.27843 | Ppp1r2 | 1.060757783 | 0.74407 | 0.1798 |
| Olink | Pla2g4a | | 0.94967 | 1.20324 | 0.89263 | 1.09527 | 0.85918 | 1.04138 | 0.98305 | | 1.00305 | 1.18196 | 1.04396 | Pla2g4a | 1.050682068 | 0.51081 | 0.07365 |
| Olink | Ddah1 | | 0.8626 | 0.87572 | 1.25647 | 0.90555 | 1.09966 | 1.26331 | 0.73961 | | 0.99519 | 1.21693 | 1.02243 | Ddah1 | 1.047493337 | 0.70473 | 0.1209 |
| Olink | Tgfbr3 | | 0.89284 | 1.00829 | 1.23205 | 0.92332 | 0.9435 | 1.46841 | 0.76388 | | 1.00146 | 1.04323 | 0.95713 | Tgfbr3 | 1.046820262 | 0.72972 | 0.1308 |
| Olink | Flrt2 | | 1.06695 | 1.08351 | 0.92478 | 0.9925 | 0.93226 | 1.14843 | 0.92486 | | 0.9018 | 1.16622 | 1.08366 | Flrt2 | 1.044996786 | 0.50612 | 0.06465 |
| Olink | Ahr | | 0.86865 | 1.24742 | 0.96274 | 1.01559 | 0.9056 | 0.98609 | 1.02166 | | 1.11758 | 0.93211 | 1.14679 | Ahr | 1.040845977 | 0.61418 | 0.07788 |
| Olink | Plxna4 | | 0.8051 | 1.43434 | 1.02495 | 0.90035 | 0.83525 | 1.36145 | 0.85293 | | 0.9987 | 0.87018 | 1.1208 | Plxna4 | 1.040812692 | 0.7901 | 0.1483 |
| Olink | Fli1 | | 0.95471 | 0.90647 | 1.03811 | 1.17549 | 0.92521 | 0.89949 | 0.78298 | | 1.39214 | 1.04894 | 1.07595 | Fli1 | 1.039900018 | 0.73533 | 0.114 |
| Olink | Il1a | | 0.81194 | 1.76088 | 1.03967 | 0.34151 | 1.046 | 0.56543 | 0.71506 | | 0.44891 | 1.22245 | 2.22083 | Il1a | 1.034538324 | 0.93289 | 0.3974 |
| Olink | Il17f | | 0.81965 | 1.26442 | 1.04477 | 0.94943 | 0.92173 | 1.08853 | 0.79068 | | 1.15831 | 1.01192 | 1.09104 | Il17f | 1.028095237 | 0.78287 | 0.09857 |
| Olink | Eno2 | | 0.88959 | 1.23953 | 0.95759 | 1.08041 | 0.83287 | 1.06398 | 0.82151 | | 0.96096 | 1.09938 | 1.17566 | Eno2 | 1.024297103 | 0.80486 | 0.09514 |
| Olink | Clstn2 | | 0.99967 | 1.15335 | 0.70359 | 0.80332 | 1.34006 | 1.22717 | 0.90547 | | 0.72639 | 0.84531 | 1.39426 | Clstn2 | 1.019719951 | 0.91055 | 0.1701 |
| Multiplex assay | Ccl7 | | 0.93987 | 1.07891 | 1.54486 | 0.46295 | 0.97341 | 1.0131 | 1.44909 | | 0.5726 | 1.50682 | 0.52832 | Ccl7 | 1.013984763 | 0.95997 | 0.2701 |
| Olink | Tgfa | | 0.92471 | 1.08254 | 1.11861 | 0.95414 | 0.91999 | 1.07431 | 0.91416 | | 1.05152 | 0.96456 | 1.06516 | Tgfa | 1.013941957 | 0.7973 | 0.0525 |
| Olink | Il5 | | 0.95372 | 0.89644 | 1.14855 | 1.28609 | 0.71521 | 0.92789 | 0.60359 | | 0.88502 | 1.01649 | 1.55785 | Il5 | 0.998168331 | 0.99235 | 0.1851 |
| Olink | Il1b | | 0.86268 | 1.25864 | 1.03436 | 0.88212 | 0.9622 | 0.992 | 0.92673 | | 0.89815 | 1.1477 | 1.00409 | Il1b | 0.99373397 | 0.94208 | 0.08358 |
| Olink | Sez6l2 | | 0.8275 | 1.15445 | 0.91843 | 0.87486 | 1.22476 | 1.01832 | 0.93282 | | 0.89191 | 1.00843 | 1.10211 | Sez6l2 | 0.990719107 | 0.91812 | 0.08748 |
| Olink | Wisp1 | | 0.8852 | 1.12945 | 1.08393 | 0.78771 | 1.11371 | 1.08102 | 0.88315 | | 0.92983 | 1.05553 | 0.98068 | Wisp1 | 0.986041284 | 0.86293 | 0.07829 |
| Olink | Csf2 | | 0.98187 | 1.07484 | 0.96991 | 1.0712 | 0.90217 | 1.30366 | 0.9082 | | 0.91076 | 1.01499 | 0.77988 | Csf2 | 0.983498039 | 0.86526 | 0.09418 |
| Olink | Cntn1 | | 0.83714 | 1.24568 | 0.93242 | 0.84296 | 1.14181 | 0.90277 | 0.88516 | | 1.05675 | 0.96169 | 1.10184 | Cntn1 | 0.981644465 | 0.84814 | 0.0928 |
| Olink | Qdpr | | 0.91512 | 0.74796 | 0.93088 | 1.58134 | 0.82471 | 1.1826 | 0.58505 | | 0.85542 | 1.30865 | 0.97186 | Qdpr | 0.980719207 | 0.92388 | 0.1956 |
| Olink | Erbb4 | | 0.86624 | 1.28217 | 0.9688 | 1.05348 | 0.82931 | 0.98399 | 0.89732 | | 0.7887 | 1.33427 | 0.89705 | Erbb4 | 0.980266256 | 0.87726 | 0.1238 |
| Olink | Il10 | | 1.21658 | 1.01268 | 0.99202 | 0.84938 | 0.92933 | 0.6978 | 1.01776 | | 1.35267 | 0.84171 | 0.97576 | Il10 | 0.977140748 | 0.85975 | 0.1253 |
| Olink | Epcam | | 0.78484 | 1.72579 | 0.73819 | 0.8447 | 0.90649 | 0.54585 | 1.02374 | | 1.16304 | 0.73699 | 1.34816 | Epcam | 0.963556037 | 0.8799 | 0.2336 |
| ELISA | Fabp4 | | 0.69127 | 0.72573 | 1.23985 | 1.33094 | 1.01221 | 0.96378 | 1.11175 | | 0.86496 | 0.90789 | 0.95024 | Fabp4 | 0.959724844 | 0.77546 | 0.1365 |
| Olink | Cyr61 | | 0.87954 | 1.20267 | 0.85165 | 1.10224 | 0.96391 | 0.97723 | 0.7453 | | 0.96075 | 1.08572 | 0.99395 | Cyr61 | 0.952589806 | 0.60188 | 0.0873 |
| Olink | Ca13 | | 0.59126 | 1.47672 | 0.86684 | 1.06838 | 0.9968 | 1.33667 | 0.71193 | | 0.79382 | 0.91767 | 0.92981 | Ca13 | 0.937980666 | 0.73932 | 0.18 |
| Olink | Vsig2 | | 1.41936 | 0.96134 | 0.67778 | 1.25887 | 0.68264 | 1.29495 | 0.52133 | | 0.63497 | 0.90746 | 1.21846 | Vsig2 | 0.915434865 | 0.70349 | 0.2143 |
| Olink | Dlk1 | | 1.06477 | 1.02255 | 0.91175 | 0.94276 | 1.05818 | 1.00545 | 0.94323 | | 0.88525 | 0.83048 | 0.91122 | Dlk1 | 0.915124592 | 0.08105 | 0.04253 |
| Olink | Rgma | | 1.02564 | 0.98436 | 0.9098 | 0.97487 | 1.10532 | 0.85663 | 0.61097 | | 0.94118 | 0.9185 | 1.17169 | Rgma | 0.899793248 | 0.32425 | 0.0954 |
| Olink | Notch3 | | 0.73531 | 1.46045 | 0.8923 | 0.96184 | 0.9501 | 0.60889 | 0.8676 | | 0.7322 | 1.16643 | 0.99538 | Notch3 | 0.87409938 | 0.44368 | 0.1563 |
| Olink | Ccl20 | | 0.94866 | 1.2662 | 0.81171 | 1.13468 | 0.83875 | 0.69751 | 0.64341 | | 0.92142 | 0.81983 | 1.28819 | Ccl20 | 0.874072716 | 0.40703 | 0.1439 |
| Multiplex assay | Cxcl12 | | 0.8365 | 0.95626 | 0.93832 | 1.23153 | 1.03739 | 0.82221 | 0.79725 | | 0.82484 | 0.85593 | 0.82688 | Cxcl12 | 0.825419777 | 0.03092 | 0.06678 |
| Multiplex assay | Igf1 | | 0.57199 | 0.68284 | 0.68011 | 1.0081 | 2.05696 | 0.67304 | 1.43151 | | 0.83463 | 0.72413 | 0.42453 | Igf1 | 0.817569376 | 0.58575 | 0.3213 |
| Olink | Plin1 | | 0.86015 | 1.10676 | 1.04038 | 1.13629 | 0.85642 | 0.97095 | 0.56283 | | 0.77258 | 0.79779 | 0.6105 | Plin1 | 0.742928387 | 0.02594 | 0.09424 |
| **Ranking by fold change** | | | (Fold change>2 or fold change<0.5, p-value<0.05) | | | | | | | | | | | | | | |
| **Method** | | **Target name** | **WT** | | | | | **KO** | | | | | | **Target name** | **Mean Fold Change** | **P value** | **SE of difference** |
| ELISA | | Lcn2 | 0.89602 | 1.13938 | 0.6761 | 1.14221 | 1.1463 | 4.71317 | | 3.81967 | 3.36602 | 2.42522 | 4.25456 | Lcn2 | 3.715727126 | 0.00015 | 0.4038 |
| Olink | | Ccl2 | 1.09003 | 0.99038 | 0.90559 | 0.71161 | 1.30238 | 2.9592 | | 1.82545 | 3.10899 | 5.54073 | 1.7063 | Ccl2 | 3.028134123 | 0.01957 | 0.6968 |
| Olink | | Prdx5 | 0.73855 | 1.39738 | 0.73877 | 0.69795 | 1.42736 | 3.35401 | | 1.18743 | 2.78224 | 5.23725 | 2.29622 | Prdx5 | 2.97142877 | 0.02125 | 0.69 |
| Olink | | Fst | 1.03052 | 0.95056 | 1.08105 | 0.94781 | 0.99007 | 2.93239 | | 1.50553 | 1.59805 | 3.53088 | 1.54787 | Fst | 2.222943818 | 0.02028 | 0.4235 |
| Olink | | Matn2 | 1.28818 | 1.25789 | 0.17092 | 1.00803 | 1.275 | 1.48159 | | 1.74432 | 1.93267 | 2.92856 | 2.10667 | Matn2 | 2.038758848 | 0.01276 | 0.3254 |

**Table S1. Related to Figure 5. Combined expression panel of soluble factors determined by different methods.** The expression levels of secreted proteins from WT and KO BMMSCs were measured using Olink, ELISA, and multiplex assays. The mean fold change, p values, and standard error (SE) of the differences were calculated. Proteins were filtered based on a fold change > 2 or < 0.5, with a p-value < 0.05. The results were ranked in descending order according to fold change. *n*=5 for each group.

**Table S2. Sequences of shRNA**

| **Name** | **Target sequences (5'-3')** |
| --- | --- |
| *shCcl2* | GCAAGATGATCCCAATGAGTA |
| *shLcn2* | CAGGCAATGCGGTCCAGAAAAA |
| *Scramble* | CCTAAGGTTAAGTCGCCCTCG |

**Table S3. Summary of primer pairs for qPCR**

| **Target** | **Species** | **Primer sequence (5'-3')** |
| --- | --- | --- |
| *β-actin*-F | Mouse | CATTGCTGACAGGATGCAGAAGG |
| *β-actin*-R | Mouse | TGCTGGAAGGTGGACAGTGAGG |
| *Piezo1*-F | Mouse | ACATTGCATCCTCGCTGTCA |
| *Piezo1*-R | Mouse | TCCAGATGTGAATCGCCACC |
| *Piezo2*-F | Mouse | CAGGTTCTGGCTGAATTTCC |
| *Piezo2*-F | Mouse | TGGACCGTGCACTCTACCT |
| *Ccl2*-F | Mouse | CAGGTCCCTGTCATGCTTCT |
| *Ccl2*-R | Mouse | GAGTGGGGCGTTAACTGCAT |
| *Prdx5* F | Mouse | AGTCCCTGGGGCATTTACAC |
| *Prdx5* R | Mouse | TAAGTCTGTCGCCTTCCCA |
| *Fst*-F | Mouse | GCCAGTGACAATGCCACATACG |
| *Fst*-R | Mouse | CTTCCTCCGTTTCTTCCGAGATG |
| *Matn2*-F | Mouse | TGCCGTTGGGGTAGGAAAAG |
| *Matn2*-R | Mouse | TTATGGTGACTGGCTCAGGTTC |
| *Lcn2*-F | Mouse | CCAGTTCGCCATGGTATTTT |
| *Lcn2*-R | Mouse | CACACTCACCACCCATTCAG |
| *Runx2*-F | Mouse | CCTGAACTCTGCACCAAGTCCT |
| *Runx2*-R | Mouse | TCATCTGGCTCAGATAGGAGGG |
| *Alpl*-F | Mouse | CCAGAAAGACACCTTGACTGTGG |
| *Alpl*-R | Mouse | TCTTGTCCGTGTCGCTCACCAT |
| *Bglap*-F | Mouse | GAACAGACAAGTCCCACACAGC |
| *Bglap*-R | Mouse | TCAGCAGAGTGAGCAGAAAGAT |
| *Bmp2*-F | Mouse | GCCAAACACAAACAGCGGAA |
| *Bmp2*-R | Mouse | CCAGTCATTCCACCCCACAT |
| *Adipoq*-F | Mouse | ATCTGGAGGTGGGAGACCAA |
| *Adipoq*-R | Mouse | GGGCTATGGGTAGTTGCAGT |
| *C/ebpα*-F | Mouse | TGGAGACGCAACAGAAGGTG |
| *C/ebpα*-R | Mouse | CAGCCTAGAGATCCAGCGAC |
| *aP2*-F | Mouse | GTGTGATGCCTTTGTGGGAAC |
| *aP2*-R | Mouse | CCCCGCCATCTAGGGTTATGA |
| *Pparγ*-F | Mouse | GTACTGTCGGTTTCAGAAGTGCC |
| *Pparγ*-R | Mouse | ATCTCCGCCAACAGCTTCTCCT |
| *GAPDH*-F | Human | AATGGGCAGCCGTTAGGAAA |
| *GAPDH*-R | Human | GCCCAATACGACCAAATCAGAG |
| *ADIPOQ*-F | Human | AACATGCCCATTCGCTTTACC |
| *ADIPOQ*-R | Human | TAGGCAAAGTAGTACAGCCCA |
| *PPARγ*-F | Human | TTCCATTCACAAGAACAGATCC |
| *PPARγ*-R | Human | CTTTGATTGCACTTTGGTACTC |
| *C/EBPα*-F | Human | ACTGGGACCCTCAGCCTTG |
| *C/EBPα*-R | Human | TGGACTGATCGTGCTTCGTG |
| *aP2*-F | Human | TGTGCAGAAATGGGATGGAAA |
| *aP2*-R | Human | CAACGTCCCTTGGCTTATGCT |
| *RUNX2*-F | Human | CCACCACTCACTACCACACC |
| *RUNX2*-R | Human | TCAGCGTCAACACCATCATT |
| *BMP2*-F | Human | TGTATCGCAGGCACTCAGGTCA |
| *BMP2*-R | Human | CCACTCGTTTCTGGTAGTTCTTC |
| *BGLAP*-F | Human | CTCACACTCCTCGCCCTATT |
| *BGLAP*-R | Human | TCAGCCAACTCGTCACAGTC |
| *ALPL*-F | Human | CACCCACGTCGATTGCATCT |
| *ALPL*-R | Human | TAGCCACGTTGGTGTTGAGC |
| *Lcn2-p65*-F | Mouse | TTATGGGAGTGGACAGGCAG |
| *Lcn2-p65*-R | Mouse | AGTCCAGGAAGCCATGAAGT |
| *Ccl2-p65*-F | Mouse | TCAGGAGGAGGACAGTGACT |
| *Ccl2-p65*-R | Mouse | GTGCTGGGATTAAAGGCGTG |
| *Ccl2-Jun*-F | Mouse | TTTCCCTGGGGTTCTTGTGTC |
| *Ccl2-Jun*-R | Mouse | AGAGCATCTCGTTGCTACCTC |
| *Ccl2-Stat1/Stat3/Spi1*-F | Mouse | GCAGAGCCACTCCATTCACA |
| *Ccl2-Stat1/Stat3/Spi1*-R | Mouse | AGGAGTAGCATCACCCTGGA |
| *Myf5-*F | Mouse | GGTGGAGAACTATTACAGCCTGC |
| *Myf5-*R | Mouse | ACAGTAGATGCTGTCAAAGCTGC |
| *Myod-*F | Mouse | GCACTACAGTGGCGACTCAGAT |
| *Myod-*R | Mouse | TAGTAGGCGGTGTCGTAGCCAT |
| *Mstn-*F | Mouse | AGTGGATCTAAATGAGGGCAGT |
| *Mstn-*R | Mouse | GTTTCCAGGCGCAGCTTAC |
| *Myog-*F | Mouse | GAGACATCCCCCTATTTCTACCA |
| *Myog-*R | Mouse | GCTCAGTCCGCTCATAGCC |
| *Pgc -1α-*F | Mouse | AGCCGTGACCACTGACAACGAG |
| *Pgc-1α-*R | Mouse | GCTGCATGGTTCTGAGTGCTAAG |
| *p16^Ink4a^*-F | Mouse | TGTTGAGGCTAGAGAGGATCTTG |
| *p16^Ink4a^* -R | Mouse | CGAATCTGCACCGTAGTTGAGC |
| *p21^Cip1^*-F | Mouse | AAGTGTGCCGTTGTCTCTTC |
| *p21^Cip1^*-R | Mouse | AGTCAAAGTTCCACCGTTCTC |
| *Ki67*-F | Mouse | GAGGAGAAACGCCAACCAAGAG |
| *Ki67*-R | Mouse | TTTGTCCTCGGTGGCGTTATCC |
| *Sox2*-F | Mouse | AACGGCAGCTACAGCATGATGC |
| *Sox2*-R | Mouse | CGAGCTGGTCATGGAGTTGTAC |
| *Oct4*-F | Mouse | CAGCAGATCACTCACATCGCCA |
| *Oct4*-R | Mouse | GCCTCATACTCTTCTCGTTGGG |
| *Nanog*-F | Mouse | GAACGCCTCATCAATGCCTGCA |
| *Nanog*-R | Mouse | GAATCAGGGCTGCCTTGAAGAG |
