# Supplementary material for: Piezo1 activation suppresses bone marrow adipogenesis to prevent osteoporosis by inhibiting a mechanoinflammatory autocrine loop
Source: Signal Transduct Target Ther. 2025 Oct 28;10:357. doi: 10.1038/s41392-025-02455-w (PMC12559722; doi:10.1038/s41392-025-02455-w)
Supplement: Supplementary file 2 — Uncropped blot and gel [file 41392_2025_2455_MOESM2_ESM.docx]

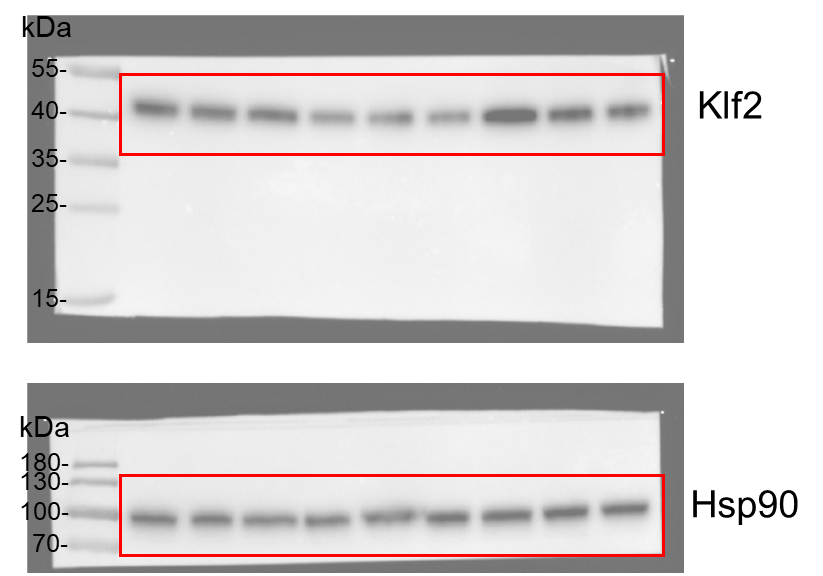


Original and uncropped films of Western blots

Fig. S18h

**
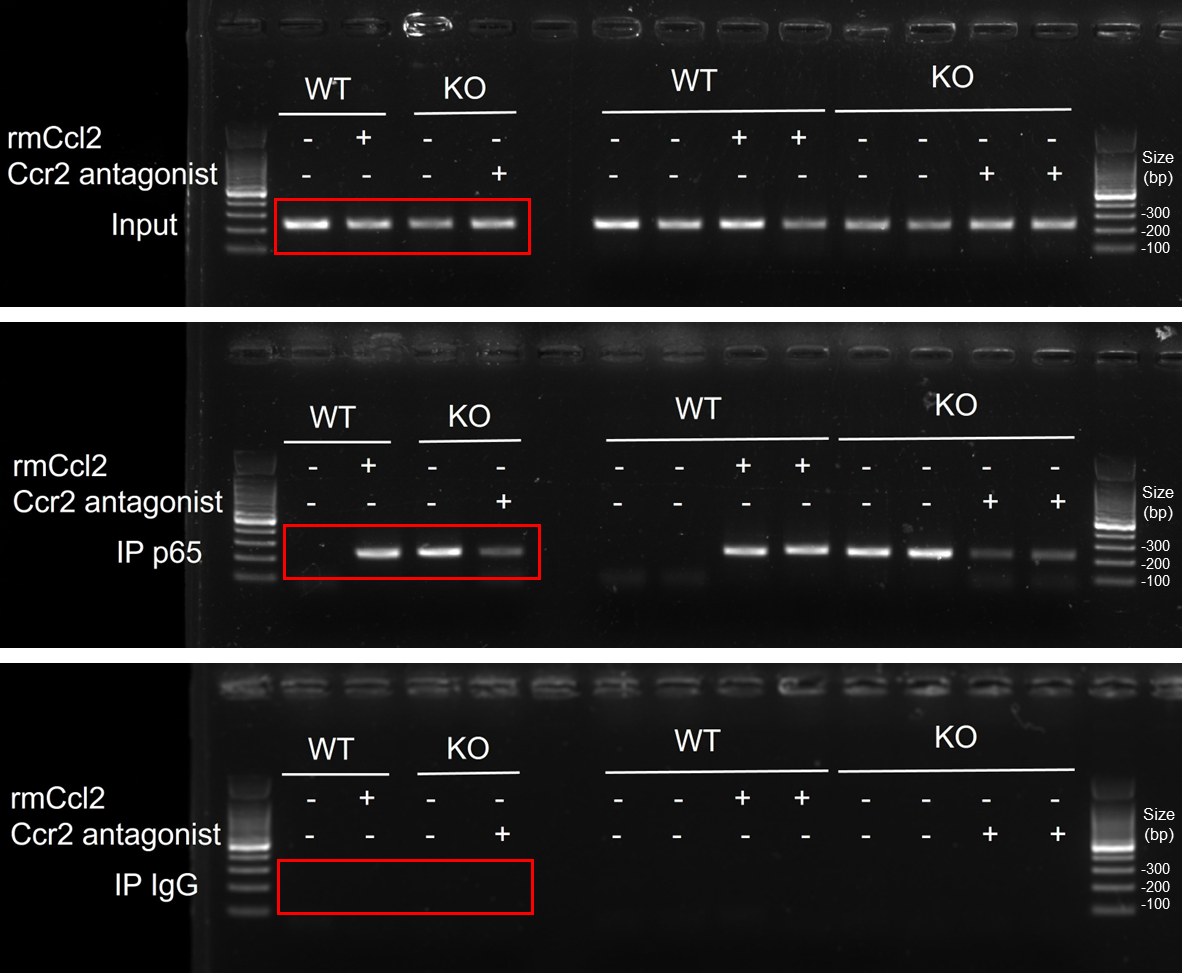

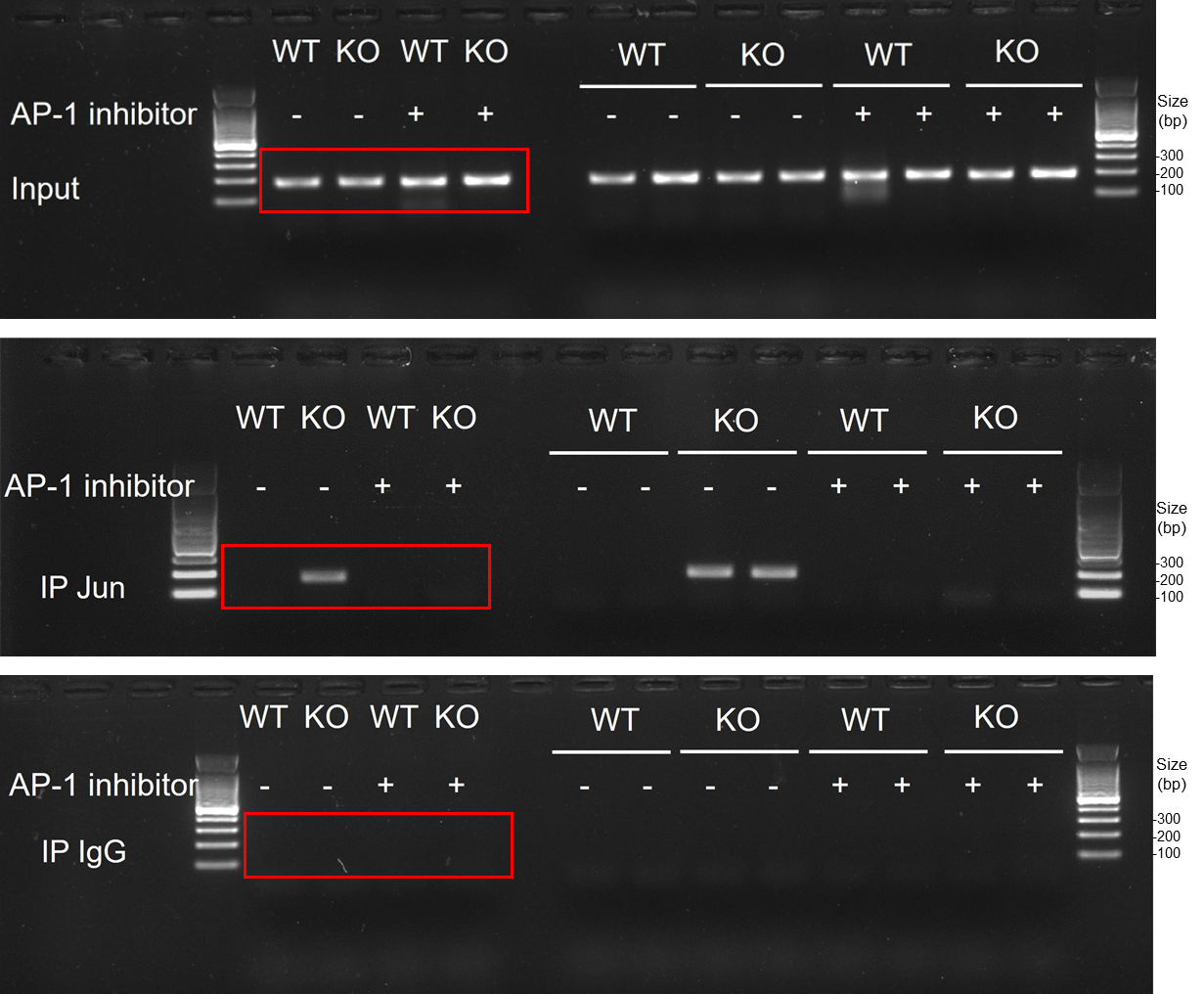
**

Original and uncropped films of agarose gel

Fig. 7h

Fig. 7r
